# A Compendium of Syngeneic, Transplantable Pediatric High-Grade Glioma Models Reveals Subtype-Specific Therapeutic Vulnerabilities

**DOI:** 10.1158/2159-8290.CD-23-0004

**Published:** 2023-04-03

**Authors:** Michael McNicholas, Antonella De Cola, Zahedeh Bashardanesh, Amelia Foss, Cameron B. Lloyd, Steven Hébert, Damien Faury, Augusto Faria Andrade, Nada Jabado, Claudia L. Kleinman, Manav Pathania

**Affiliations:** 1 Department of Oncology and Milner Therapeutics Institute, Jeffrey Cheah Biomedical Centre, University of Cambridge, Cambridge, United Kingdom.; 2 CRUK Children's Brain Tumour Centre of Excellence, University of Cambridge, Cambridge, United Kingdom.; 3 Lady Davis Research Institute, Jewish General Hospital, Montreal, Quebec, Canada.; 4 Department of Human Genetics, McGill University, Montreal, Quebec, Canada.; 5 Laboratory of Cell Biology, Center for Cancer Research, National Cancer Institute, National Institutes of Health, Bethesda, Maryland.; 6 Department of Pediatrics, McGill University, and The Research Institute of the McGill University Health Centre, Montreal, Quebec, Canada.; 7 Division of Experimental Medicine, Department of Medicine, McGill University, Montreal, Quebec, Canada.

## Abstract

Pediatric high-grade gliomas (pHGG) are lethal, incurable brain tumors frequently driven by clonal mutations in histone genes. They often harbor a range of additional genetic alterations that correlate with different ages, anatomic locations, and tumor subtypes. We developed models representing 16 pHGG subtypes driven by different combinations of alterations targeted to specific brain regions. Tumors developed with varying latencies and cell lines derived from these models engrafted in syngeneic, immunocompetent mice with high penetrance. Targeted drug screening revealed unexpected selective vulnerabilities—H3.3^G34R^/PDGFRA^C235Y^ to FGFR inhibition, H3.3^K27M^/PDGFRA^WT^ to PDGFRA inhibition, and H3.3^K27M^/PDGFRA^WT^ and H3.3^K27M^/PPM1D^ΔC^/PIK3CA^E545K^ to combined inhibition of MEK and PIK3CA. Moreover, H3.3^K27M^ tumors with PIK3CA, NF1, and FGFR1 mutations were more invasive and harbored distinct additional phenotypes, such as exophytic spread, cranial nerve invasion, and spinal dissemination. Collectively, these models reveal that different partner alterations produce distinct effects on pHGG cellular composition, latency, invasiveness, and treatment sensitivity.

**Significance:**

Histone-mutant pediatric gliomas are a highly heterogeneous tumor entity. Different histone mutations correlate with different ages of onset, survival outcomes, brain regions, and partner alterations. We have developed models of histone-mutant gliomas that reflect this anatomic and genetic heterogeneity and provide evidence of subtype-specific biology and therapeutic targeting.

*See related commentary by Lubanszky and Hawkins, p. 1516.*

*This article is highlighted in the In This Issue feature, p. 1501*

## Teaser

Models of pediatric high-grade glioma reveal genotype–phenotype associations, differences in cell type composition, and sensitivity to targeted and combination therapy approaches, making preclinical evaluation of a precision therapy approach possible.

## INTRODUCTION

Pediatric high-grade gliomas (pHGG) are lethal and incurable brain tumors occurring in children and adolescents (1–3). Current treatments are ineffective and largely palliative, underlining the need for more effective, targeted therapies (1). Half of all pHGGs, and most diffuse midline gliomas (DMG), bear driver mutations in histone 3 (H3) variants H3.1 and H3.3, exhibiting lysine-to-methionine substitution at K27 (K27M) in H3.1 or H3.3, or glycine-to-arginine/valine substitution at G34 (G34R/V) in H3.3 (4–6). H3 mutations exhibit very specific distribution patterns correlating with different ages, locations, partner alterations, and clinical outcomes (2, 7–13). H3 mutations arise in the developing brain in discrete neural progenitor populations, in which they deregulate the epigenome, stall differentiation, and increase the likelihood of tumorigenesis (11, 13–20).

**Figure 1.**
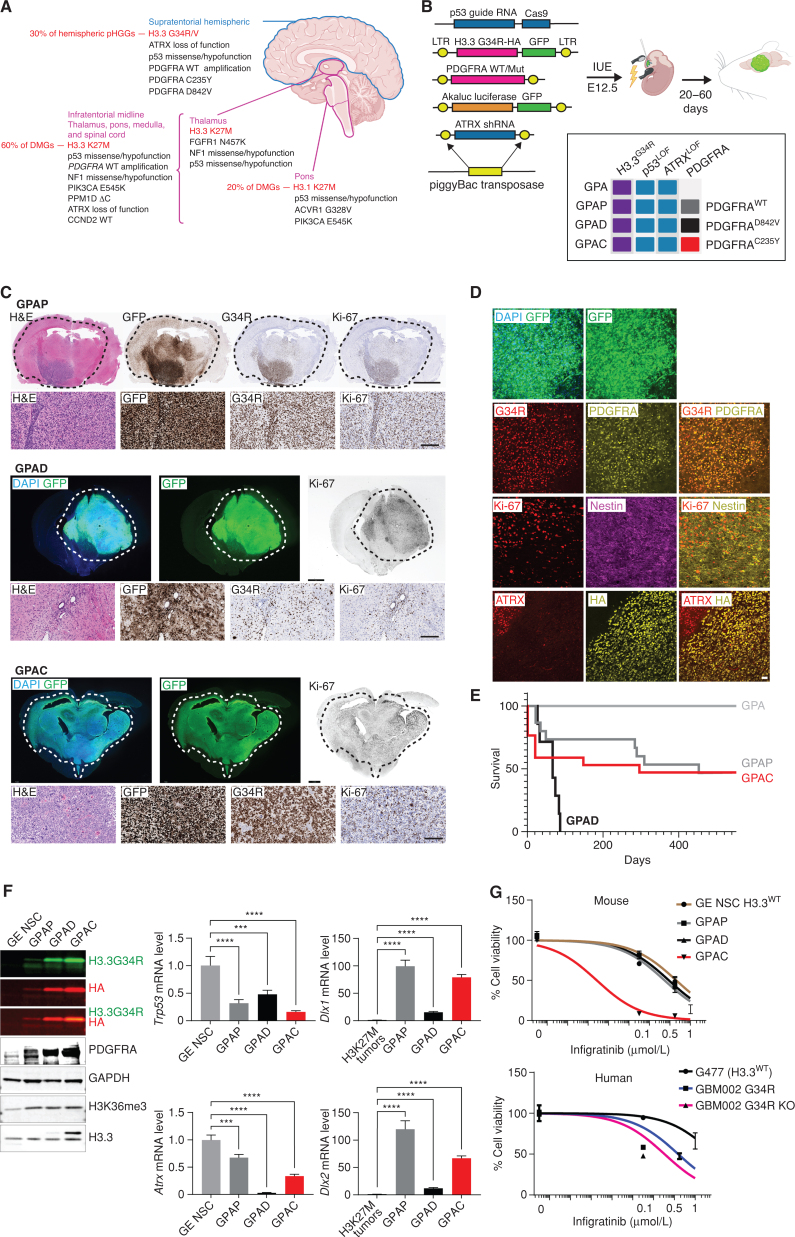
**A,** Schematic representation of cosegregating mutations in pHGGs and DMGs and their correlation with different anatomic compartments. WT, wild-type. **B,** Schematic describing *in utero* electroporation (IUE)–based H3.3^G34R^-driven brain tumor modeling. Different combinations of piggyBac and CRISPR vectors are delivered into neural stem cells (NSC) in the ganglionic eminences (GE) at E12.5. After birth, mice develop tumors representing distinct pHGG subtypes. Inset: mutation combinations represented in each H3.3^G34R^ model and the acronyms used to refer to them. E12.5, embryonic day 12.5; LOF, loss of function. **C,** Immunofluorescence and IHC detection of GFP, G34R, Ki-67, and hematoxylin and eosin (H&E) in coronal forebrain sections from tumor-bearing, symptomatic mice. Embryos were electroporated with vectors encoding (i) H3.3^G34R^, p53^LOF^, ATRX^LOF^, PDGFRA^WT^ (GPAP); (ii) H3.3^G34R^, p53^LOF^, ATRX^LOF^, PDGFRA^D842V^ (GPAD); and (iii) H3.3^G34R^, p53^LOF^, ATRX^LOF^, PDGFRA^C235Y^ (GPAC). Tumor cells are GFP^+^. Higher magnification panels below each low-magnification panel are of the tumor bulk in the ventral forebrain, i.e., striatum, which is what the GE differentiates into in adults. Scale bars, 1 mm in the immunofluorescence panels and 200 μm in the IHC panels. **D,** Immunofluorescence of tumor tissue in the ventral forebrain, i.e., striatum, to confirm the presence of cointroduced mutations and for colocalization with various markers. Tumor cells are GFP^+^, G34R^+^, HA^+^, PDGFRA^+^, and ATRX^−^. They also express higher levels of Ki-67 and Nestin. Scale bars, 50 μm. **E,** Kaplan–Meier survival curves of IUE, tumor-bearing mice carrying different combinations of mutations. GPAP (*n* = 15), GPAD (*n* = 7), and GPAC (*n* = 17). Statistical comparisons using the log-rank Mantel–Cox tests are described in Supplementary Table S2. **F,** Left: acid-extracted histone and protein lysates prepared from *ex vivo* gliomasphere (GS) lines derived from the H3.3^G34R^-driven *de novo* models (GPAP, GPAD, and GPAC). GS lines express higher levels of G34R, HA, and PDGFRA than control wild-type NSCs derived from GEs (GE NSC). Total H3.3 levels and GAPDH were used as loading controls. Middle: validation of p53 and ATRX downregulation in *ex vivo* H3.3^G34R^ GS cells by qRT-PCR. Right: validation of DLX1/2 upregulation in *ex vivo* H3.3^G34R^ GS cells by qRT-PCR. Data are presented as means ± SD from at least *n* = 3 replicates. ****, *P* < 0.0001 and ***, *P* = 0.0003 were calculated using one-way ANOVA. **G,** Dose–response curves for mouse GPAP, GPAD, and GPAC cells and normal H3.3^WT^ NSCs (top), and human G34R (GBM002), G34R knockout (KO), and H3.3^WT^ glioma cells (bottom) following treatment with infigratinib for 6 days at the indicated concentrations. Means ± SD are plotted from at least *n* = 3 replicates. Statistics are summarized in Supple­mentary Table S7.

Although H3 mutations are clonal, highly recurrent (2, 4, 6–8), and necessary for tumorigenesis (20, 21), they alone are not sufficient to induce tumorigenesis, as our previous work has shown (20). Partner alterations are needed for transformation, and they too exhibit spatiotemporal and mutually exclusive patterns of occurrence (Fig. 1A; refs. 2, 7). For instance, p53 missense mutations and truncations in PPM1D, a negative regulator of p53 itself, are often mutually exclusive (2). Another example is ATRX loss of function, which is associated with hemispheric G34R/V tumors and is less frequent in midline K27M tumors (2, 4, 9). In addition, PIK3CA mutations frequently cosegregate with H3.1^K27M^ mutations and PPM1D truncations (2). Gain-of-function alterations in growth factor receptors also co-occur with specific H3 mutations. Activating mutations in ACVR1 occur most often in H3.1^K27M^ tumors in the brainstem (7), whereas FGFR1 mutations usually occur in H3.3^K27M^ tumors in midline locations outside the brainstem (7, 22). A proportion of FGFR1-mutant tumors also harbor inactivating mutations in NF1 (2, 22). *PDGFRA* amplifications cosegregate with p53 mutations in both H3.3^K27M^ and G34R/V tumors (23–26). Uniquely, G34R/V tumors frequently acquire the C235Y mutation in the extracellular domain of PDGFRA (50% at diagnosis, 80% at relapse; ref. 13) and possess an epigenomic and transcriptomic signature mirroring interneuron progenitors arising in the ganglionic eminences. K27M tumors, on the other hand, display an early brainstem oligodendrocyte precursor cell (OPC)–like signature that arises in the lower rhombic lip (27, 28). Therefore, a specific cellular context is needed for each partner alteration's oncogenic functions, given (i) their spatiotemporal patterns, (ii) their association with specific H3 mutants, and (iii) their functional roles in signaling pathways, which are known to depend on context. Defining this context is crucial, as partners are more pharmacologically tractable and thus better precision therapy candidates than H3 mutations (29–31).

To resolve the roles of partner alterations in pHGGs while restricting tumor development to specific brain regions, we refined our *in utero* electroporation (IUE)–based approach to deliver combinations of mutations to discrete neural stem cell (NSC) niches in the embryo. We developed the first glioma model driven by H3.3^G34R^ and its associated PDGFRA^C235Y^ mutation, demonstrating that the ganglionic eminences (GE) can function as the originating progenitor niche for these tumors. In the context of K27M, we developed additional highly penetrant models driven by lower rhombic lip (LRL) delivery of partner alterations in ACVR1, PIK3CA, PPM1D, NF1, and FGFR1, which together represent the most common DMG subtypes. Uniquely, these new models do not require constitutively active PDGF/PDGFRA signaling to drive tumor development, allowing investigation of subtype-specific oncogenic mechanisms. We found that although K27M was unable to drive tumor development alone, the addition of one further mutation in the form of either p53 loss, NF1 loss, or FGFR1 mutation was sufficient to induce highly penetrant tumor development (70% or higher). We also show that specific phenotypes such as exophytic spread, cranial nerve invasion, and spinal dissemination are associated with distinct partner alterations. Single-nuclear profiling of a subset of models confirmed good correspondence with human tumors and revealed variable cell type hierarchies. Cell lines derived from these models engraft in syngeneic, immunocompetent mice with high penetrance and short latencies, and drug screening identified selective vulnerabilities. This included sensitivity to the FGFR inhibitor infigratinib in H3.3^G34R^/PDGFRA^C235Y^ cells and to a combination therapy involving the PIK3CA inhibitor alpelisib and the MEK inhibitor trametinib in *PDGFRA*^WT^-amplified and PIK3CA^E545K^/PPM1D^ΔC^-mutant cells, the latter of which was validated in patient-derived cell lines. These selective vulnerabilities can inform precision therapy development for H3-mutant pHGG subtypes.

## RESULTS

### H3.3^G34R^ and PDGFRA Alterations Drive Tumorigenesis from the GE, with PDGFRA^C235Y^-Derived Cells Uniquely Sensitive to the FGFR Inhibitor Infigratinib

To systematically assess the effect of histone mutations in combination with other alterations seen in patients, we optimized a delivery system to introduce these mutations at embryonic day 12.5 (E12.5) into the mouse dorsal or ventral pallium, targeting the cortex or GE, respectively, using IUE of piggyBac transposons and CRISPR vectors (Fig. 1B; Supplementary Table S1; ref. 32). Transient expression of the piggyBac transposase and Cas9 ensures a brief period of clonal induction, allowing small populations of genetically recombined and spatially restricted premalignant progenitors to give rise to gliomas in an immunocompetent environment. The vector carrying the histone mutant expressed GFP downstream from a modified 2A peptide (PQR), and the mutant histone itself was C-terminally tagged with an HA label, allowing immunochemical detection of tumor cells with GFP and the mutant histone with HA (Supplementary Fig. S1A; ref. 33). The vectors carrying the overexpressed partner alterations were similarly tagged with an EBFP/V5 label (Supplementary Fig. S1B). Vector concentrations were optimized for cotransfection efficiency considering the size of each plasmid (NEBioCalculator). Finally, to enable noninvasive bioluminescence imaging (BLI), we engineered and codelivered a vector expressing Akaluc luciferase, which enables BLI in C57BL/6J mice harboring tumors in deep brain structures such as the ventral forebrain or brainstem without the need for shaving or depilation (Supplementary Fig. S1C; ref. 34). We confirmed our ability to target these anatomic locations, demonstrating that GFP-encoding vectors can be specifically and reproducibly introduced into the GE or cortex at E12.5 (Supplementary Fig. S2A–S2D).

To dissect the contributions of H3.3^G34R^, specific PDGFRA alterations, and the environmental niche of the GE *in vivo*, we created mouse models carrying H3.3^G34R^, ATRX^LOF^, and p53^LOF^ (GPA), together with PDGFRA^WT^ (GPA**P**), PDGFRA^C235Y^ (GPA**C**), or PDGFRA^D842V^ (GPA**D**; Fig. 1B; Supplementary Figs. S3A and S3B; S4A and S4B; S5A and S5B; S6). PDGFRA^WT^ was used to mimic the focal amplification of the normal gene that is often seen in these tumors, whereas PDGFRA^C235Y^ modeled the highly prevalent mutation observed specifically in H3.3^G34R^ tumors. PDGFRA^D842V^ was included as a positive control to guarantee tumor development and allow comparison with other published models (35–38).

GPAD tumors showed 100% penetrance with a median survival of 67 days, as expected for the highly oncogenic PDGFRA^D842V^ mutation (Fig. 1C–E; Supplementary Tables S2 and S3). Tumor penetrance in GPAP and GPAC mice was lower, with 55% of the mice developing neurologic symptoms and tumors (Fig. 1C–E). GPAC tumors were more aggressive and had a shorter latency, with a median survival of 296 days compared with 453 days for GPAP tumors (Supplementary Table S2 and S3). The GPA combination without PDGFRA was unable to drive tumor development (Fig. 1E; Supplementary Fig. S6). We validated the presence of each introduced mutation in all models (Fig. 1D; Supplementary Figs. S3A and S3B; S4A and S4B; S5A and S5B; and S6), confirming that codelivery of separate vectors using piggyBac IUE leads to stable integration and coexpression of each mutation in the majority of tumor cells. Histologically, H3.3^G34R^ tumors possessed both densely compacted and diffuse components, and all maintained low levels of ATRX expression (Fig. 1D; Supplementary Figs. S3A and S3B; S4A and S4B; S5A and S5B; and S6). Recapitulating human tumor biology, H3.3^G34R^ tumors displayed lower levels of OLIG2 than K27M tumors and expressed high levels of the interneuron progenitor markers DLX1 and DLX2 (Fig. 1F; ref. 13).

Introducing PDGFRA mutations into the GE induced lethality in a proportion of electroporated embryos. Codelivery of H3.3^WT^ with PDGFRA^C235Y^ or PDGFRA^D842V^ saw only 34% and 38% of pups surviving to weaning, respectively (Supplementary Fig. S7). This decrease in survival was not observed with PDGFRA^WT^ (Supplementary Fig. S7). Interestingly, survival was higher when H3.3^G34R^ was codelivered with PDGFRA^C235Y^ into the GE, with 65% of H3.3^G34R^/PDGFRA^C235Y^ pups surviving to weaning (Supplementary Fig. S7). This suggests that H3.3^G34R^ can alleviate some of the lethality induced by PDGFRA^C235Y^, allowing survival of a greater proportion of embryos expressing this combination of mutations until H3.3^G34R^/PDGFRA^C235Y^ cells undergo transformation. This effect is also niche-specific, occurring only in the GE and not in the cortex (Supplementary Fig. S7).

We generated gliomasphere (GS) cell lines from symptomatic GPAP, GPAD, and GPAC mice in serum-free media. GS cells expressed HA-tagged H3.3^G34R^ and maintained ATRX and p53 downregulation with PDGFRA overexpression, as expected (Fig. 1F). We assessed the sensitivity of GPAP, GPAD, and GPAC GS cells to eight different small-molecule inhibitors, each targeting a different pathway: PDGFRA (avapritinib), PIK3CA (alpelisib), FGFR1 (infigratinib), MEK (trametinib; ref. 39), MDM2 (idasanutlin; ref. 40), NAD biosynthesis (FK866; ref. 41), histone lysine methylation (GSK-J4; ref. 42), and histone acetylation (corin; ref. 43). Although GPAC cells were largely insensitive to the PDGFRA inhibitor avapritinib (Supplementary Fig. S8; ref. 44), they displayed a remarkable, submicromolar sensitivity to the FGFR inhibitor infigratinib (Fig. 1G). This sensitivity to infigratinib was specific and more pronounced in GPAC cells compared with GPAD, GPAP, and wild-type neurospheres, indicating a unique selectivity for the PDGFRA^C235Y^ mutation. We confirmed human tumor-derived GBM002 cells that carry the G34R mutation are also more sensitive to infigratinib than H3.3^WT^ glioma cells (Fig. 1G).

Collectively, these data describe the first H3.3^G34R^ models developed by targeting the GE in embryonic mice, experimentally validating the GE as a niche capable of giving rise to these tumors. Three models were developed, driven by H3.3^G34R^ and three different PDGFRA alterations. Additionally, the GPAC model describes the first GE-targeted, H3.3^G34R^-mutant model driven by the recently discovered PDGFRA^C235Y^ mutation. Finally, we demonstrate that GPAC tumors are unexpectedly sensitive to the FGFR inhibitor infigratinib, which may offer alternative strategies to sensitize these tumors to treatment, known to be insensitive to traditional PDGFRA inhibitors.

### K27M Models Driven by ACVR1, PIK3CA, PPM1D, NF1, and FGFR1 Are Highly Penetrant

**Figure 2.**
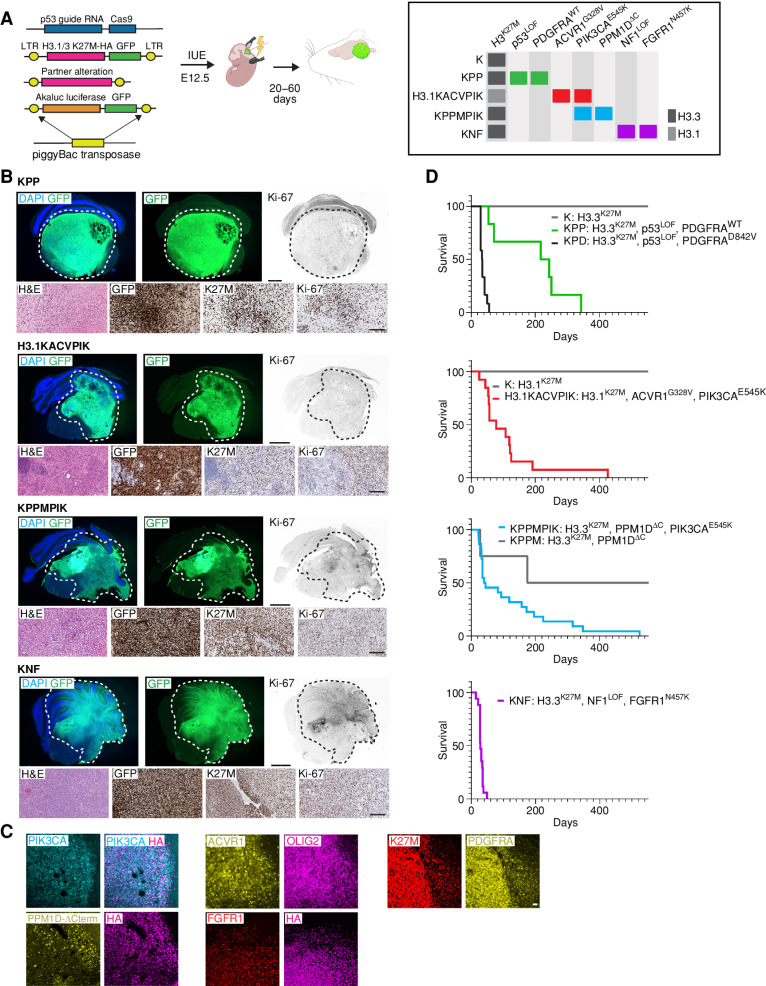
**A,** Schematic describing IUE-based H3^K27M^-driven brain tumor modeling. Different combinations of piggyBac and CRISPR vectors are delivered into NSCs lining the LRL at E12.5. After birth, mice develop tumors representing distinct DMG subtypes. Inset: mutation combinations represented in H3^K27M^ models and the acronyms used to refer to them. **B,** Immunofluorescence and IHC detection of GFP, K27M, Ki-67, and hematoxylin and eosin (H&E) in coronal hindbrain sections from tumor-bearing, symptomatic mice. Embryos were electroporated with vectors encoding (i) H3.3^K27M^, p53^LOF^, PDGFRA^WT^ (KPP); (ii) H3.1^K27M^, ACVRA^G328V^, PIK3CA^E545K^ (H3.1KACVPIK); (iii) H3.3^K27M^, PPM1D^ΔC^, PIK3CA^E545K^ (KPPMPIK); and (iv) H3.3^K27M^, NF1^LOF^, FGFR1^N457K^ (KNF). Tumor cells are GFP^+^. Higher magnification panels below each low-magnification panel are of the tumor bulk in the brainstem, i.e., pons and medulla, which are directly below the cerebellum and fourth ventricle. Scale bars represent 1 mm in the immunofluorescence panels and 200 μm in the IHC panels. **C,** Immunofluorescence of tumor tissue sections in the pons or medulla to confirm the presence of cointroduced mutations and for colocalization with various markers. PIK3CA and PPM1D are coexpressed in HA^+^ KPPMPIK tumor cells (left); ACVR1 and OLIG2 colocalize in H3.1KACVPIK tumors (middle, top); FGFR1 is upregulated in HA^+^ KNF tumor cells (middle, bottom); and PDGFRA is expressed in K27M^+^ KPP tumor cells (right). Scale bars, 50 μm. **D,** Kaplan–Meier survival curves of IUE, tumor-bearing mice carrying different combinations of mutations. KPP (*n* = 6), H3.1KACVPIK (*n* = 13), KPPMPIK (*n* = 22), and KNF (*n* = 17). Statistical comparisons using the log-rank Mantel–Cox tests are described in Supplementary Table S2.

Most K27M DMGs harbor partner alterations, with the majority possessing 3 to 10 additional mutations from over 30 recurrently altered genes (2, 45). To assess which alterations can cooperate with K27M to induce tumorigenesis, we first evaluated mutations most frequently found in human DMGs. We focused on modeling four main subtypes driven by combinations of distinct partners (Fig. 2A). Three of these models (KPP, H3.1KACVPIK, and KPPMPIK) represent the most common DMG subtypes, harboring partner alterations in PDGFRA, ACVR1, PIK3CA, and PPM1D. Additionally, we modeled a rarer midline subtype that occurs outside the hindbrain, in the thalamus (7, 22), harboring NF1^LOF^ and FGFR1^N457K^ (KNF; Fig. 2A). FGFR1 mutations and NF1^LOF^ represent interesting counterpoints to p53^LOF^ and PDGFRA alterations, allowing evaluation of different signaling pathways that have thus far not been investigated in the context of K27M.

We introduced these mutation combinations into the LRL of E12.5 mice using the piggyBac transposon system, as before. Bicistronic HA-tagged H3.1/3^WT^ or H3.1/3^K27M^-GFP vectors were delivered together with vectors expressing various partner alterations, the Akaluc vector to allow BLI, and transient piggyBac transposase and Cas9 to mediate permanent recombination. We confirmed our ability to target the correct anatomic location, demonstrating specific and reproducible induction of GFP expression in the hindbrain at P0 and at weaning (Supplementary Fig. S9A–S9C).

High-grade, 100% penetrant tumors developed with all four combinations (Fig. 2B–D), with large GFP^+^ lesions apparent in the hindbrains of symptomatic mice (Supplementary Figs. S10A and S10B; S11A and S11B; S12A and S12B; S13A and S13B; S14; and S15). We verified the presence of each partner alteration in these tumor models (Fig. 2C; Supplementary Figs. S10A and S10B; S11A and S11B; S12A and S12B; S13A and S13B; S14; and S15). Tumors were diffusely infiltrative, and GFP^+^ tumor cells clearly colocalized with H3^K27M^ and Ki-67 and expressed high levels of neural stem/progenitor cell markers OLIG2 and Nestin (Fig. 2C; Supplementary Figs. S10A and S10B; S11A and S11B; S12A and S12B; S13A and S13B; S14; and S15). GFP^+^/HA^+^ cells in tumors displayed a marked reduction in H3K27me3 levels, as expected in cells expressing the K27M mutation (Supplementary Figs. S10A and S10B; S11A and S11B; S12A and S12B; S13A and S13B; S14; S15). These data highlight the similarity of these models to human tumors, as these histologic features are used in the clinic to differentiate DMGs from other brain tumor entities. Each mutation combination also demonstrated differences in the latency of tumor progression, as measured by the length of time needed for the development of brain tumor–related neurologic symptoms requiring euthanasia (Fig. 2D; Supplementary Tables S2 and S3). KNF tumor-bearing mice demonstrated the shortest median survival at 28 days, followed by KPPMPIK (42 days), H3.1KACVPIK (65 days), and KPP (230 days).

These data show that p53^LOF^ and PDGFRA^WT^, PIK3CA^E545K^, and PPM1D^ΔC^ (PPM1D^W420*^, orthologous to human PPM1D^W427*^, the most frequent truncation in ref. 2), and NF1^LOF^ and FGFR1^N457K^ are sufficient to induce DMG-like tumors in the context of H3.3^K27M^. In addition, in the context of H3.1^K27M^, ACVR1^G328V^ and PIK3CA^E545K^ are able to induce transformation. Taken together, these four mutation combinations induce brainstem tumors in mice that recapitulate the genetic and histologic characteristics of DMG subtypes, as well as their lethality. Importantly, in contrast to previously published H3^K27M^-driven models (21, 46–49), the models described here are the first and only brainstem-targeted models that do not rely on constitutively active PDGFRA or PDGF overexpression to drive tumor development while maintaining high penetrance (31). Exogenously high levels of the ligand or constitutive activation of PDGFRA is rare in the context of H3^K27M^ in human tumors and can confound efforts to identify selective targets for precision therapy, as these can be oncogenic independently of H3^K27M^. The models described here allow evaluation of the unique tumor biology produced by specific partner alterations and investigation of their selective therapeutic targeting without the confounding presence of PDGF overexpression or mutant PDGFRA.

### FGFR1 and NF1 Mutations Promote an Early OPC Tumor Signature, while p53 Loss with PDGFRA Amplification Fuels Myeloid Infiltration

**Figure 3.**
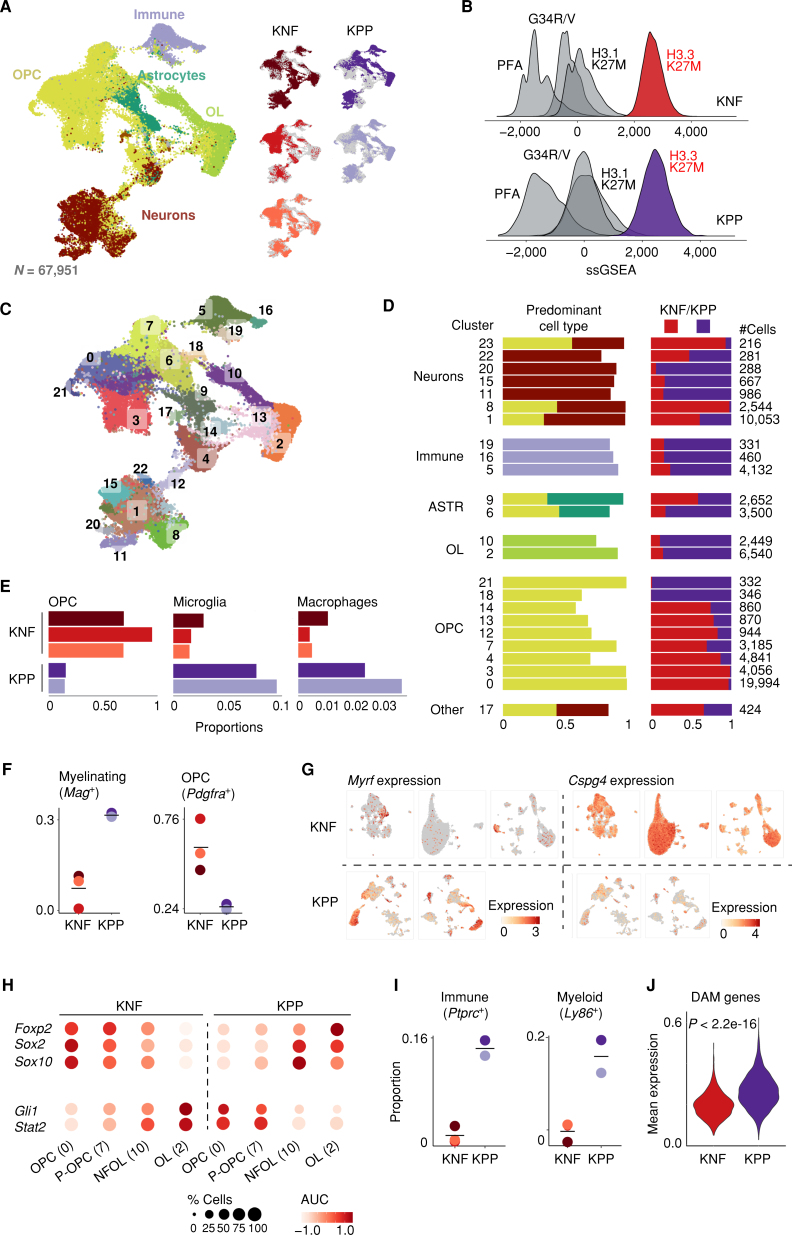
**A,** Uniform manifold approximation and projection (UMAP) of KNF and KPP integration colored by cell type annotation summarized in Supplementary Table S5 (left) and by replicate (right). Cells that did not meet quality control thresholds, as determined from individual sample analysis, and cells with no cell type consensus are not plotted. OL, oligodendrocyte. **B,** Distribution of ssGSEA scores per cell for human tumor signatures in OPC-projected cells of KNF samples (top) and KPP samples (bottom). Signatures were derived from differential expression of bulk RNA-seq data for tumor subtypes (see Methods). *N* = 34,326 cells in KNF; *N* = 3,892 cells in KPP. **C,** UMAP as in **A** with cells colored by cluster. **D,** Predominant cell types per cluster of KNF and KPP integration (left), corresponding proportion of each genotype within the cluster (middle), and number of cells per cluster (right). Only cells passing QC and with a cell type consensus are considered. Cell types comprising at least 30% of the cluster are shown. ASTR, astrocyte. **E,** Proportion of cells per replicate annotated as OPC (left), microglia (middle), and macrophages (right). **F,** Proportion of cells with detected *Mag* (left) and *Pdgfra* (right). **G,** UMAPs of each replicate from individual sample analysis with cells colored by normalized expression of *Myrf* (left) and *Cspg4* (right). **H,** Mean inferred TF activity scores (scaled) and percent of cells with activity of TF shown for clusters 0, 7, 10, and 2, with predominant cell types of OPC, proliferating OPC (P-OPC), newly forming oligodendrocytes (NFOL), and mature oligodendrocytes, respectively; cluster number is indicated in parenthesis. Representative TFs with differing activity patterns between genotypes are shown. **I,** Proportion of cells with detected *Ptprc* (left) and *Ly86* (right). **J,** Mean expression of DAM signature genes in immune cluster 5, with cells grouped by genotype. *P* < 2.2e-16.

To define the transcriptional landscape of H3.3^K27M^ tumors at single-cell resolution, we profiled the KNF (*n* = 3), KPP (*n* = 2), and KPPMPIK (*n* = 2) models using 10x Chromium single-nuclei RNA sequencing (snRNA-seq). In total, we obtained 137,695 cells passing quality control with an average of 2,255 unique molecular identifiers (UMI) per cell (KNF *n* = 93,960; KPP *n* = 31,479; KPPMPIK *n* = 12,256; Supplementary Tables S4–S6). After quality control, dimension reduction, and clustering, we first distinguished clusters of immune cells from cells of neuroectodermal origin based on canonical markers (Supplementary Fig. S16A–S16C). Next, we performed an automated annotation at the individual cell level using a consensus of four classifiers trained on a single-cell reference atlas of the developing brain for neuroectodermal cells (27) and on a single-cell reference of CD45^+^ mouse brain cells for immune cells (ref. 50; Supplementary Fig. S16D and S16E). This analysis identified well-defined populations of cells along the oligodendroglial lineage, as well as other cell types including astrocytes and vascular, neuronal, and immune cells (Supplementary Fig. S16E). Finally, we corrected for batch effects using Harmony, subsampling KNF replicates to avoid biases resulting from the large differences in cell numbers. This resulted in well-integrated datasets with biological replicates showing similar profiles (Fig. 3A; Supplementary Fig. S17A and S17B).

To assess the relevance of these models to study human disease, we performed single-sample gene set enrichment analysis (ssGSEA) in each individual cell, assessing gene signatures specific for K27M and G34R/V human tumors or posterior fossa ependymoma (PFA) as controls. For all H3.3^K27M^ mouse models, OPC-like cells showed a very specific enrichment for H3.3^K27M^ signatures (Fig. 3B; Supplementary Fig. S17C), significantly higher than for H3.1^K27M^, H3.3^G34R^, and PFA signatures, confirming that their molecular profiles recapitulate human tumor biology.

We next compared cell compositions of the KNF and KPP models to study the effects of the rarer, thalamus-specific NF1^LOF^ and FGFR1^N457K^ mutations in relation to the more frequently brainstem-specific PDGFRA^WT^ amplification and p53^LOF^ context (Fig. 3B–D). First, we observed a marked difference in cellular hierarchy between the two phenotypes, with the KNF models predominantly composed of OPC-like cells, whereas the KPP models showed a different cellular composition with more mature-like cells predominating (Fig. 3E–G). Reconstruction of gene regulatory networks using SCENIC (51) revealed a different dynamic of transcription factor (TF) activation along differentiation. For the oligodendroglial lineage, several TFs active in early OPC-like cells in the KNF models (e.g., *Foxp2* and *Sox17*) were activated in later differentiation stages (myelinating oligodendrocyte-like cells) in the KPP model (Fig. 3H; Supplementary Fig. S17D). Conversely, *Maf*, *Gli1*, and *Bcl11*, among others, showed later activation in the KNF genotype. Second, KPP models showed much higher levels of myeloid infiltration (Fig. 3F, I, and J), with higher proportions of cells expressing pan-immune and myeloid markers (Fig. 3I). Furthermore, microglia clusters in these models showed increased expression of disease-associated microglia (DAM) signature genes (Fig. 3J). We also profiled KPPMPIK using snRNA-seq, which displayed more similarity to KPP than KNF tumors, with fewer immature OPCs and more mature-like cells (Supplementary Fig. S17A–S17D). Altogether, these results underscore the distinct downstream effects of NF1^LOF^ and FGFR1^N457K^ on cell-intrinsic programs, as well as on cell-extrinsic factors such as the tumor immune microenvironment.

### p53^LOF^, NF1^LOF^, FGFR1^N457K^, and CCND2^WT^ Are Sufficient to Induce Transformation in the Context of K27M

**Figure 4.**
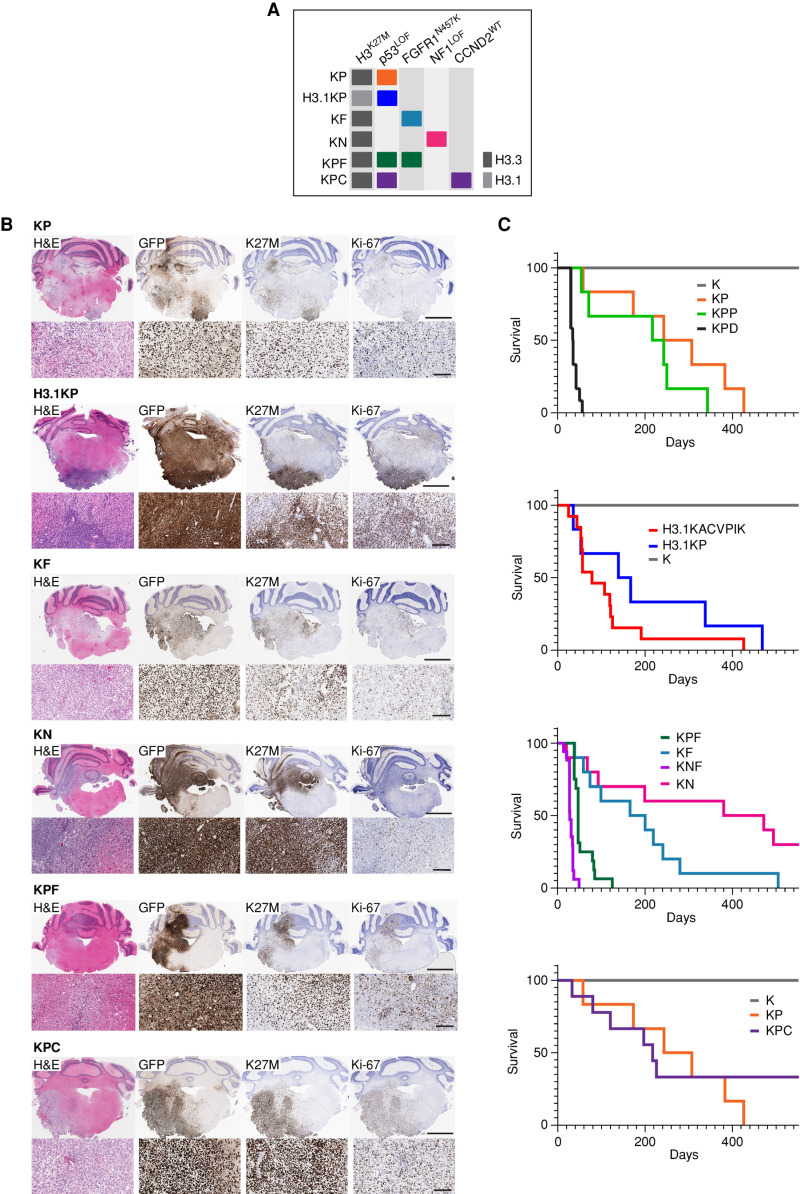
**A,** Mutation combinations represented in H3^K27M^ models and the acronyms used to refer to them. **B,** IHC detection of hematoxylin and eosin (H&E), GFP, K27M, and Ki-67 in coronal hindbrain sections from tumor-bearing, symptomatic mice. Embryos were electroporated with vectors encoding (i) H3.3^K27M^, p53^LOF^ (KP); (ii) H3.1^K27M^, p53^LOF^ (H3.1KP); (iii) H3.3^K27M^, FGFR1^N457K^ (KF); (iv) H3.3^K27M^, NF1^LOF^ (KN); (v) H3.3^K27M^, p53^LOF^, FGFR1^N457K^ (KPF); and (vi) H3.3^K27M^, p53^LOF^, CCND2^WT^ (KPC). Tumor cells are GFP^+^. Higher magnification panels below each low-magnification panel are of the tumor bulk in the brainstem, i.e., pons and medulla, which are directly below the cerebellum and fourth ventricle. Scale bars, 2 mm in the low-magnification panels and 200 μm in the high-magnification panels. **C,** Kaplan–Meier survival curves of IUE, tumor-bearing mice carrying different combinations of mutations. KP (*n* = 6), H3.1KP (*n* = 6), KF (*n* = 10), KN (*n* = 7), KPF (*n* = 16), and KPC (*n* = 9). Statistical comparisons using the log-rank Mantel–Cox tests are described in Supplementary Table S2.

Next, we evaluated H3^K27M^-specific partner alterations individually and in combination with other rarer partners to identify those that had transformation potential. H3 mutations arrest progenitors in a proliferative, preneoplastic state but cannot induce transformation alone, thus we interrogated which additional mutations are needed at minimum to induce tumor development. We focused on mutations linked to discrete brain regions and key biological pathways altered in DMGs, including mutations specific to thalamic and brainstem tumors and affecting receptor tyrosine kinases; BMP, PI3K/mTOR, and MAPK signaling; the cell cycle; and DNA repair. Therefore, we investigated alterations in FGFR1, ACVR1, PIK3CA, NF1, CCND2, PPM1D, and p53 in combinations of two or three coexpressed mutations, and in the context of two different histone mutations (H3.1^K27M^ and H3.3^K27M^; Fig. 4A). These alterations, together with the four subtype models described above, reflect a large proportion of the genetic heterogeneity seen in DMGs while also representing tumor subtypes that do not rely on PDGF/PDGFRA signaling.

When delivered into the LRL at E12.5, p53^LOF^ (KP), FGFR1^N457K^ (KF), and NF1^LOF^ (KN) were sufficient to induce tumorige­nesis as the sole second hit together with H3.3^K27M^, with median survivals of 275 days, 209.5 days, and 199 days, respectively (Fig. 4B and C; Supplementary Figs. S18A and S18B; S19A and S19C; and S20A and S20B). However, PPM1D^ΔC^ or PIK3CA^E545K^ was not able to drive tumor development in our system when delivered as the only partners with H3.3^K27M^. Only one of four H3.3^K27M^/PPM1D^ΔC^ mice included in the survival curve in Fig. 2D developed a brain tumor; the rest developed hydrocephaly or other non–brain tumor–related conditions requiring euthanasia. In the context of H3.1^K27M^, p53^LOF^ could function as the sole additional transforming hit with a median survival of 153 days (H3.1KP; Fig. 4B and C; Supplementary Fig. S21A and S21B) but ACVR1^G328V^ could not without cointroduction of PIK3CA^E545K^ as a third hit. In the context of H3.3^K27M^ and p53^LOF^, FGFR1^N457K^ (KPF) and CCND2^WT^ (KPC) were able to accelerate tumor development as third hits, with median survivals of 47 days and 217 days, respectively (Fig. 4B and C; Supplementary Figs. S22A–S22C and S23A and S23B). The KP, H3.1KP, KF, and KPF models were 100% penetrant, whereas the KN and KPC models were 70% and 65% penetrant, respectively (Fig. 4C; Supplementary Table S2 and S3). GFP^+^ cells in these tumors colocalized with K27M and Ki-67 and expressed high levels of OLIG2 (Fig. 4B; Supplementary Figs. S18A and S18B; S19A and S19C; S20A and S20B; S21A and S21B; S22A–S22C; and S23A and S23B). We confirmed the presence of the histone mutation and partner alterations in each model (Supplementary Figs. S18A and S18B; S19A–S19C; S20A and S20B; S21A and S21B; S22A–S22C; and S23A and S23B). The different combinations of partner alterations produced different latencies of tumor development, as shown by their Kaplan–Meier survival curves depicted in Fig. 4C and summarized in Supplementary Tables S2 and S3.

Of note, H3.1^K27M^ and H3.3^K27M^ expression alone (Supplementary Figs. S14 and S15) and H3.3^WT^/p53^LOF^, H3.3^WT^/NF1^LOF^, H3.3^WT^/FGFR1^N457K^, and H3.3^WT^/PPM1D^ΔC^/PIK3CA^E545K^ (Supplementary Table S3) were not able to induce tumor development in mice when introduced into the LRL at E12.5. Although our previous work described H3.3^K27M^- and p53^LOF^-driven gliomagenesis in both the cortex and hindbrain (20), the KP model described here is the first 100% penetrant model of this two-hit combination in the brainstem. A previous H3.3^K27M^/p53^LOF^ transgenic model produced cerebellar rather than brainstem tumors (21). In contrast, our approach restricts tumor development to the brainstem, where these tumors occur in patients. In addition, H3.1KP is the first model to describe H3.1^K27M^'s oncogenic capacity with just one additional alteration: p53^LOF^. Finally, mutations affecting cell-cycle components are present in 25% of pHGGs, with *CCND2* amplifications being the most common in DMG (2). Thus, the KPC model is unique in allowing evaluation of cell-cycle checkpoint deregulation in the pathogenesis of DMG.

We also compared the H3.3^K27M^, p53^LOF^, and PDGFRA^WT^ (KPP) model to iterations that included ATRX^LOF^ and PDGFRA^D842V^ to identify unique effects of these partners. We compared the KPP model to models harboring the constitutively active PDGFRA^D842V^ mutation, as this mutation and another similarly constitutively active PDGFRA mutation (PDGFRA^V544ins^) have been utilized in recently published models of H3^K27M^ and H3.3^G34R^ gliomas (21, 38, 48, 52). We also wanted to evaluate whether ATRX^LOF^ affected tumor latency, as this alteration is found in a proportion of brainstem tumors. We therefore modeled (i) H3.3^K27M^, p53^LOF^, and PDGFRA^D842V^ (KPD); (ii) H3.3^K27M^, p53^LOF^, ATRX^LOF^, and PDGFRA^WT^ (KPAP); and (iii) H3.3^K27M^, p53^LOF^, ATRX^LOF^, and PDGFRA^D842V^ (KPAD; Fig. 4B and C; Supplementary Figs. S24A and S24B; S25A and S25B; and S26). As in KPP, we found KPD, KPAP, and KPAD tumors were 100% penetrant in the brainstem, with median survivals of 34.5 days, 207 days, and 45 days, respectively, in contrast to KPP's 230-day median survival (Supplementary Tables S2 and S3). It is important to note that H3.3^WT^/PDGFRA^D842V^ expression produced tumors with a similar median survival to H3.3^K27M^/PDGFRA^D842V^ (34.5 days vs. 37 days), and the two were not significantly different (Supplementary Tables S2 and S3). This suggests that PDGFRA^D842V^ is oncogenic regardless of H3^K27M^ expression in our system and may obscure the role of the histone mutation and other partner alterations.

These data demonstrate that the K27M histone mutation and at least one additional partner are necessary for tumorigenesis, and that neither the histone mutation alone nor the partner alterations in the context of H3.3^WT^ are sufficient. In the context of H3.3^K27M^ and H3.1^K27M^, p53^LOF^, NF1^LOF^, FGFR1^N457K^, and CCND2^WT^ can act as second and third hits capable of inducing and/or accelerating tumorigenesis. These data also demonstrate that the two-hit combinations of H3.1^K27M^/ACVR1^G328V^, H3.3^K27M^/PIK3CA^E545K^, and H3.3^K27M^/PPM1D^ΔC^ are not oncogenic using this approach, potentially requiring additional partners for transformation. Altogether, these data describe nine minimal, two- and three-hit mutation combinations (KP, H3.1KP, KN, KF, KPF, KPC, KPD, KPAP, and KPAD) capable of inducing tumor development in the context of H3^K27M^. Six of these models are able to transform without coexpression of PDGF/PDGFRA and therefore represent valuable preclinical systems reflecting patient alterations.

### Specific Partners Associate with Spinal Dissemination, Exophytic Spread, and Cranial Nerve Invasion

**Figure 5.**
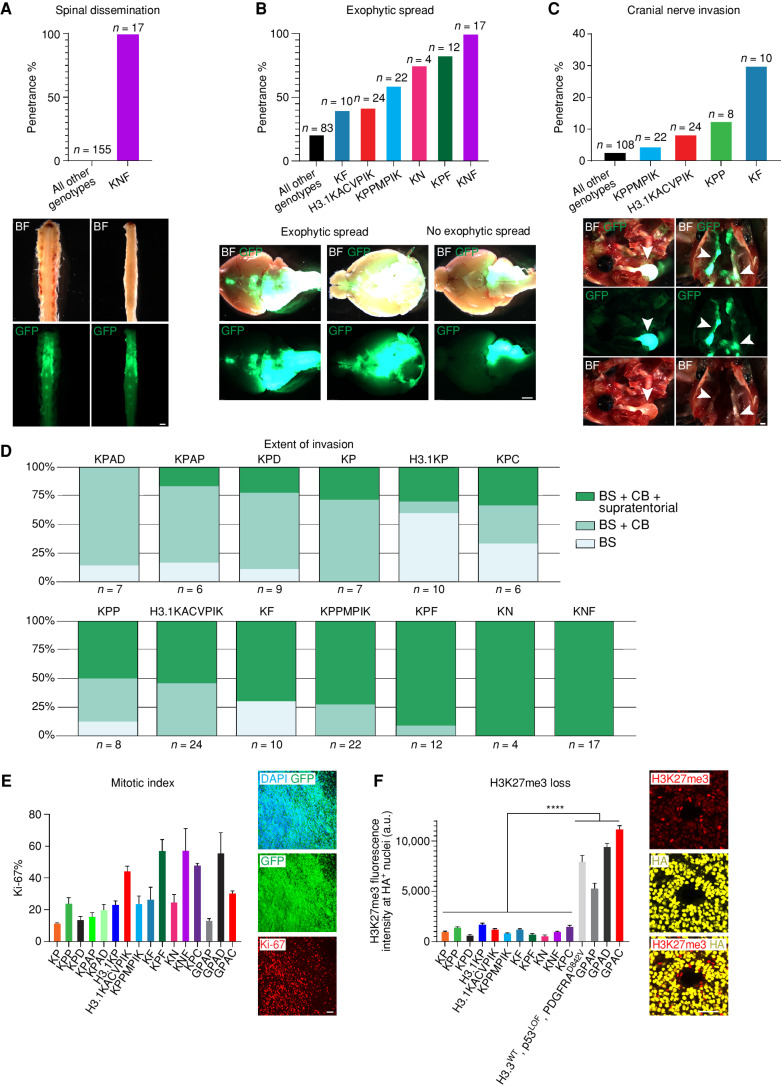
**A,** Top: penetrance of spinal dissemination in tumor-bearing mice harboring H3.3^K27M^, NF1^LOF^, and FGFR1^N457K^ mutations compared with all other genotypes. Bottom: representative bright field (BF) and fluorescence (GFP) images of GFP^+^ tumor cells that have disseminated into the thoracic spinal cord. Scale bars, 1 mm. **B,** Top: penetrance of exophytic spread in tumor-bearing mice harboring FGFR1^N457K^, PIK3CA^E545K^, PPM1D^ΔC^, and NF1^LOF^. Bottom: representative BF and fluorescence images showing exophytic spread (bottom left) or a lack of exophytic spread (bottom right) of GFP^+^ tumor cells. Scale bars, 1 mm. **C,** Top: penetrance of cranial nerve invasion in tumor-bearing KF, KPP, H3.1KACVPIK, and KPPMPIK mice. Bottom: representative BF and fluorescence images showing cranial nerve invasion of GFP^+^ tumor cells (indicated by white arrows). Scale bars, 1 mm. **D,** Analysis of the extent of invasion of H3.1/3^K27M^-driven tumors into different anatomic regions (CB, cerebellum; BS, brainstem). **E,** Immunofluorescence and quantification of Ki-67^+^/GFP^+^ cells in H3.1/3^K27M^- and H3.3^G34R^-driven tumors. GFP^+^ tumor cells have a mitotic index of 10 or higher. Data, mean ± SEM. Representative images showing DAPI, GFP, and Ki-67 immunofluorescence levels in a brainstem tumor are shown on the right. Scale bars, 50 μm. **F,** Quantification of H3K27me3 immunofluorescence signal intensity in HA^+^ nuclei in tumors. Data, mean ± SEM; ****, *P* < 0.0001 was calculated using the one-way ANOVA test. Representative images of H3K27me3 and HA staining in a brainstem tumor are shown on the right. Scale bars, 50 μm. a.u., arbitrary units.

Further phenotypic characterization of these models revealed intriguing differences in spinal dissemination, exophytic spread or blood vessel co-option, cranial nerve invasion, and parenchymal invasion. Spinal dissemination, and more spe­cifically thoracic spinal dissemination (rather than medullar or cervical), was exclusive to KNF mice and was very apparent, occurring in every mouse in this condition and in no other DMG model (Fig. 5A). We also observed what appears to be co-option of the ventral blood vessel tracks of the brain, sometimes referred to as exophytic spread, in 40% to 80% of mice harboring FGFR1^N457K^, PIK3CA^E545K^, PPM1D^ΔC^, and NF1^LOF^ (Fig. 5B). Invasion into highly myelinated cranial nerves was also a noteworthy phenotype, occurring most frequently in the KF, KPP, H3.1KACVPIK, and KPPMPIK conditions (Fig. 5C). In terms of invasion in brain parenchyma, FGFR1^N457K^-, NF1^LOF^-, PPM1D^ΔC^-, and PIK3CA^E545K^-containing conditions demonstrated significantly higher levels of thalamic/supratentorial and cerebellar invasion. Conditions with ATRX^LOF^ and two-hit models KP and H3.1KP displayed lower levels of invasion, with the H3.1KP model in particular being more restricted to the brainstem proper in 60% of mice of this condition (Fig. 5D).

We characterized the mitotic index and levels of H3K27me3 across the 16 models representing different pHGG subtypes (Fig. 5E and F). The mitotic index was consistently >10 across all conditions, with H3.1KACVPIK, KPF, KNF, and KPC possessing the highest mitotic activity at >40 for each (40% of GFP^+^ tumor cells being Ki-67^+^). GPAP, GPAC, and GPAD tumors had mitotic indices of 13, 30, and 55, respectively (Fig. 5E). H3K27me3 levels were significantly reduced in all models harboring the H3^K27M^ mutation, whereas H3.3^G34R^ tumors maintained high levels of this epigenetic mark (Fig. 5F). Altogether, these data describe specific phenotypes that are unique to certain partner alterations, such as spinal dissemination, exophytic spread, cranial nerve invasion, and extrapontine spread. These data also demonstrate differences in proliferation rates in models driven by different partner alterations and confirm the primary impact of the H3^K27M^ mutation in every tumor (reduced H3K27me3 levels).

### *Ex Vivo* Cells Derived from *De Novo* Models Engraft in Syngeneic, Immunocompetent Mice

**Figure 6.**
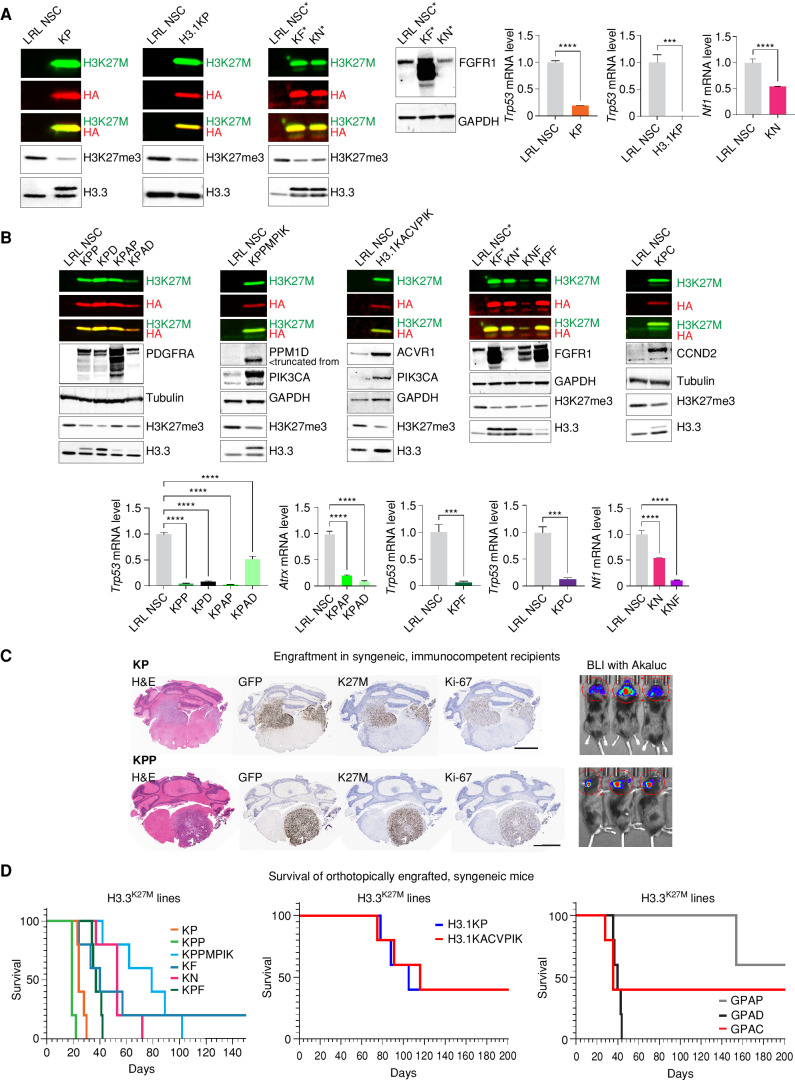
**A**, Left: acid-extracted histone and protein lysates prepared from *ex vivo* GS lines derived from the two-hit *de novo* models (KP, H3.1KP, KF, and KN) were probed for K27M, K27me3, HA, and total H3.3 levels. NSCs derived from the LRL (LRL NSC) were used as controls. Middle: validation of FGFR1 overexpression in *ex vivo* KF cells; GAPDH was used as a loading control. Right: validation of p53 and NF1 downregulation in *ex vivo* cells by qRT-PCR. Data are presented as means ± SD from at least *n* = 3 replicates. ****, *P* < 0.0001 and ***, *P* = 0.0003 were calculated using unpaired *t* test and one-way ANOVA. **B**, Left: acid-extracted histone and protein lysates prepared from *ex vivo* GS lines derived from the three- and four-hit *de novo* models (KPP, KPD, KPAP, KPAD, KPPMPIK, H3.1KACVPIK, KNF, KPF, and KPC) were probed for K27M, K27me3, HA, and total H3.3 levels. LRL NSCs were used as controls. Validation of PDGFRA, PPM1D, PIK3CA, ACVR1, FGFR1, and CCND2 overexpression in *ex vivo* GS cells; GAPDH and tubulin were used as loading controls. Right: validation of p53, ATRX, and NF1 downregulation in *ex vivo* GS cells by qRT-PCR. Data are presented as means ± SD from at least *n* = 3 replicates. ****, *P* < 0.0001 and ***, *P* = 0.0003 were calculated using unpaired *t* test and one-way ANOVA. **C,** Left: IHC detection of hematoxylin and eosin (H&E), GFP, K27M, and Ki-67 in coronal hindbrain sections from orthotopically engrafted, symptomatic mice. Mice were injected in either the pons (H3K27M) or striatum (H3.3G34R) with 150,000 to 300,000 cells from each cell GS cell line (*n* = 5 mice per condition). Tumor cells are GFP^+^. Scale bars, 2 mm. Right: BLI of brainstem tumors in orthotopically engrafted C57BL/6J mice without shaving/depilation using Akaluc imaging. **D**, Kaplan–Meier curves depicting survival of mice orthotopically injected with GS cells derived from H3.3^K27M^ (left), H3.1^K27M^ (middle), and H3.3^G34R^ (right) models. Note that the following samples are shown twice in **A** and **B**: LRL NSC, KF, and KN; these repetitions are indicated with an asterisk. The sole purpose of this repetition is to make it easier for the reader, by collating all the Western blot (WB) data describing the “two-hit” models in **A** and all the WB data describing the “three-hit or more” models in **B**.

Syngeneic, engraftable pHGG models are needed to identify novel treatment strategies targeting microenvironmental and immune interactions. These models have been difficult to generate and represent a major technical obstacle within the pHGG field, limiting the identification of novel drug targets. To develop syngeneic models, we derived a minimum of two lines from symptomatic mice for each model and grew them in serum-free media as GS. We confirmed expression of HA-tagged H3^K27M^ as well as downregulation of H3K27me3 in each GS line derived from the two-, three-, and four-hit models compared with WT LRL NSCs (Fig. 6A and B). We confirmed downregulation of p53 in KP and H3.1KP cells, and NF1 in KN cells (Fig. 6A). KF cells overexpressed FGFR1 as expected (Fig. 6A), and KPP, KPD, KPAP, and KPAD cells overexpressed PDGFRA—the latter two together with ATRX downregulation (Fig. 6B). KPPMPIK cells overexpressed the truncated form of PPM1D and PIK3CA, whereas H3.1KACVPIK cells overexpressed ACVR1 and PIK3CA (Fig. 6B). KNF and KPF cells overexpressed FGFR1 as expected, and KPF cells demonstrated knockdown of p53 levels, whereas KNF cells had diminished levels of NF1 (Fig. 6B). These data demonstrate that each model retains expression of driver and partner alterations introduced in the embryo and corroborates our *in vivo* findings regarding their coexpression (Supplementary Figs. S10–S15 and S18–S26).

Next, we evaluated the engraftment potential of these GS lines in syngeneic C57BL/6J mice. H3^K27M^ GS cells were engrafted into the pons, whereas H3.3^G34R^ tumor spheres were engrafted into the striatum. GS lines derived from every condition except for KNF were able to engraft in syngeneic mice, and most H3.3^K27M^ lines were 100% penetrant with very short latencies (Fig. 6C and D). It is unclear why KNF GS lines were unable to engraft; however, this combination of mutations produces the most aggressive tumors, with the shortest latencies in the *de novo* setting and rapid growth *in vitro*. It is possible that this condition is less adaptable to changes in growth conditions. KF, H3.1KP, H3.1KACVPIK, GPAP, and GPAC were less than 100% penetrant when orthotopically engrafted, with GPAP consistently demonstrating 40%, GPAC 60%, H3.1KP and H3.1KACVPIK 60%, and KF 80% penetrance (Fig. 6D). In contrast, KP, KPP, KPPMPIK, KN, KPF, and GPAD were 100% penetrant, with all mice developing symptoms in 20 to 100 days (Fig. 6D). Engrafted tumors appeared high-grade in hematoxylin and eosin (H&E)–stained sections and were positive for GFP, H3^K27M^, or H3.3^G34R^ and possessed high levels of Ki-67 labeling (Fig. 6D). These engraftable models, representing numerous different pHGG subtypes, will be useful tools for investigating tumor interactions with immune and other stromal cells. Importantly, introducing these mutation combinations into wild-type GE or LRL NSCs *in vitro* was not able to produce similarly engraftable GS lines, suggesting that prior transformation in an *in vivo*, immunocompetent environment is required.

### Drug Screening in Patient and Murine Model–Derived Cell Lines Reveals Sensitivity to Combination Therapy Targeting MEK and PI3K Signaling

To utilize these models for identifying subtype-specific vulnerabilities that are pharmacologically tractable, we assessed sensitivity to the same eight different small-molecule inhibitors used to assess sensitivity in GPAC cells. Each molecule targeted a different pathway component affected by one of the partner alterations: PDGFRA (avapritinib), PIK3CA (alpel­isib), FGFR1 (infigratinib), MEK (trametinib; ref. 39), MDM2 (idasanutlin; ref. 40), NAD biosynthesis (FK866; ref. 41), histone lysine methylation (GSK-J4; ref. 42), and histone acetylation (corin; ref. 43). We evaluated these inhibitors in 10 different mouse H3^K27M^ GS lines (KP, H3.1KP, H3.1KACVPIK, KPP, KPD, KPPMPIK, KNF, KPF, KN, and KF).

**Figure 7.**
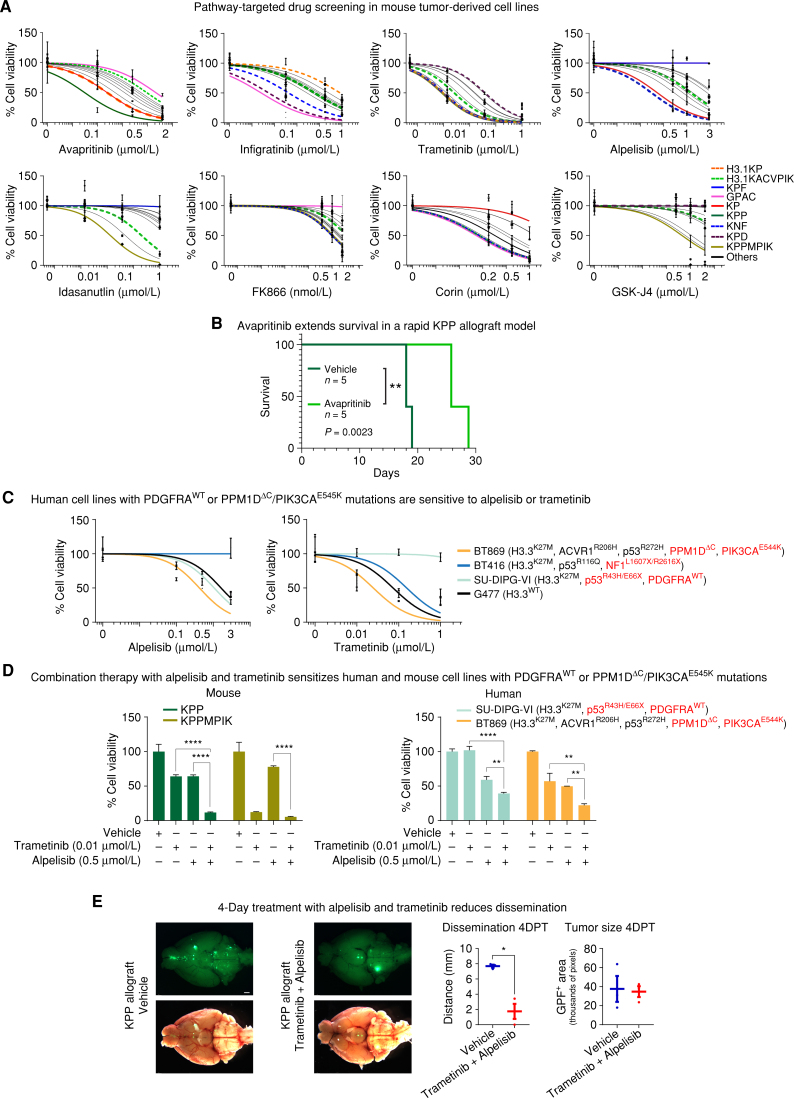
**A**, Dose–response curves for the PI3K inhibitor alpelisib, the PDGFRA^mutant^ inhibitor avapritinib, the LSD1/HDAC inhibitor corin, the KDM6A/B inhibitor GSK-J4, the MDM2 inhibitor idasanutlin, the NAMPT inhibitor FK866, the FGFR inhibitor infigratinib, and the MEK inhibitor trametinib, tested in mouse tumor-derived GS lines. Cells were treated for 3 days (avapritinib, FK-866, idasanutlin, trametinib) or 6 days (alpelisib, corin, GSK-J4, infigratinib) at the indicated concentrations. The most sensitive and the most resistant cell lines are highlighted with different colors and with dashed vs. solid lines. Means ± SD are plotted from at least *n* = 3 replicates. Statistics are summarized in Supplementary Table 7. **B**, Kaplan-Meier curves depicting survival of mice orthotopically injected with KPP cells and orally treated for 2 weeks with avapritinib vs. vehicle control. **, *P* < 0.01 was calculated using the log-rank Mantel-Cox test. Statistical comparisons are described in Supplementary Table 2. **C**, Dose–response curves for alpelisib and trametinib tested against patient-derived cell lines for 6 days at the indicated concentrations. Means ± SD are plotted from at least *n* = 3 replicates. Statistics are summarized in Supplementary Table 7. **D**, Cell viability assays using mouse GS cells and patient-derived cells following treatment with trametinib, alpelisib, and a combination of both drugs for 6 days at the indicated concentrations. Means ± SD are plotted from at least *n* = 3 replicates. ****, *P* < 0.0001; **, *P* < 0.01 were calculated using ordinary one-way ANOVA. Statistics are summarized in Supplementary Table 7. **E**, Left: representative fluorescence images of disseminating GFP^+^ tumor cells 12 days following engraftment, and 4 days following initiation of continuous intracranial delivery of alpelisib and trametinib vs. vehicle control. Scale bar, 1 mm. Right: the extent of dissemination and the size of GFP^+^ tumors following resection at 4 days after treatment. Means ± SD are plotted from at least *n* = 3 replicates. *, *P* < 0.05 was calculated using the Welch *t* test.

We identified specific sensitivity to PDGFRA inhibition by avapritinib in KP, H3.1KP, and KPP cells and to PIK3CA inhibition by alpelisib in KP, KF, KNF, and GPAC cells. Cells from KF, KNF, KPF, GPAC, and KPAD showed specific sensitivity to FGFR1 inhibition by infigratinib, whereas H3.1KP, H3.1KACVPIK, KPPMPIK, KN, and KNF cells were sensitive to MEK inhibition by trametinib (Fig. 7A; Supplementary Fig. S27). We also saw increased sensitivity to corin in KNF and H3.1KACVPIK cells (Fig. 7A; Supplementary Fig. S27). Interestingly, we noticed differences in sensitivity in KPD (PDGFRA^D842V^) compared with KPP (PDGFRA^WT^) cells, with PDGFRA^D842V^ cells being more sensitive to infigratinib and corin and less sensitive to GSK-J4, avapritinib, and alpelisib than PDGFRA^WT^ cells (Supplementary Fig. S27). This suggests that PDGFRA mutation status may be an important consideration in evaluating responses to treatment in DMG models. Statistical analyses are summarized in Supplementary Table S7. We confirmed the selective sensitivity of murine KPP cells to avapritinib *in vivo* (Fig. 7B). We orally delivered 30 mg/kg of avapritinib to syngeneic allografts over 2 to 3 weeks. Avapritinib treatment significantly extended survival in the KPP model (median survival 18 days with vehicle vs. 26 days with avapritinib, *P* = 0.0023).

To confirm these partner alteration–specific vulnerabilities in the human context, we first assessed sensitivity to alpelisib and trametinib in patient-derived cells carrying NF1 mutations (BT416), p53 mutations and PDGFRA^WT^ (SU-DIPG-VI), and PIK3CA^E544K^/PPM1D^ΔC^ (BT869). We confirmed sensitivity to alpelisib in p53-mutant/PDGFRA^WT^ and PIK3CA^E544K^/PPM1D^ΔC^ cells and to trametinib in PIK3CA^E544K^/PPM1D^ΔC^ and NF1-mutant cells (Fig. 7C). These results demonstrate that our mouse model–derived GS cells can aid in identifying therapeutic vulnerabilities unique to specific partners, which also replicate in human tumor-derived cells with similar mutation profiles.

To identify combination therapy approaches that could increase the likelihood of therapeutic success, we combined the lowest effective concentrations of trametinib and alpel­isib, and evaluated this combination in mouse and human cells carrying p53^LOF^/PDGFRA^WT^ and PIK3CA^E545K^/PPM1D^ΔC^ alterations. In cells from both species, trametinib and alpe­l­isib synergized and reduced viability with stronger effects than the application of either agent alone (Fig. 7D). Therefore, a combination therapy approach targeting MEK and PI3K signaling can sensitize DMG subtypes carrying partner alterations in p53/PDGFRA^WT^ and PPM1D/PIK3CA.

Finally, to identify agents that can limit the hallmark invasiveness of DMGs, we investigated whether the *in vivo* administration of trametinib and alpelisib could limit tumor dissemination in the short term. We administered the agents directly into the central nervous system (CNS) using mini–osmotic pumps and included a low dose of the efflux transporter inhibitor elacridar to retain high trametinib levels within the CNS. At 1 week following engraftment with KPP GS cells, when tumors demonstrated moderate levels of BLI signal, pumps containing either vehicle/elacridar or alpelisib/trametinib/elacridar were implanted into mice for continuous drug delivery into the fourth ventricle. As shown in Fig. 7E, 4 days of direct CNS delivery dramatically reduced GFP^+^ cell dissemination in the alpelisib/trametinib-treated animals relative to vehicle. Importantly, tumor volume was not affected at this early stage (Fig. 7E), indicating the specific effects on dissemination and invasion. These results highlight the utility of our *in vivo* models for evaluating pharmacologic strategies to curtail DMG invasiveness, which may make them more amenable to surgical resection. Taken together, these data demonstrate the utility of our syngeneic models for preclinical testing of treatment efficacy, providing a proof of principle for investigations now possible for many different pHGG subtypes.

## DISCUSSION

Mouse models can help identify oncogenic mechanisms and stromal co-option programs resulting from specific sets of partner alterations seen in patients. However, modeling mutant histone–driven gliomas in ways that recapitulate human disease has been challenging. These brain tumors display very precise spatiotemporally segregated patterns of occurrence, correlating with different embryonic progenitor niches. This anatomic compartmentalization has been difficult to reproduce in mice. In addition, the genetic heterogeneity of these tumors, exhibited by the many combinations of mutations observed in patient samples, is difficult to model in mice using conventional transgenic approaches. Despite these challenges, four different approaches have been successful in modeling these gliomas *in vivo* thus far. Three of these approaches are conceptually similar, combining focal, mosaic delivery of histone mutations and p53 inhibition in NSCs with either overexpression of NRAS^G12V^, PDGF ligand, or a constitutively active mutant PDGFRA (38, 46–49, 53, 54). However, increased NRAS^G12V^, PDGF, and constitutively active PDGFRA^D842V^ are potently oncogenic in isolation, thereby eclipsing the specific roles of the histone mutations and other partner alterations (23, 49, 55, 56). The fourth approach uses transgenics to conditionally drive mutant histone expression and selective loss of p53 across all NSCs while also using constitutively active PDGFRA^V544ins^ to hasten tumor development (21). This strategy leads to indiscriminate tumor development throughout the neuraxis, failing to recapitulate the anatomic compartmentalization that is a hallmark of this disease.

Here, we used IUE to resolve the roles of partner alterations while restricting tumor development to specific brain regions. In doing so, we developed a suite of syngeneic immunocompetent models driven by a range of partners in three different histone mutant contexts. Our models emulate key molecular and histologic features of human pHGGs, including diffuse infiltration, global loss of H3K27me3 and high OLIG2 expression in K27M tumors, and high DLX1/2 expression and low OLIG2 expression in forebrain G34R/V tumors. Furthermore, we demonstrate that p53^LOF^, NF1^LOF^, and FGFR1^N457K^ can function as second hits required for transformation in H3.3^K27M^ and H3.1^K27M^ tumors, and that PPM1D^ΔC^, PIK3CA^E545K^, ACVR1^G328V^, PDGFRA^WT^, and CCND2^WT^ can act as third additional hits affecting tumor malignancy and survival. We show these tumors mirror transcriptomic and epigenomic signatures of human tumors at the single-cell level, and that different partners alter cell type composition within tumors. Finally, we validate the translational potential of these models by identifying subtype-specific pharmacologic vulnerabilities in murine GS lines *in vitro* and *in vivo*, and in patient-derived cell lines with similar mutational profiles. These models and syngeneically engraftable GS lines will be useful in further studies to evaluate tumor evolution and treatment-resistance mechanisms in pHGGs.

We describe four unique models driven by just two oncogenic events: H3.1/3^K27M^ with either p53^LOF^, NF1^LOF^, or FGFR1^N457K^ mutation. In addition, we describe five additional models driven by three concurrent mutations, involving H3.3^K27M^/PPM1D^ΔC^/PIK3CA^E545K^, H3.1^K27M^/ACVR1^G328V^/PIK3CA^E545K^, H3.3^K27M^/p53^LOF^/FGFR1^N457K^, H3.3^K27M^/NF1^LOF^/FGFR1^N457K^, and H3.3^K27M^/p53^LOF^/CCND2^WT^. These nine models develop in the brainstem proper and are the only fully penetrant DMG models that are not reliant on either PDGF overexpression utilizing the RCAS system or constitutively active PDGFRA mutations, which are oncogenic on their own. The two- and three-hit FGFR1-mutant models (KF, KNF, and KPF) will aid in delineating the role of this mutation in transformation and thalamic or spinal homing in DMGs. FGFR1 alterations are also implicated in low-grade gliomas in children, although no FGFR1-driven *in vivo* models have been developed thus far. The FGFR1-mutant models described here are therefore the first to evaluate this alteration in gliomagenesis in the context of H3.3^K27M^.

The H3.1^K27M^/p53^LOF^ model is also the first to demonstrate the oncogenic capacity of H3.1^K27M^ without the need for ACVR1 or PIK3CA mutations, whereas the H3.1^K27M^/ACVR1^G328V^/PIK3CA^E545K^ model is the only such model that is fully penetrant (31). These models will be helpful for identifying H3.1^K27M^, ACVR1, and PIK3CA mutation–specific therapeutic vulnerabilities for this DMG subtype. In addition, the H3.3^G34R^ model harboring the PDGFRA^C235Y^ mutation is the first *de novo* and syngeneically engraftable model harboring this recently identified mutation. Cross-referencing PDGFRA^C235Y^-bearing tumors to tumors carrying other PDGFRA alterations in the context of H3.3^G34R^ may reveal distinct vulnerabilities that could aid in combating resistance mechanisms in these tumors. We identified infigratinib as a potent sensitizer of H3.3^G34R^/PDGFRA^C235Y^ murine tumor cells. Human cell lines carrying PDGFRA^C235Y^ mutations are currently unavailable, as are cell lines carrying many of the other partners evaluated in this study. This further emphasizes the usefulness of the models we present here, as the only material available to investigate the biological and therapeutic implications of these mutations. Finally, GS lines derived from these models collectively represent a large proportion of the genetic diversity of pHGGs and are syngeneically engraftable in immunocompetent mice. These GS lines produce new opportunities to evaluate subtype-specific immune and stromal involvement in pHGGs. In addition, with barcoding and pooled engraftment approaches, these GS lines can be used to investigate the relationship between genetic heterogeneity in pHGGs and their intratumoral functional specializations.

The unique propensity for H3.3^K27M^/NF1^LOF^/FGFR1^N457K^ tumors to disseminate into the thoracic spinal cord is supported by previous case reports (22, 57, 58). However, examination of spinal dissemination, and indeed exophytic spread and cranial nerve invasion, is not routinely carried out in patients. Due to this lack of systematic assessment, correlations with specific partner alterations have also not been identified. Therefore, currently it is not possible for us to confirm whether these phenomena routinely occur in the human context. Now that these models have revealed partner-specific effects, further investigation can commence in patient material if available. When confirmed in the human context, these models will provide the only tractable systems enabling experimental and therapeutic manipulation of these partner-specific invasive phenotypes.

One caveat of our approach is the inability to control the copy number of the mutations introduced with piggyBac transposition. Although transgenic approaches would be better able to address this, piggyBac is more practical for two main reasons. First, no currently available Cre driver lines are able to specifically limit transgene expression to just the LRL or GE without also implicating other progenitor types in the brain and other organs. This lack of spatiotemporal specificity is exemplified by the cerebellar and forebrain tumors often seen when using Nestin-CreERT2 to drive K27M expression and p53^LOF^ (21). Second, the number and variety of co-occurring mutations that need to be modeled to capture the genetic diversity of pHGGs would entail generating a multitude of transgenic lines, with the amount of crossing and genotyping quickly becoming excessive. PiggyBac transposition, on the other hand, allows specific and localized targeting of the LRL or GE with a range of mutations simultaneously in wild-type mice.

Besides the inability to control copy number, other limitations of IUE consist of the random nature of transposon-based genomic integration, which can disrupt endogenous gene expression and chromatin architecture. To account for this, control conditions that use the same concentration of transposon vectors and carry cargoes of similar lengths are important. In our case, these controls included the delivery of H3.3^WT^-expressing transposons together with transposons carrying the partner alterations, or delivery of the H3.3^K27M^-expressing transposon alone, at a higher total concentration mirroring the other conditions. In this way, levels of piggyBac-mediated insertional mutagenesis can be controlled for across conditions to some degree. However, despite this, the risk of integration-related genetic effects cannot be completely eliminated, and it is an important limitation to consider when using IUE-based approaches to model gliomas.

Another limitation of IUE is the highly invasive procedure that must be carried out on pregnant mice, which introduces considerations around consistency, reproducibility, and animal welfare if the requisite level of precision and skill in this surgical procedure is not adequately developed. Both reproducibility and welfare concerns can be moderated with adequate practice of the technique using cadavers prior to initiating live experiments, and in our hands, embryo viability following IUE is in excess of 95%, with similar rates of successful targeting of the correct anatomic compartment, when evaluated 96 hours following IUE, on the day of birth, and at weaning (Supplementary Fig. S2B–S2D; Supplementary Fig. S9B). However, since IUE is ultimately a manual surgical procedure that relies on electroporation with externally applied electrodes, some variability in *in vivo* transection efficiency, and the risk of gene delivery to locations that not only include but also surround the target area, cannot be eliminated completely.

For this study, we prioritized eight partner mutations for *in vivo* evaluation. A few additional alterations associated with histone mutations in pHGGs remain to be assessed. These include TOP3A in H3^K27M^ tumors and FBXW7 in H3.3^G34R^ tumors, among others. Future work will involve modeling these and other drivers of brainstem and forebrain pediatric brain tumors, such as alterations in EZHIP and FOXR2, which have been associated with hindbrain ependymomas and forebrain gliomas arising from similar progenitor niches as those targeted in this study. Cross-referencing single-cell data from these models harboring various drivers and partner alterations will help further refine and identify subtype-specific oncogenic mechanisms, stromal interactions, and therapeutic vulnerabilities.

These models are initiated by introducing mutations into a small population of progenitors in a focal and mosaic fashion in an otherwise intact system, mirroring the clonal expansion of mutant cells and the etiology of human cancer. Therefore, these models allow investigation of premalignant states and early stages of tumor development and will be important for revealing subtype-specific oncogenic mechanisms. In addition, due to the length of time needed for tumors to form, the microenvironment is slowly reprogrammed and co-opted to support disease maintenance, which recapitulates how tumors develop in patients. As our models are driven by combinations of mutations that partially mimic the mutational burden of human tumors, they recapitulate some of the genetic heterogeneity of pHGGs. Evaluating the role of each partner in isolation may not provide the same information regarding oncogenic potential, phenotypic divergence, or therapeutic susceptibility. This increased genetic complexity permits the identification of targets for precision therapy. By carrying out a small, focused screen with just eight inhibitors, we were able to identify subtype-specific susceptibility to combined PIK3CA and MEK inhibition.

Most importantly, we show that our models recapitulate key histologic and molecular features of human tumors and mirror their cell type composition at the single-cell level. These models can now be utilized to evaluate targeted therapies together with standard-of-care approaches involving conventional chemoradiotherapy to identify effective combinations. They can serve as a preclinical platform to advance translational science through the identification and validation of experimental drugs or drug targets. Furthermore, these models and GS lines will be a valuable resource for those in the pHGG research community looking to evaluate immuno-oncology or stromal interactions for therapy development, some of which (e.g., chimeric antigen receptor T-cell approaches) are beginning to show promise. Connecting preclinical evaluation of experimental treatments using these models to alterations present in patient tumors will aid in matching treatment options to stratified patient populations, helping to identify new treatment options that are more likely to succeed in the clinic.

## METHODS

### Vector Construction

The piggyBac donor and helper vector system was used to transduce NSCs *in utero* as previously described (32). CAG-PBase and PBCAG-GFP were a kind gift from F. Chen and J. LoTurco, University of Connecticut. CRISPR/Cas9 pX330 vectors containing negative control (5′-GCGACCAATACGCGAACGTC-3′), Trp53-targeting gRNA (5′ACAGCCATCACCTCACTGCA-3′), or Nf1-targeting gRNA (5′-AGTCAGCACCGAGCACAACA-3′) sequences were a kind gift from J. Gronych, German Cancer Research Center (DKFZ; ref. 59). The piggyBac vectors carrying the histone mutations and partner alterations were cloned into PBCAG-GFP using a combination of classic restriction enzyme-based cloning, gene synthesis, and the NEBuilder HiFi DNA Assembly Cloning System (New England BioLabs). All vectors drive target gene expression from the CAG promoter and short hairpin RNAs (shRNA) from either the U6 promoter or an intronic eSIBR cassette (60, 61). The mutant histone, PDGFRA, ACVR1, and Akaluc vectors expressed GFP downstream from a PQR 2A peptide, and the mutant histone was C-terminally tagged with an HA label (33). The piggyBac vectors carrying other partner alterations were tagged with an EBFP/V5 label upstream from the PQR 2A peptide. The piggyBac and CRISPR vectors used in this study were as follows: PBGAG-H3.3^WT^HA-PQR-GFP, PBCAG-H3.3^K27M^HA-PQR-GFP, PBCAG-H3.1^K27M^HA-PQR-GFP, PBCAG-H3.3^G34R^HA-PQR-GFP, PBCAG-PDGFRA^WT^-PQR-GFP, PBCAG-PDGFRA^D842V^-PQR-GFP, PBCAG-PDGFRA^C235Y^-PQR-GFP, PBCAG-ACVR1^G328V^-PQR-GFP, PBCAG-EBFP^V5^-PQR-PIK3CA^E545K^, PBCAG-EBFP^V5^-PQR-PPM1D^W420*^, PBCAG-EBFP^V5^-PQR-FGFR1^N457K^, PBCAG-EBFP^V5^-PQR-CCND2^WT^, PBU6-ATRXshRNA, PBCAG-Akaluc-PQR-GFP, CAG-PBase, PX330-Nf1gRNA-CBh-Cas9, PX330-p53gRNA-CBh-Cas9, and PX330-control gRNA-CBh-Cas9.

### IUE

IUE was performed as previously described (20), with minor modifications for targeting the hindbrain or GE. Timed-mated, pregnant C57BL/6J (RRID:IMSR_JAX:000664) mice were acquired from Charles River Laboratories and maintained under pathogen-free conditions, in individually ventilated cages, and with food and water provided *ad libitum*. All procedures were approved by the University of Cambridge Animal Welfare and Ethical Review Body and carried out under a UK Home Office License (PPL PP2303899) in accordance with the Animals (Scientific Procedures) Act 1986. Pregnant females at E12.5 (hindbrain) or E12.5 (forebrain) were anesthetized using 2.5% isoflurane and 1.5 L O_2_/minute, with analgesic support provided preoperatively via subcutaneous delivery of Buprevet at 0.1 mg/kg. Uterine horns were exposed through a 1-cm incision, and individual embryos were digitally manipulated into the correct orientation for intraventricular injection. Pulled borosilicate capillaries were loaded with endotoxin-free DNA and Fast Green dye (0.05%, Sigma) for visualization, and a microinjector (Eppendorf) was used to inject either the lateral or fourth ventricles with the DNA–dye mixture. Three to five plasmids were injected simultaneously, each up to a final concentration of 2 μg/μL, and 1 to 2 μL of total solution was injected per embryo. DNA was electroporated into GE progenitors using 5-mm tweezertrodes (BTX) or into LRL progenitors using 3-mm tweezertrodes, applying 5 square pulses at 35 V and 25 V, respectively, 50 ms each with 950 ms intervals. The embryos were returned to the abdominal cavity, the muscle and skin were sutured, and the animal was monitored until fully recovered from the procedure.

### BLI

Tumor-bearing mice received intraperitoneal administration of 100 μL of 15 mmol/L AkaLumine-HCl (HY-112641A, MedChemExpress). Approximately 5 minutes after substrate administration, the mice were anesthetized with 2.5% isoflurane and 1.5 L O_2_/minute, and bioluminescence images were acquired using an IVIS Spectrum (PerkinElmer; RRID:SCR_018621). The following conditions were used for image acquisition: open for total bioluminescence, exposure time = 60 seconds, binning = medium: 8, field of view = 13.5 × 13.5 cm, and f/stop = 1. Bioluminescent images were analyzed and exported using Living Image 4.7 software (PerkinElmer; RRID:SCR_014247).

### Perfusion Fixation

Mice were transcardially perfused under terminal anesthesia with ice-cold PBS followed by ice-cold 4% paraformaldehyde. Brains were dissected and postfixed in 4% paraformaldehyde overnight before being transferred to a gradient of 15% to 30% sucrose–PBS solutions overnight to ensure full cryoprotection of the tissue. After complete permeation with sucrose solution, brains were frozen in OCT (Sakura) on dry ice and sectioned on a Leica 3050S cryostat (Leica Biosystems; RRID:SCR_020206) into 30-μm-thick free-floating sections.

### snRNA-seq Data Preprocessing

The 10x Genomics Cell Ranger pipeline (v3.1.0) was used to trim, demultiplex, and align reads to the reference genome, distinguish cells from background, and count unique transcripts per cell. Reads were aligned to mm10 customized with added vector sequences: piggyBac transposase (with CAG promoter), piggyBac LTR-3′, Akaluc, PQR, GFP, piggyBac LTR-5′, H3.3WTHA, and P2A. Sequences for U6-Trp53gRNA-Cas9 and shATRX were also added to the reference used for KPP and KPPMPIK genotypes, whereas U6-Nf1gRNA-Cas9 was added to the reference for KNF.

Quality control of expression matrices and clustering of individual samples were performed in R (v3.6.1) with methods from the Seurat package (ref. 62; v3.2.1). Genes with expression in fewer than three cells and cells with fewer than 200 detected genes were excluded. Low-quality cells were filtered based on the following metrics: number of detected genes, number of UMIs, and percent of mitochondrial transcripts. Filtering thresholds are summarized in Supplementary Table S4.

Libraries were scaled to 10,000 UMIs per cell and log-normalized. The number of UMIs and mitochondrial content were regressed from the normalized gene counts, and the residuals were z-scored gene-wise. Dimensionality reduction was performed using principal component analysis (PCA), with 100 PCs computed using the top 2,000 most variant genes. The first 30 PCs were then used as input for visualization in two dimensions using uniform manifold approximation and projection (UMAP; arXiv 1802.03426v3) and as input for clustering. Cells were clustered using a shared nearest neighbor (SNN) modularity optimization algorithm (63) using the Louvain algorithm on a k-nearest neighbor graph with *k* = 20 and the resolution parameter set to 1. Cell-cycle scores for G_2_–M and S phases were computed as implemented in Seurat using G_2_–M and S phase–associated signatures converted to murine homologs (64). Scores were obtained by calculating the average expression of phase-associated genes and subtracting the average expression of control gene lists. Control gene lists were derived by binning genes in each input list into 24 bins according to expression levels and randomly selecting 100 control genes from within each expression bin. Postclustering quality control was performed to remove clusters with low sequencing depth and high mitochondrial content.

### Integration of Samples within and across Genotypes

To identify cell populations shared by KNF and KPP samples, samples were integrated using Harmony (ref. 65; v1.0). Due to imbalanced cell numbers between genotypes, each KNF replicate was first downsampled to match the KPP replicates (*N* = 15,537), such that proportions of clusters identified in individual sample analysis were maintained. The number of UMIs and mitochondrial content were regressed from the merged normalized gene counts, and PCA of variant genes was performed as described above. The first 30 PCs were aligned by sample using the following parameters: theta = 2, lambda = 1, sigma = 0.1. The “harmonized” PCs were used as input for visualization with UMAP and for clustering using an SNN modularity optimization algorithm using the Louvain algorithm on a k-nearest neighbor graph with *k* = 20 and resolution = 0.5. Integration of the KPPMPIK replicates was performed with the same workflow.

### Consensus Cell Type Identification

To identify cell types in the snRNA-seq data, we implemented an automated, reference-based annotation workflow based on four prediction methods: SciBet (66), SingleCellNet (67), SingleR (68), and Spearman correlation. A consensus annotation was assigned when at least two methods agreed without a tie. Cell type annotation was performed using two murine brain cell type references. Immune cells were first labeled at the cluster level in individual samples based on expression of canonical markers (Ptprc, Ly86, Hexb, Cd84, Ccr5, Cd247, Skap1, Zfp366, and Btla). These clusters were annotated with a single-cell dataset of normal mouse brain CD45^+^ immune cells as the training reference (50). Nonimmune cells were then annotated with a single-cell dataset of embryonic and early postnatal murine brain (27), with the consensus labels defined without age information. The consensus labels were summarized as broad cell classes: radial glial cells, OPCs, glial progenitors, oligodendrocytes, astrocytes, ependymal cells, neuronal progenitors, neurons, vascular cells, immune cells, and other. This ontology is detailed in Supplementary Table S5. Only cells with a consensus prediction from agreement of at least two methods were used for downstream analysis.

### Gene Set Enrichment of Human Tumor Signatures

To assess the expression of human tumor-derived signatures in the murine models, we performed ssGSEA with code adapted from the GSVA package (v.1.27.0) as described (27). Enrichment in single cells was scored for four signatures derived from previous studies of bulk RNA-seq and differential expression analysis between several tumor entities (13, 27). This includes signatures from comparing H3.3^K27M^ and H3.1^K27M^ pontine high-grade glioma (*N* = 20; refs. 4, 27, 69, 70), H3.3^K27M^ high-grade glioma and EZHIP PFA (*N* = 28; refs. 4, 27, 69–71), and G34R/V and H3.3^K27M^ high-grade glioma (*N* = 26; refs. 13, 27). All differential expression analyses yielding the signatures were performed using DESeq2 (v1.14.1). Signatures were processed by removing mitochondrial genes (defined as beginning with “MT-”), selecting the top 100 genes based on logFC/variance(logFC), and converting to mouse homologs. The top 200 genes based on logFC/variance(logFC) were selected for the H3.1^K27M^ signature to obtain a number of mouse homologs comparable to the other signatures. The signatures used for ssGSEA scoring are found in Supplementary Table S6.

### TF Activity Inference

The activity of TFs and their regulated genes were inferred from normalized expression data with the python implementation of SCENIC (ref. 51; v.0.10.0) through the Singularity (v.3.8) container. Briefly, modules of genes coexpressed with TFs were detected through reconstructing a gene regulatory network. TFs with binding motifs enriched in their corresponding modules were retained, and genes containing the binding motifs as potential direct targets were retained in the modules. The AUCell algorithm (51) was then used to compute an activity score for each TF's module in each cell. The above workflow was performed with the merged data across replicates for KNF and KPP genotypes separately.

### *Ex Vivo* NSC/GS Isolation and Culture

Tumor-bearing C57BL/6J mice were euthanized by CO_2_ exposure. Brains were rapidly dissected in ice-cold dissociation medium (DM) containing 20 mmol/L glucose, 81.8 mmol/L Na_2_SO_4_, 30 mmol/L K_2_SO_4_, 5.8 mmol/L MgCl_2_, 250 μmol/L CaCl_2_, 1 mmol/L HEPES, 160 μmol/L NaOH, 0.8 mmol/L kynurenic acid, 50 μmol/L D-APV, 100 U/mL penicillin, 100 μg/mL streptomycin, 5 μg/mL plasmocin, and 100 μg/mL primocin. Coronal sections were cut using a brain matrix and GFP^+^ (tumor) and GFP^−^ (stroma) regions were microdissected under an epifluorescence stereomicroscope (Leica M205, Leica Biosystems). Microdissected tissue was then enzymatically digested into a single-cell suspension using the Papain Dissociation System according to the manufacturer's instructions (Worthington Biochemicals). The dissociated cell solution was separated on an OptiPrep density gradient to remove debris, following which GFP^+^ cells were sorted using a FACSAria II (BD Biosciences). Sorted cells were plated into NeuroCult NSC proliferation media (STEMCELL Technologies) containing 20 ng/mL EGF (Miltenyi Biotec), 20 ng/mL bFGF (Miltenyi Biotec), 10 ng/mL PDGF-AA (Shenandoah Biotechnology), 10 ng/mL PDGF-BB (Shenandoah Biotechnology), and 2 μg/mL heparin (STEMCELL Technologies). Cells were grown as spheroids (GS) using ultra-low attachment plates (Corning). Cell lines were authenticated using short tandem repeat profiling and contamination with *Mycoplasma* was tested using the qPCR-based PhoenixDX Mycoplasma Detection Kit. The results were recorded with the cell line authentication service at the CRUK Cambridge Institute. Cell lines at or below passage number 15 were used in experiments.

### Patient-Derived Cell Culture

Patient-derived cell lines were provided by N. Jabado. BT416 (RRID:CVCL_WW64), BT869 (RRID:CVCL_C1MH), SU-DIPG-VI (RRID:CVCL_IT40), G477, and GBM002 cells were grown at 37°C and 5% CO_2_ in stem cell media consisting of Neurocult NS-A Basal Medium (human, STEMCELL Technologies) and Neurocult NS-A Proliferation Supplement (human, STEMCELL Technologies). The media were supplemented with 20 ng/mL EGF (STEMCELL Technologies), 10 ng/mL bFGF (STEMCELL Technologies), and 0.2% heparin (Stem Cell Technologies). Cells were cultured in flasks precoated with 0.01% poly-L-ornithine and 1 mg/mL laminin diluted in PBS.

### Drug-Sensitivity Assays

Mouse tumor-derived GS cells and patient-derived cell lines were plated in 96-well plates and treated with eight compounds at different concentrations, either for 3 days (avapritinib, FK866, idasanutlin, and trametinib) or for 6 days (alpelisib, corin, GSK-J4, and infigratinib). At the endpoint, cell viability was measured using CellTiter-Glo (2.0 Promega). Relative luminescence units (RLU) for each well were normalized to the median RLU from the DMSO control wells as 100% viability. Three technical replicates per drug condition were performed as well as two independent biological replicates. IC_50_ values (drug concentration causing 50% inhibition of cell proliferation) were calculated using GraphPad Prism (RRID:SCR_002798), and the curves show the mean ± SD of the replicates per condition measured. Statistics are summarized in Supplementary Table S7. All compounds were purchased from MedChemExpress and were diluted in DMSO to a final concentration of 10 mmol/L.

### RNA Extraction and qPCR

RNA was extracted using the Rneasy Plus Kit (Qiagen) according to the manufacturer's instructions. RNA quality and quantification were carried out using a Nanodrop spectrophotometer (Thermo Fisher) or a Bioanalyzer (Agilent). Total RNA (500 ng) was used for reverse transcription using TaqMan Reverse Transcription Reagents (Thermo Fisher), and real-time PCR was performed using the SsoAdvanced Universal SYBR Green Supermix (Bio-Rad) on a CFX96 Touch Real-Time PCR Detection System (Bio-Rad; RRID:SCR_018064). Primers are described in Supplementary Table S8. The 2-^ΔΔCT^ method was used to calculate relative gene expression levels, and gene expression was normalized to mouse B2M levels.

### Western Blotting

Total protein extraction was performed using lysis buffer (Tris-HCl 50 mmol/L pH 8, NaCl 150 mmol/L, Triton-X-100 1%, NaF 100 mmol/L, EDTA 1 mmol/L, MgCl_2_ 1 mmol/L, and glycerol 10%) containing a protease inhibitor cocktail (Sigma-Aldrich). Equal amounts of total protein were subjected to Bis-Tris gel electrophoresis (Nupage) and then transferred to nitrocellulose membranes using the iBlot2 Dry Blotting System (Thermo Fisher). The membranes were blocked with Intercept PBS Blocking Buffer (Li-Cor) and incubated with primary antibodies overnight at 4°C (Supplementary Table S9). After washing, membranes were incubated with Li-Cor Fluorescent IRDye secondary antibodies at room temperature (800 CW and 680 CW dyes) for 1 hour. Detection was performed with the Li-Cor Odyssey FC Imaging System. Histone extraction was performed using the Histone Extraction Kit (Active Motif). Cells were lysed according to the one-step extraction protocol provided by the manufacturer, and histone lysates then underwent Bis-Tris gel electrophoresis, transfer, and detection as described above.

### Histology

For histologic analysis of tumors, paraformaldehyde-fixed brains were embedded in paraffin, sectioned at a thickness of 3 μm, and processed for H&E staining. Immunohistologic detection of various antigens was performed on the Leica automated Bond-III platform in conjunction with the Polymer Refine Detection System (DS9800) using biotin-free secondary antibodies coupled to streptavidin–horseradish peroxidase and diaminobenzidine as a chromogen (Supplementary Table S9). Slides were then mounted using DPX Mountant for Histology (#06522, Sigma-Aldrich), digitized on a Leica Aperio AT2, and viewed and exported using the Aperio ImageScope software (Leica Biosystems).

### Immunofluorescence *In Vivo*

For immunofluorescence, free-floating sections were incubated in a blocking solution (10% goat or donkey serum, 3% BSA, 0.3% Triton-X in PBS) for 1 hour at room temperature and then incubated with primary antibodies at 4°C overnight (Supplementary Table S9). Sections were washed in PBS-Tween (0.05%) before the addition of Hoechst 33342 and Alexa Fluor–conjugated secondary antibodies in a blocking solution for 1 hour at room temperature. Following washing, sections were mounted in ProLong Diamond Antifade mountant (Thermo Fisher Scientific) and imaged on a confocal microscope (Leica SP8, Leica Biosystems).

### Quantification of H3K27me3 and Ki-67 in Tissue Sections

H3K27me3 fluorescence signal in HA^+^ cells was quantified using both ImageJ (RRID:SCR_003070) and Metamorph software (Molecular Devices; RRID:SCR_002368). Two to four single-plane confocal images with a z-step of 2 μm were combined for fluorescence intensity analysis (total depth less than 10 μm). GFP^+^Ki-67^+^ cells in the striatum and pons were quantified using ImageJ and QuPath (RRID:SCR_018257). Briefly, a DAPI mask was generated and used to quantify the total number of GFP^+^ tumor cells, and the total number of GFP^+^Ki-67^+^ cells was then counted and used to calculate the mitotic index of the tumor. All cell counting and fluorescent intensity analyses were carried out on images from a minimum of 3 mice per condition.

### Orthotopic Allotransplantation

Eight-week-old C57BL/6J mice (Charles River Laboratories) were maintained under pathogen-free conditions, in individually ventilated cages, and with food and water provided *ad libitum*. All procedures were approved by the UK Home Office (PPL PP2303899) and carried out in accordance with the Animals (Scientific Procedures) Act 1986. Analgesia was provided preoperatively via a subcutaneous injection of Buprevet (0.1 mg/kg). Mice were anesthetized (2.5% isoflurane and 1.5 L O_2_/minute), and the head of the mouse was fixed in a stereotactic frame (#51730, Stoelting Europe). A midline incision was made along the scalp to expose the skull and a small burr hole was made using a high-speed drill at defined stereotaxic coordinates: 0.5 mm anterior and 1.8 mm lateral from bregma for striatal targeting, and 0.8 mm posterior and 1.1 mm lateral from lambda for pontine targeting. A total of 1.5 × 10^5^ to 3 × 10^5^ GS cells resuspended in 5 μL of PBS were then delivered at a depth of 3.2 mm or 5 mm, respectively, using a 26-gauge (2 mm, AS point style) Hamilton Syringe at a controlled rate of 2 μL/minute before the needle was removed at a rate of 0.5 mm/minute. The scalp was then closed with sutures, and mice were placed in a heat chamber until fully recovered before being returned to their home cage.

### *In Vivo* Drug Treatment

Animals were intracranially engrafted in either striatum or pons with GS cells and received either oral administration 4 days after engraftment or were implanted with an ALZET mini–osmotic pump (Durect) 7 days after engraftment. Mice were randomized based on bioluminescence signal prior to treatment initiation. For oral gavage treatment, avapritinib was dissolved in 10% dimethyl sulfoxide (DMSO) and 90% saline (containing 20% SBE-β-CD saline), and mice received either avapritinib (30 mg/kg) or vehicle for 15 consecutive days via this route. For osmotic pump treatment, a 14-day ALZET pump (Model 2002) was connected to an ALZET brain infusion cannula (ALZET brain infusion kit 2), filled, and primed with the treatment or vehicle (elacridar in 20% SBE-β-CD saline) as per the manufacturer's instructions prior to implantation. The pump was inserted into a subcutaneous pocket slightly posterior to the scapulae, and the cannula was installed in the fourth ventricle. The cannula pedestal was secured in place using cyanoacrylate gel (Loctite 454), and pumps were removed following treatment as per the manufacturer's recommendation.

### Data Availability

The data generated in this study are available within the article and its supplementary data files.

## Supplementary Material

Supplementary Tables 1-3, 7-9 Tables describing statistical analyses, median survivals, IC50 values calculated from cell viability assays, primer sequences and antibodies.

Supplementary Table 4 Quality control and filtering of snRNA-seq data.

Supplementary Table 5 Cell type ontologies derived from snRNA-seq data.

Supplementary Table 6 ssGEA signatures derived from snRNA-seq data.

Supplementary Figures 1-27 Figures describing hindbrain vs ganglionic eminence targeting strategies, each of the 16 models generated and validation that they expressed the introduced mutations, survival of GE vs CTX-electroporated embryos, and cell viability data shown in bar graph format.
