# Correction: A Compendium of Syngeneic, Transplantable Pediatric High-Grade Glioma Models Reveals Subtype-Specific Therapeutic Vulnerabilities

**DOI:** 10.1158/2159-8290.CD-24-0299

**Published:** 2024-05-01

In the original version of this article (1), the label above the top set of images in Fig. 1C was mislabeled as “GPAD” by the compositor during production of the paper and should instead read “GPAP.” Figure 1C has been corrected in the latest online HTML and PDF versions of the article. The publisher regrets the error.
