# Supplementary material for: A Compendium of Syngeneic, Transplantable Pediatric High-Grade Glioma Models Reveals Subtype-Specific Therapeutic Vulnerabilities
Source: Cancer Discov. 2023 Apr 3;13(7):1592–615. doi: 10.1158/2159-8290.CD-23-0004 (PMC10326601; doi:10.1158/2159-8290.CD-23-0004)
Supplement: Supplementary Tables 1-3, 7-9 — Tables describing statistical analyses, median survivals, IC50 values calculated from cell viability assays, primer sequences and antibodies. [file cd-23-0004_supplementary_tables_1-3_7-9_suppst1.pdf]

Supplementary Table 1. Nomenclature key for the 16 pHGG subtype models.

Mutation combinations	Acronym
H3.3^{G34R}, p53^{LOF}, ATRX^{LOF} PDGFRA^{WT}	GPAP
H3.3^{G34R}, p53^{LOF}, ATRX^{LOF} PDGFRA^{C235Y}	GPAC
H3.3^{G34R} , p53^{LOF}, ATRX^{LOF} PDGFRA^{D842V}	GPAD
H3.3^{K27M}, p53^{LOF}	KP
H3.3^{K27M}, p53^{LOF}, PDGFRA^{WT}	KPP
H3.3^{K27M}, p53^{LOF}, PDGFRA^{D842V}	KPD
H3.3^{K27M}, p53^{LOF}, ATRX^{LOF}, PDGFRA^{WT}	KPAP
H3.3^{K27M}, p53^{LOF}, ATRX^{LOF}, PDGFRA^{D842V}	KPAD
H3.3^{K27M}, FGFR1^{N457K}	KF
H3.3^{K27M}, p53^{LOF}, FGFR1^{N457K}	KPF
H3.3^{K27M}, NF1^{LOF}	KN
H3.3^{K27M}, NF1^{LOF}, FGFR1^{N457K}	KNF
H3.3^{K27M}, PPM1D$^{\Delta C}$, PIK3CA^{E545K}	KPPMPIK
H3.1^{K27M}, p53^{LOF}	H3.1KP
H3.1^{K27M}, ACVR1^{G328V}, PIK3CA^{E545K}	H3.1KACVPIK
H3.3^{K27M}, p53^{LOF}, CCND2	KPC

Supplementary Table 2. Statistical analyses of Kaplan-Meier survival data shown in main figures (**Fig. 1, 2, 4, and 6**).

Significance levels and *p* values corresponding to Kaplan-Meier data as determined by the logrank (Mantel-Cox) test

**Comparison**	**Summary**	**P**
**Figure 1E**		
H3.3^{G34R}, p53^{LOF}, ATRX^{LOF} **vs** H3.3^{G34R}, p53^{LOF}, ATRX^{LOF}, PDGFRA^{WT}	ns	P = 0.0882
H3.3^{G34R}, p53^{LOF}, ATRX^{LOF} **vs** H3.3^{G34R}, p53^{LOF}, ATRX^{LOF}, PDGFRA^{C235Y}	ns	P = 0.1459
H3.3^{G34R}, p53^{LOF}, ATRX^{LOF} **vs** H3.3^{G34R}, p53^{LOF}, ATRX^{LOF}, PDGFRA^{D842V}	**	P = 0.0029
H3.3^{G34R}, p53^{LOF}, ATRX^{LOF}, PDGFRA^{WT} **vs** H3.3^{G34R}, p53^{LOF}, ATRX^{LOF}, PDGFRA^{C235Y}	ns	P = 0.7474
H3.3^{G34R}, p53^{LOF}, ATRX^{LOF}, PDGFRA^{WT} **vs** H3.3^{G34R}, p53^{LOF}, ATRX^{LOF}, PDGFRA^{D842V}	**	P = 0.0029
H3.3^{G34R}, p53^{LOF}, ATRX^{LOF}, PDGFRA^{D842V} **vs** H3.3^{G34R}, p53^{LOF}, ATRX^{LOF}, PDGFRA^{C235Y}	*	P = 0.0133
**Figure 2D**		
H3.3^{K27M} **vs** H3.3^{K27M}, p53^{LOF}, PDGFRA^{WT}	***	P = 0.0002
H3.3^{K27M} **vs** H3.3^{K27M}, p53^{LOF}, PDGFRA^{D842V}	****	P = <0.0001
H3.3^{K27M}, p53^{LOF}, PDGFRA^{WT} **vs** H3.3^{K27M}, p53^{LOF}, PDGFRA^{D842V}	***	P = 0.0002
H3.1^{K27M} **vs** H3.1^{K27M}, ACVR1^{G328V}, PIK3CA^{E545K}	**	P = 0.0011
H3.3^{K27M}, PPM1D$^{\Delta C}$, PIK3CA^{E545K} **vs** H3.3^{K27M}, PPM1D$^{\Delta C}$	*	P = 0.0428
**Figure 4C**		
H3.3^{K27M} **vs** H3.3^{K27M}, p53^{LOF}	***	P = 0.0002
H3.3^{K27M} **vs** H3.3^{K27M}, p53^{LOF}, PDGFRA^{WT}	***	P = 0.0002
H3.3^{K27M} **vs** H3.3^{K27M}, p53^{LOF}, PDGFRA^{D842V}	****	P = <0.0001
H3.3^{K27M}, p53^{LOF} **vs** H3.3^{K27M}, p53^{LOF}, PDGFRA^{WT}	ns	P = 0.2460
H3.3^{K27M}, p53^{LOF} **vs** H3.3^{K27M}, p53^{LOF}, PDGFRA^{D842V}	****	P = <0.0001
H3.1^{K27M} **vs** H3.1^{K27M}, p53^{LOF}	**	P = 0.0038
H3.1^{K27M} **vs** H3.1^{K27M}, ACVR1^{G328V}, PIK3CA^{E545K}	**	P = 0.0011
H3.1^{K27M}, p53^{LOF} **vs** H3.1^{K27M}, ACVR1^{G328V}, PIK3CA^{E545K}	ns	P = 0.1820
H3.3^{K27M}, p53^{LOF}, FGFR1^{N457K} **vs** H3.3^{K27M}, FGFR1^{N457K}	***	P = 0.0004
H3.3^{K27M}, p53^{LOF}, FGFR1^{N457K} **vs** H3.3^{K27M}, NF1^{LOF}, FGFR1^{N457K}	****	P = <0.0001
H3.3^{K27M}, p53^{LOF}, FGFR1^{N457K} **vs** H3.3^{K27M}, NF1^{LOF}	**	P = 0.0033
H3.3^{K27M}, FGFR1^{N457K} **vs** H3.3^{K27M}, NF1^{LOF}, FGFR1^{N457K}	****	P = <0.0001
H3.3^{K27M}, FGFR1^{N457K} **vs** H3.3^{K27M}, NF1^{LOF}	ns	P = 0.0737
H3.3^{K27M}, NF1^{LOF}, FGFR1^{N457K} **vs** H3.3^{K27M}, NF1^{LOF}	***	P = 0.0003
H3.3^{WT}, p53^{LOF}, PDGFRA^{D842V} **vs** H3.3^{K27M}, p53^{LOF}, PDGFRA^{D842V}	ns	P = 0.2035
**Figure 6D (orthotopic allografts)**		
H3.3^{K27M}, p53^{LOF} **vs** H3.3^{K27M}, p53^{LOF}, PDGFRA^{WT}	**	P = 0.0016
H3.3^{K27M}, p53^{LOF} **vs** H3.3^{K27M}, p53^{LOF}, FGFR1^{N457K}	**	P = 0.0018

H3.3^{K27M}, p53^{LOF} **vs** H3.1^{K27M}, p53^{LOF}	**	P = 0.0018
H3.3^{K27M}, p53^{LOF} **vs** H3.3^{K27M}, PPM1D$^{\Delta C}$, PIK3CA^{E545K}	**	P = 0.0018
H3.3^{K27M}, p53^{LOF} **vs** H3.3^{G34R}, p53^{LOF}, ATRX^{LOF}, PDGFRA^{D842V}	**	P = 0.0018
H3.3^{K27M}, p53^{LOF} **vs** H3.3^{G34R}, p53^{LOF}, ATRX^{LOF}, PDGFRA^{C235Y}	**	P = 0.0090
H3.3^{K27M}, p53^{LOF}, PDGFRA^{WT} **vs** H3.3^{K27M}, p53^{LOF}, FGFR1^{N457K}	**	P = 0.0016
H3.3^{K27M}, p53^{LOF}, PDGFRA^{WT} **vs** H3.1^{K27M}, p53^{LOF}	**	P = 0.0016
H3.3^{K27M}, p53^{LOF}, PDGFRA^{WT} **vs** H3.3^{K27M}, PPM1D$^{\Delta C}$, PIK3CA^{E545K}	**	P = 0.0016
H3.3^{K27M}, p53^{LOF}, PDGFRA^{WT} **vs** H3.3^{G34R}, p53^{LOF}, ATRX^{LOF}, PDGFRA^{D842V}	**	P = 0.0016
H3.3^{K27M}, p53^{LOF}, PDGFRA^{WT} **vs** H3.3^{G34R}, p53^{LOF}, ATRX^{LOF}, PDGFRA^{C235Y}	**	P = 0.0016
H3.3^{K27M}, p53^{LOF}, FGFR1^{N457K} **vs** H3.1^{K27M}, p53^{LOF}	**	P = 0.0018
H3.3^{K27M}, p53^{LOF}, FGFR1^{N457K} **vs** H3.3^{K27M}, PPM1D$^{\Delta C}$, PIK3CA^{E545K}	**	P = 0.0044
H3.3^{K27M}, p53^{LOF}, FGFR1^{N457K} **vs** H3.3^{G34R}, p53^{LOF}, ATRX^{LOF}, PDGFRA^{D842V}	ns	P = 0.2426
H3.3^{K27M}, p53^{LOF}, FGFR1^{N457K} **vs** H3.3^{G34R}, p53^{LOF}, ATRX^{LOF}, PDGFRA^{C235Y}	ns	P = 0.3984
H3.1^{K27M}, p53^{LOF} **vs** H3.3^{K27M}, PPM1D$^{\Delta C}$, PIK3CA^{E545K}	ns	P = 0.0752
H3.1^{K27M}, p53^{LOF} **vs** H3.3^{G34R}, p53^{LOF}, ATRX^{LOF}, PDGFRA^{D842V}	**	P = 0.0018
H3.1^{K27M}, p53^{LOF} **vs** H3.3^{G34R}, p53^{LOF}, ATRX^{LOF}, PDGFRA^{C235Y}	ns	P = 0.6101
H3.3^{K27M}, PPM1D$^{\Delta C}$, PIK3CA^{E545K} **vs** H3.3^{G34R}, p53^{LOF}, ATRX^{LOF}, PDGFRA^{D842V}	*	P = 0.0127
H3.3^{K27M}, PPM1D$^{\Delta C}$, PIK3CA^{E545K} **vs** H3.3^{G34R}, p53^{LOF}, ATRX^{LOF}, PDGFRA^{C235Y}	ns	P = 0.6702
H3.3^{G34R}, p53^{LOF}, ATRX^{LOF}, PDGFRA^{D842V} **vs** H3.3^{G34R}, p53^{LOF}, ATRX^{LOF}, PDGFRA^{C235Y}	ns	P = 0.5783
**Figure 7B (avapritinib treated orthotopic allografts)**		
H3.3^{K27M}, p53^{LOF}, PDGFRA^{WT} vehicle **vs** H3.3^{K27M}, p53^{LOF}, PDGFRA^{WT} avapritinib	**	P = 0.0023

Supplementary Table 3. Median survivals from Kaplan-Meier survival curves shown in main figures (**Fig. 1, 2, 4, and 6**).

**Median survivals of *de novo* models (generated using IUE)**			
**Experimental Condition**	**Location**	**Median Survival (Days)**	**n**
H3.3^{G34R}, p53^{LOF}, ATRX^{LOF}	Ganglionic Eminence	Euthanized at 2 years	3
H3.3^{G34R}, p53^{LOF}, ATRX^{LOF}, PDGFRA^{WT}	Ganglionic Eminence	453	15
H3.3^{G34R}, p53^{LOF}, ATRX^{LOF}, PDGFRA^{C235Y}	Ganglionic Eminence	296	17
H3.3^{G34R}, p53^{LOF}, ATRX^{LOF}, PDGFRA^{D842V}	Ganglionic Eminence	67	7
H3.3^{WT}, p53^{LOF}, ATRX^{LOF}, PDGFRA^{WT}	Ganglionic Eminence	76	5
H3.3^{WT}, p53^{LOF}, ATRX^{LOF}, PDGFRA^{C235Y}	Ganglionic Eminence	Euthanized at 2 years	7
H3.3^{WT}, p53^{LOF}, ATRX^{LOF}, PDGFRA^{D842V}	Ganglionic Eminence	96	5
H3.3^{K27M}	Hindbrain	Euthanized at 2 years	5
H3.3^{K27M}, p53^{LOF}	Hindbrain	275	6
H3.3^{K27M}, p53^{LOF}, PDGFRA^{WT}	Hindbrain	230	6
H3.3^{K27M}, p53^{LOF}, PDGFRA^{D842V}	Hindbrain	34.5	12
H3.3^{K27M}, p53^{LOF}, ATRX^{LOF}, PDGFRA^{WT}	Hindbrain	207	9
H3.3^{K27M}, p53^{LOF}, ATRX^{LOF}, PDGFRA^{D842V}	Hindbrain	45	7
H3.3^{WT}, p53^{LOF}, PDGFRA^{D842V}	Hindbrain	37	6
H3.3^{WT}, p53^{LOF}	Hindbrain	Euthanized at 2 years	5
H3.3^{K27M}, FGFR1^{N457K}	Hindbrain	209.5	12
H3.3^{K27M}, p53^{LOF}, FGFR1^{N457K}	Hindbrain	47	16
H3.3^{K27M}, NF1^{LOF}	Hindbrain	199	7
H3.3^{K27M}, NF1^{LOF}, FGFR1^{N457K}	Hindbrain	28	17
H3.3^{WT}, NF1^{LOF}	Hindbrain	Euthanized at 2 years	5
H3.3^{WT}, FGFR1^{N457K}	Hindbrain	Euthanized at 2 years	4
H3.3^{WT}, p53^{LOF}, FGFR1^{N457K}	Hindbrain	103	5
H3.3^{K27M}, PPM1D$^{\Delta C}$	Hindbrain	422.5 (only 1 of 4 tumor-related)	4
H3.3^{K27M}, PPM1D$^{\Delta C}$, PIK3CA^{E545K}	Hindbrain	42	22
H3.3^{WT}, PPM1D$^{\Delta C}$, PIK3CA^{E545K}	Hindbrain	Euthanized at 2 years	5
H3.1^{K27M}	Hindbrain	Euthanized at 2 years	4
H3.1^{K27M}, p53^{LOF}	Hindbrain	153	6
H3.1^{K27M}, ACVR1^{G328V}	Hindbrain	Euthanized at 2 years	4
H3.1^{K27M}, ACVR1^{G328V}, PIK3CA^{E545K}	Hindbrain	78	13

$H3.3^{K27M}$, $p53^{LOF}$, CCND2	Hindbrain	217	9
**Median survivals of orthotopic allografts**			
**Experimental Condition**	**Location**	**Median Survival (Days)**	**n**
$H3.3^{K27M}$, $p53^{LOF}$	Hindbrain	24	5
$H3.3^{K27M}$, $NF1^{LOF}$	Hindbrain	53	5
$H3.3^{K27M}$, $FGFR1^{N457K}$	Hindbrain	40	5
$H3.3^{K27M}$, $p53^{LOF}$, $PDGFRA^{WT}$	Hindbrain	19	5
$H3.3^{K27M}$, $p53^{LOF}$, $FGFR1^{N457K}$	Hindbrain	37	5
$H3.3^{K27M}$, $PPM1D^{\Delta C}$, $PIK3CA^{E545K}$	Hindbrain	79	5
$H3.1^{K27M}$, $p53^{LOF}$	Hindbrain	105	5
$H3.1^{K27M}$, $ACVR1^{G328V}$, $PIK3CA^{E545K}$	Hindbrain	116	5
$H3.3^{G34R}$, $p53^{LOF}$, $ATRX^{LOF}$, $PDGFRA^{WT}$	Striatum	Undefined (<50% penetrant)	5
$H3.3^{G34R}$, $p53^{LOF}$, $ATRX^{LOF}$, $PDGFRA^{D842V}$	Striatum	40	5
$H3.3^{G34R}$, $p53^{LOF}$, $ATRX^{LOF}$, $PDGFRA^{C235Y}$	Striatum	36	5
**Median survivals of avapritinib treatment in orthotopic allografts**			
**Experimental Condition**	**Location**	**Median Survival (Days)**	**n**
$H3.3^{K27M}$, $p53^{LOF}$, $PDGFRA^{WT}$ vehicle	Hindbrain	18	5
$H3.3^{K27M}$, $p53^{LOF}$, $PDGFRA^{WT}$ avapritinib	Hindbrain	26	5

Supplementary Table 7. Statistical analyses for dose-response curves shown in main figure (**Fig. 1 and 7**).

Drug	Cell line	IC50 (μm)	95% CI
Alpelisib	H3.3^{G34R}, p53^{LOF}, ATRX^{LOF}, PDGFRA^{WT}	2.196	1.965 to 2.460
	H3.3^{G34R}, p53^{LOF}, ATRX^{LOF}, PDGFRA^{C235Y}	0.5352	0.4777 to 0.5982
	H3.3^{G34R}, p53^{LOF}, ATRX^{LOF}, PDGFRA^{D842V}	6.427	4.929 to 8.707
	H3.3^{K27M}, p53^{LOF}	0.2291	0.1820 to 0.2819
	H3.3^{K27M}, p53^{LOF}, PDGFRA^{WT}	0.8263	0.7065 to 0.9650
	H3.3^{K27M}, p53^{LOF}, PDGFRA^{D842V}	3.554	2.147 to 6.420
	H3.3^{K27M}, FGFR1^{N457K}	1.349	1.071 to 1.705
	H3.3^{K27M}, p53^{LOF}, FGFR1^{N457K}	$+ \infty$	NA
	H3.3^{K27M}, NF1^{LOF}	2.752	2.225 to 3.442
	H3.3^{K27M}, NF1^{LOF}, FGFR1^{N457K}	0.1610	0.1165 to 0.2114
	H3.3^{K27M}, PPM1D$^{\Delta C}$, PIK3CA^{E545K}	1.049	0.7269 to 1.503
	H3.1^{K27M}, p53^{LOF}	3.868	2.368 to 6.956
	H3.1^{K27M}, PIK3CA^{E545K}, ACVR1^{G328V}	1.219	0.7106 to 2.106
	G477 (H3.3^{WT}) - human	1.631	1.106 to 2.413
	SU-DIPG-VI (H3.3^{K27M}, p53$^{R34H/E66X}$, PDGFRA^{WT}) – human	1.060	0.6300 to 1.812
	BT-869 (H3.3^{K27M}, PPM1D$^{\Delta C}$, PIK3CA^{E545K}, ACVR1^{R206H}, p53^{R272H}) – human	0.4217	0.2150 to 0.8326
	BT-416 (H3.3^{K27M}, p53^{R116Q}, NF1$^{L1607X/R2616X}$) – human	NA	NA
Avapritinib	H3.3^{G34R}, p53^{LOF}, ATRX^{LOF}, PDGFRA^{WT}	1.653	1.175 to 2.369
	H3.3^{G34R}, p53^{LOF}, ATRX^{LOF}, PDGFRA^{C235Y}	1.462	1.170 to 1.837
	H3.3^{G34R}, p53^{LOF}, ATRX^{LOF}, PDGFRA^{D842V}	1.781	1.275 to 2.536
	H3.3^{K27M}, p53^{LOF}	0.1504	0.09405 to 0.2375
	H3.3^{K27M}, p53^{LOF}, PDGFRA^{WT}	0.03175	0.01761 to 0.04935
	H3.3^{K27M}, p53^{LOF}, PDGFRA^{D842V}	0.3444	0.2698 to 0.4373
	H3.3^{K27M}, FGFR1^{N457K}	0.7322	0.5780 to 0.9291
	H3.3^{K27M}, p53^{LOF}, FGFR1^{N457K}	0.6401	0.4693 to 0.8757
	H3.3^{K27M}, NF1^{LOF}	0.4383	0.3228 to 0.5915
	H3.3^{K27M}, NF1^{LOF}, FGFR1^{N457K}	0.5302	0.3504 to 0.8029
	H3.3^{K27M}, PPM1D$^{\Delta C}$, PIK3CA^{E545K}	0.2213	0.1370 to 0.3525
	H3.1^{K27M}, p53^{LOF}	0.1497	0.1380 to 0.1624
	H3.1^{K27M}, PIK3CA^{E545K}, ACVR1^{G328V}	0.9602	0.4773 to 1.979
Corin	H3.3^{G34R}, p53^{LOF}, ATRX^{LOF}, PDGFRA^{WT}	0.7641	0.5074 to 1.179
	H3.3^{G34R}, p53^{LOF}, ATRX^{LOF}, PDGFRA^{C235Y}	0.1216	0.1033 to 0.1417
	H3.3^{G34R}, p53^{LOF}, ATRX^{LOF}, PDGFRA^{D842V}	0.7989	0.5409 to 1.209
	H3.3^{K27M}, p53^{LOF}	2.815	1.176 to 26.15
	H3.3^{K27M}, p53^{LOF}, PDGFRA^{WT}	1.227	0.4666 to 5.259
	H3.3^{K27M}, p53^{LOF}, PDGFRA^{D842V}	0.1485	0.09938 to 0.2113
	H3.3^{K27M}, FGFR1^{N457K}	0.2493	0.1592 to 0.3767
	H3.3^{K27M}, p53^{LOF}, FGFR1^{N457K}	1.260	0.7813 to 2.205
	H3.3^{K27M}, NF1^{LOF}	0.4732	0.2112 to 1.023
	H3.3^{K27M}, NF1^{LOF}, FGFR1^{N457K}	0.1309	0.1148 to 0.1483
	H3.3^{K27M}, PPM1D$^{\Delta C}$, PIK3CA^{E545K}	0.2493	0.1546 to 0.3860
	H3.1^{K27M}, p53^{LOF}	0.3893	0.2178 to 0.6813
	H3.1^{K27M}, PIK3CA^{E545K}, ACVR1^{G328V}	0.1259	0.1049 to 0.1493
FK866	H3.3^{G34R}, p53^{LOF}, ATRX^{LOF}, PDGFRA^{WT}	2.427	1.506 to 4.254
	H3.3^{G34R}, p53^{LOF}, ATRX^{LOF}, PDGFRA^{C235Y}	123.6	13.42 to -
	H3.3^{G34R}, p53^{LOF}, ATRX^{LOF}, PDGFRA^{D842V}	4.873	3.253 to 8.168
	H3.3^{K27M}, p53^{LOF}	1.592 nM	0.7724 to 3.643 nM

	$H3.3^{K27M}$, $p53^{LOF}$, $PDGFRA^{WT}$	1.831	0.9015 to 4.163
	$H3.3^{K27M}$, $p53^{LOF}$, $PDGFRA^{D842V}$	1.642	0.9326 to 3.006
	$H3.3^{K27M}$, $FGFR1^{N457K}$	7.903	4.995 to 15.43
	$H3.3^{K27M}$, $p53^{LOF}$, $FGFR1^{N457K}$	4.359	2.821 to 7.623
	$H3.3^{K27M}$, $NF1^{LOF}$	1.439	0.8047 to 2.701
	$H3.3^{K27M}$, $NF1^{LOF}$, $FGFR1^{N457K}$	0.9861	0.7752 to 1.254
	$H3.3^{K27M}$, $PPM1D^{\Delta C}$, $PIK3CA^{E545K}$	1.032	0.6414 to 1.665
	$H3.1^{K27M}$, $p53^{LOF}$	3.197	1.726 to 7.260
	$H3.1^{K27M}$, $PIK3CA^{E545K}$, $ACVR1^{G328V}$	2.659	0.8876 to 16.31
GSK-J4	$H3.3^{G34R}$, $p53^{LOF}$, $ATRX^{LOF}$, $PDGFRA^{WT}$	39.58	13.45 to -
	$H3.3^{G34R}$, $p53^{LOF}$, $ATRX^{LOF}$, $PDGFRA^{C235Y}$	90.73	19.49 to -
	$H3.3^{G34R}$, $p53^{LOF}$, $ATRX^{LOF}$, $PDGFRA^{D842V}$	7.111	4.786 to 12.12
	$H3.3^{K27M}$, $p53^{LOF}$	1.038	0.2265 to 5.039
	$H3.3^{K27M}$, $p53^{LOF}$, $PDGFRA^{WT}$	5.074	1.447 to -
	$H3.3^{K27M}$, $p53^{LOF}$, $PDGFRA^{D842V}$	+ ∞	NA
	$H3.3^{K27M}$, $FGFR1^{N457K}$	4.263	2.911 to 6.821
	$H3.3^{K27M}$, $p53^{LOF}$, $FGFR1^{N457K}$	+ ∞	NA
	$H3.3^{K27M}$, $NF1^{LOF}$	127.3	14.24 to -
	$H3.3^{K27M}$, $NF1^{LOF}$, $FGFR1^{N457K}$	0.7821	0.1993 to 2.680
	$H3.3^{K27M}$, $PPM1D^{\Delta C}$, $PIK3CA^{E545K}$	0.6664	0.1276 to 2.494
	$H3.1^{K27M}$, $p53^{LOF}$	10.47	3.605 to -
	$H3.1^{K27M}$, $PIK3CA^{E545K}$, $ACVR1^{G328V}$	5.574	4.186 to 7.876
Idasanutlin	$H3.3^{G34R}$, $p53^{LOF}$, $ATRX^{LOF}$, $PDGFRA^{WT}$	2.824	1.840 to 4.865
	$H3.3^{G34R}$, $p53^{LOF}$, $ATRX^{LOF}$, $PDGFRA^{C235Y}$	3.623	2.348 to 6.479
	$H3.3^{G34R}$, $p53^{LOF}$, $ATRX^{LOF}$, $PDGFRA^{D842V}$	22.78	5.917 to -
	$H3.3^{K27M}$, $p53^{LOF}$	2.252	1.015 to 7.464
	$H3.3^{K27M}$, $p53^{LOF}$, $PDGFRA^{WT}$	6.275	3.941 to 12.82
	$H3.3^{K27M}$, $p53^{LOF}$, $PDGFRA^{D842V}$	2.623	1.393 to 6.643
	$H3.3^{K27M}$, $FGFR1^{N457K}$	1.558	0.5767 to 5.815
	$H3.3^{K27M}$, $p53^{LOF}$, $FGFR1^{N457K}$	55.43	5.158 to -
	$H3.3^{K27M}$, $NF1^{LOF}$	3.295	2.290 to 5.193
	$H3.3^{K27M}$, $NF1^{LOF}$, $FGFR1^{N457K}$	0.1200	0.07887 to 0.1857
	$H3.3^{K27M}$, $PPM1D^{\Delta C}$, $PIK3CA^{E545K}$	0.04732	0.03865 to 0.05772
	$H3.1^{K27M}$, $p53^{LOF}$	6.583	2.725 to -
	$H3.1^{K27M}$, $PIK3CA^{E545K}$, $ACVR1^{G328V}$	0.3268	0.09266 to 1.144
Infigratinib	$H3.3^{G34R}$, $p53^{LOF}$, $ATRX^{LOF}$, $PDGFRA^{WT}$	0.3125	0.2327 to 0.4161
	$H3.3^{G34R}$, $p53^{LOF}$, $ATRX^{LOF}$, $PDGFRA^{C235Y}$	0.01074	0.006420 to 0.01540
	$H3.3^{G34R}$, $p53^{LOF}$, $ATRX^{LOF}$, $PDGFRA^{D842V}$	0.3800	0.2863 to 0.5012
	$H3.3^{K27M}$, $p53^{LOF}$	0.3778	0.1969 to 0.6871
	$H3.3^{K27M}$, $p53^{LOF}$, $PDGFRA^{WT}$	0.4157	0.1801 to 0.8849
	$H3.3^{K27M}$, $p53^{LOF}$, $PDGFRA^{D842V}$	0.02958	0.008620 to 0.05991
	$H3.3^{K27M}$, $FGFR1^{N457K}$	0.2974	0.2250 to 0.3901
	$H3.3^{K27M}$, $p53^{LOF}$, $FGFR1^{N457K}$	0.3348	0.2469 to 0.4492
	$H3.3^{K27M}$, $NF1^{LOF}$	0.2326	0.06962 to 0.6414
	$H3.3^{K27M}$, $NF1^{LOF}$, $FGFR1^{N457K}$	0.1002	0.06164 to 0.1564
	$H3.3^{K27M}$, $PPM1D^{\Delta C}$, $PIK3CA^{E545K}$	0.5781	0.3042 to 1.080
	$H3.1^{K27M}$, $p53^{LOF}$	0.9282	0.6162 to 1.444
	$H3.1^{K27M}$, $PIK3CA^{E545K}$, $ACVR1^{G328V}$	0.3169	0.2062 to 0.4710
	GE NSC $H3.3^{WT}$	0.5299	0.3141 to 0.8966
	G477 ($H3.3^{WT}$) – human	2.266	1.765 to 3.011
	GBM-002 ($H3.3^{G34R}$) – human	0.4709	0.2626 to 0.8203

	GBM-002 G34R KO (H3.3^{G34R} KO) – human	0.2568	0.1012 to 0.5952
Trametinib	H3.3^{G34R}, p53^{LOF}, ATRX^{LOF}, PDGFRA^{WT}	0.01711	0.008319 to 0.03862
	H3.3^{G34R}, p53^{LOF}, ATRX^{LOF}, PDGFRA^{C235Y}	0.09461	0.08889 to 0.1007
	H3.3^{G34R}, p53^{LOF}, ATRX^{LOF}, PDGFRA^{D842V}	0.03260	0.02305 to 0.04615
	H3.3^{K27M}, p53^{LOF}	0.01827	0.005102 to 0.1045
	H3.3^{K27M}, p53^{LOF}, PDGFRA^{WT}	0.05263	0.02574 to 0.1046
	H3.3^{K27M}, p53^{LOF}, PDGFRA^{D842V}	0.1028	0.03278 to 0.3345
	H3.3^{K27M}, FGFR1^{N457K}	0.05553	0.04752 to 0.06479
	H3.3^{K27M}, p53^{LOF}, FGFR1^{N457K}	0.03489	0.02098 to 0.05765
	H3.3^{K27M}, NF1^{LOF}	0.008497	0.006677 to 0.01074
	H3.3^{K27M}, NF1^{LOF}, FGFR1^{N457K}	0.006592	0.003625 to 0.01116
	H3.3^{K27M}, PPM1D$^{\Delta C}$, PIK3CA^{E545K}	0.006059	0.004596 to 0.007836
	H3.1^{K27M}, p53^{LOF}	0.006737	0.005253 to 0.008527
	H3.1^{K27M}, PIK3CA^{E545K}, ACVR1^{G328V}	0.01371	0.002940 to 0.09282
	G477 (H3.3^{WT}) - human	0.07334	0.02912 to 0.1760
	SU-DIPG-VI (H3.3^{K27M}, p53$^{R34H/E66X}$, PDGFRA^{WT}) - human	20.53	8.347 to NA
	BT-869 (H3.3^{K27M}, PPM1D$^{\Delta C}$, PIK3CA^{E545K}, ACVR1^{R206H}, p53^{R272H}) - human	0.02472	0.005683 to 0.1439
	BT-416 (H3.3^{K27M}, p53^{R116Q}, NF1$^{L1607X/R2616X}$) - human	0.1549	0.009553 to 1.728

Supplementary Table 8. List of qPCR primers used in this study.

Primers	Sequence (5'-3')
Mus musculus ATRX	tctgctcttctttgcactcg<br>ttgactaaactgtaaaggaacttgga
Mus musculus p53	gacggaggtcgtgagacg<br>tttccttccacccggatac
Mus musculus Nf1	ctggaatcctgacgctcctg<br>aggagcttctatctgcctgctta
Mus musculus Dlx1	catcagttccgtgcagtcctac<br>cctttgccgttaaagcgcacct
Mus musculus Dlx2	gtctcctactccgccaaaagca<br>ggatttcaggctcaaggtcttcc
Mus musculus B2M	gtgaccctggtctttctggt<br>gtatgttcggcttcccattc

Supplementary Table 9. Antibodies used for Western blotting, immunofluorescence, and immunohistochemistry.

Antibodies	Application (Dilution)	Source and reference
**Primaries**		
ACVR1	IF (1:200),<br>WB (1:1000)	Abcam Rabbit Monoclonal - ab155981 RRID:AB_2929006<br>R&D Systems Mouse Monoclonal IgG1 - MAB637 RRID:AB_2221997
ATRX	IF (1:200),<br>WB (1:500)	Abcam Rabbit Polyclonal - ab97508 RRID:AB_10680289<br>Santa Cruz (D-5) Mouse Monoclonal IgG2a - sc-55584 RRID:AB_831012
CCND2	IF (1:200),<br>WB (1:500)	Santa Cruz (34B1-3) Rat Monoclonal IgG_{2a} - sc-452 RRID:AB_627350
CD31	IF (1:30)	BD Pharmingen Rat Monoclonal – 550274 RRID:AB_393571
FGFR1	IF (1:100),<br>WB (1:1000)	Cell Signaling Rabbit Monoclonal (D8E4) - #9740 RRID:AB_11178519
GAPDH	WB (1:1000)	Proteintech Mouse Monoclonal IgG_{2b} - 60004-1-Ig RRID:AB_2107436
GFAP	IF (1:500)	Millipore Mouse Monoclonal IgG_1 - MAB360 RRID:AB_11212597
GFP	IF (1:500),<br>IHC (10 μg/ml)	Abcam Chicken Polyclonal - ab13970 RRID:AB_300798
H3	WB (1:10,000)	Abcam Rabbit Polyclonal - ab1791 RRID:AB_302613
H3.3	WB (1:1000)	Millipore Rabbit Polyclonal - 09-838 RRID:AB_10845793
H3.3 G34R	IF (1:1000), WB (1:500), IHC (1:1000)	RevMAb Biosciences (RM240) Rabbit Monoclonal - 31-1120-00 RRID:AB_2716433
H3.3 K27M	IF (1:1000), WB (1:500), IHC (1:1000)	RevMAb Biosciences (RM192) Rabbit Monoclonal - 31-1175-00 RRID:AB_2716432
H3 K27me3	IF (1:500),<br>WB (1:1000)	Millipore Rabbit Polyclonal - 07-449 RRID:AB_310624
HA	IF (1:500),<br>WB (1:1000)	Abcam Rabbit Polyclonal - ab9110 RRID:AB_307019<br>Santa Cruz Mouse Monoclonal (F-7) IgG_{2a} - sc-7392 RRID:AB_627809
Ki67	IF (1:500),<br>IHC (1:1000)	Abcam Rabbit Polyclonal - ab15580 RRID:AB_443209<br>Bethyl Laboratories Rabbit Polyclonal - IHC-00375 RRID:AB_1547959
Nestin	IF (1:200)	Millipore Mouse Monoclonal IgG_1 - MAB353 RRID:AB_94911
Olig2	IF (1:500)	Millipore Rabbit Polyclonal - AB9610 RRID:AB_570666
PDGFRA	IF (1:10),<br>WB (1:1000)	R&D Systems Goat Polyclonal - AF1062 RRID:AB_2236897
PIK3CA	IF (1:100),<br>WB (1:1000)	Novus Bio Rabbit Polyclonal - NBP2-19804 RRID:AB_2929008<br>Abcam Rabbit Monoclonal - ab40776 RRID:AB_777253

PPM1D	IF (1:200), WB (1:500)	Novus Bio Rabbit Polyclonal - NBP1-87249 RRID:AB_11008859<br>Santa Cruz Mouse Monoclonal (F-10) IgG1 - sc-376257 RRID:AB_10986000
Sox2	IF (1:200)	R&D Systems Goat Polyclonal - AF2018 RRID:AB_355110
α-Tubulin	WB (1:1000)	Proteintech Mouse Monoclonal IgG_{2b} - 66031-1-Ig RRID:AB_11042766
V5	IF (1:1000)	Cell Signaling Rabbit Monoclonal (D3H8Q) - #13202 RRID:AB_2687461
**Secondaries**		
Hoechst 33342	IF (1:2000)	ThermoFisher Scientific H3570
Alexa Fluor 488 Donkey anti-Chicken IgG	IF (1:1000)	Jackson Immunoresearch 703-545-155 RRID:AB_2340375
Alexa Fluor 555 Donkey anti-Rabbit IgG	IF (1:1000)	ThermoFisher Scientific A31572 RRID:AB_162543
Alexa Fluor 594 Donkey anti-Goat IgG	IF (1:1000)	ThermoFisher Scientific A11058 RRID:AB_2534105
Alexa Fluor 647 Goat anti-Mouse IgG_1	IF (1:1000)	ThermoFisher Scientific A21240 RRID:AB_2535809
Alexa Fluor 647 Goat anti-Mouse IgG_{2a}	IF (1:1000)	ThermoFisher Scientific A21241 RRID:AB_2535810
Alexa Fluor 647 Donkey anti-Goat IgG	IF (1:1000)	ThermoFisher Scientific A32849 RRID:AB_2762840
Alexa Fluor 647 Donkey anti-Rabbit IgG	IF (1:1000)	Jackson Immunoresearch 711-605-152 RRID:AB_2492288
IRDye® 800CW Donkey anti-Rabbit IgG	WB (1:10,000)	LI-COR Biosciences - 926-32213 RRID:AB_621848
IRDye® 680RD Donkey anti-Mouse IgG	WB (1:10,000)	LI-COR Biosciences - 926-68072 RRID:AB_10953628
IRDye® 800CW Donkey anti-Goat IgG	WB (1:10,000)	LI-COR Biosciences - 926-32214 RRID:AB_621846
IRDye® 800CW Goat anti-Rat IgG	WB (1:10,000)	LI-COR Biosciences - 926-32219 RRID:AB_1850025
