# Supplementary material for: A Compendium of Syngeneic, Transplantable Pediatric High-Grade Glioma Models Reveals Subtype-Specific Therapeutic Vulnerabilities
Source: Cancer Discov. 2023 Apr 3;13(7):1592–615. doi: 10.1158/2159-8290.CD-23-0004 (PMC10326601; doi:10.1158/2159-8290.CD-23-0004)
Supplement: Supplementary Figures 1-27 — Figures describing hindbrain vs ganglionic eminence targeting strategies, each of the 16 models generated and validation that they expressed the introduced mutations, survival of GE vs CTX-electroporated embryos, and cell viability data shown in bar graph format. [file cd-23-0004_supplementary_figures_1-27_suppsf1.pdf]

Supplementary Figure 1: ***In vivo*** **validation of tag (HA and V5) and reporter (GFP and Akaluc) expression**

A

C-terminal HA tag reveals histone mutant expression *in vivo*
GFP is expressed from the same vector via a PQR 2A peptide

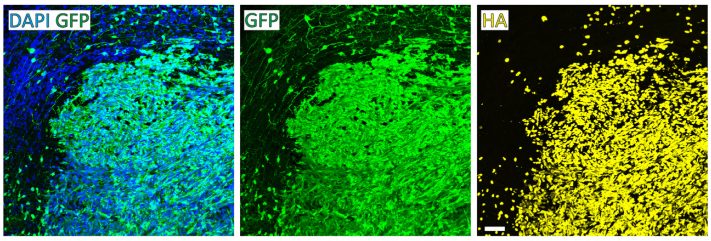

B

V5 tag expressing cells are apparent in HA⁺/GFP⁺ cells *in vivo*

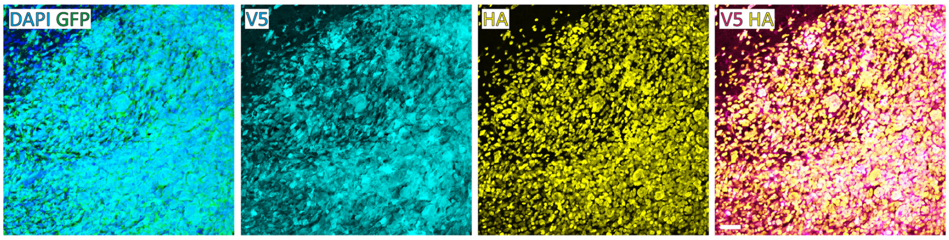

C

Akaluc allows non-invasive imaging of tumors in C57BL/6J mice without shaving or depilation

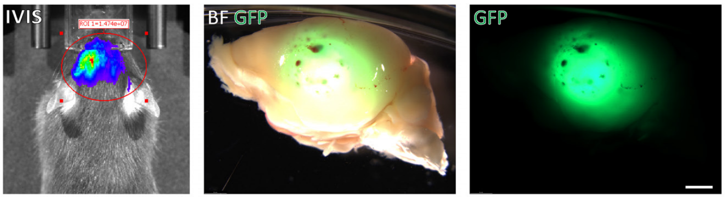

Supplementary Figure 1

A piggyBac vectors express an HA-tagged mutant histone and drive GFP expression downstream from a PQR 2A peptide. Following embryonic delivery of this vector into neural progenitor cells, GFP appears cytoplasmic and HA is nuclear, indicating that the peptides are efficiently cleaved and the mutant histone is incoroporated into chromatin.
B Partner alteration-encoding vectors express V5-tagged EBFP upstream from a PQR 2A peptide. V5 can be readily detected in every GFP^{+} tumor cell, indicating robust co-expression of the introduced mutations. **C** (Left) Introducing piggyBac Akaluc into the cocktail of elecporated constructs enables detection and monitoring of developing tumors. Akaluc bioluminescence is 100 to 1000 times brighter than convetional luciferases and allows non-invasive imaging of tumors in C57BL/6J mice without the need for shaving or depilation. (Middle and Right) The dissected tumor from the same animal imaged on the left, showing a large GFP^{+} tumor. Scale bars represent 1 mm in low magnification panels and 50 μm in high magnification panels.

Supplementary Figure 2: Ganglionic eminences vs cortical *in utero* electroporation

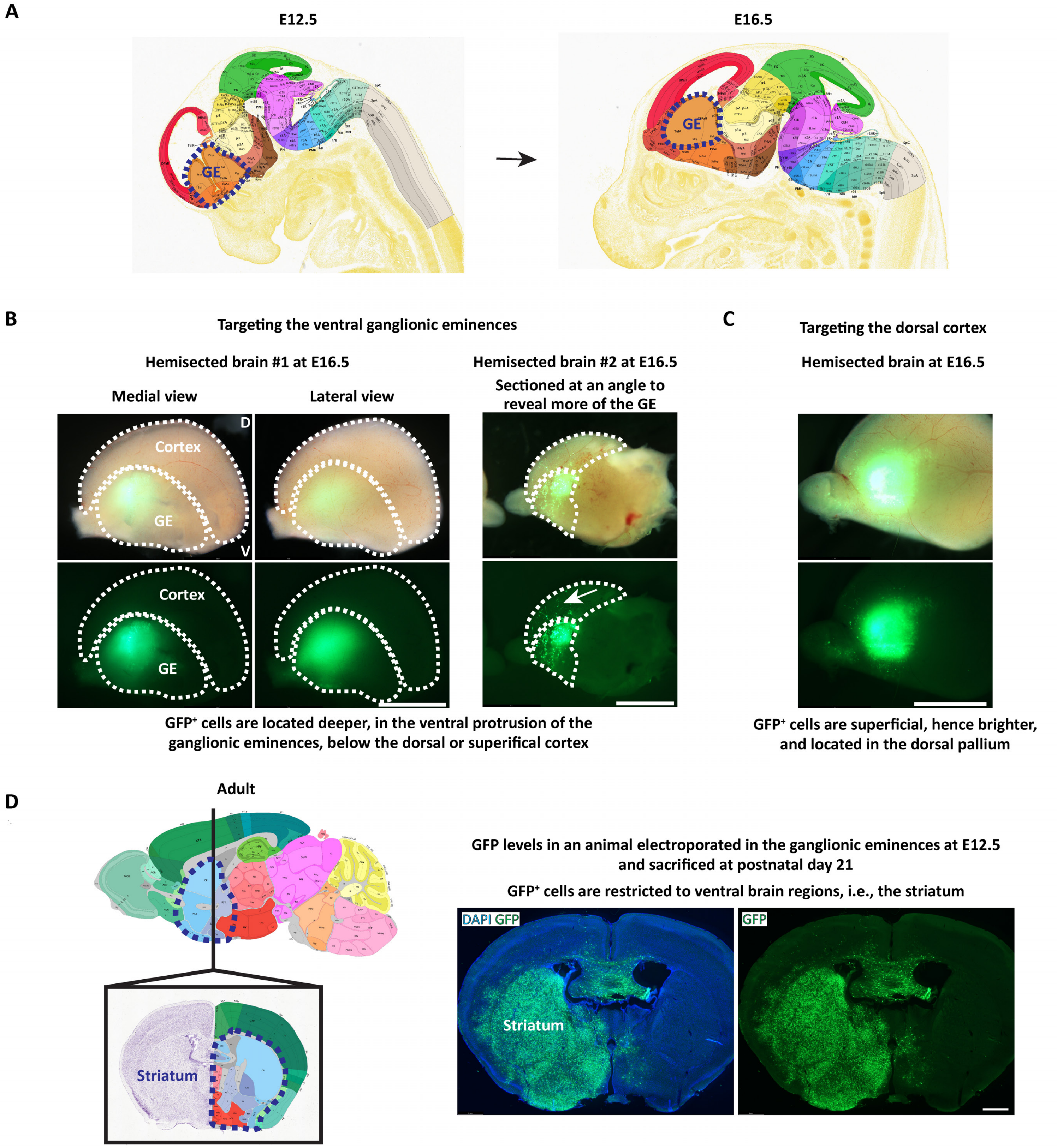

Supplementary Figure 2

A Schematic showing location of the ganglionic eminences in the sagittal plane in E12.5 and E16.5 embryos. **B** (Left) E16.5 brain electroporated in the ganglionic eminences at E12.5 (96h post-IUE). Medial and lateral views are depicted, with GFP^{+} cells apparent in the deeper, ventral protrusion of the lateral ganglionic eminence. (Right) A second example of an E16.5 brain electroporated in the ganglionic eminences at E12.5. This brain has been cut at an angle to reveal more of the ventral protrusion of the ganglionic eminences. Individual GFP^{+} interneuron progenitors are also apparent and have begun tangential migration into the cortex (arrow). Scale bares represent 1 mm. **C** E16.5 brain electroporated in the dorsal cortex at E12.5. GFP^{+} cells are superficially located, i.e., electroporation occurred into dorsal cortical layers. Scale bares represent 1 mm. **D** (Left) Schematic showing the location of the striatum in postnatal/adult mouse brain. The black bar indicates the approximate location of coronal sections shown in Figure 1. (Right) Immunofluorescence for GFP and DAPI in a coronal forebrain section from a weaning-age animal (postnatal day 21), electroporated in the ganglionic eminences at E12.5. Electroporated vectors encoded H3.3^{G34R}, ATRX^{LOF}, p53^{LOF} and PDGFRA^{WT}. Most GFP^{+} cells remain trapped in the ventral forebrain. Scale bars represent 1 mm. Reference atlas images downloaded from the Allen Institute's https://mouse.brain-map.org/static/atlas.

Supplementary Figure 3: H3.3^{G34R}, p53^{LOF}, ATRX^{LOF}, PDGFRA^{WT}

GPAP

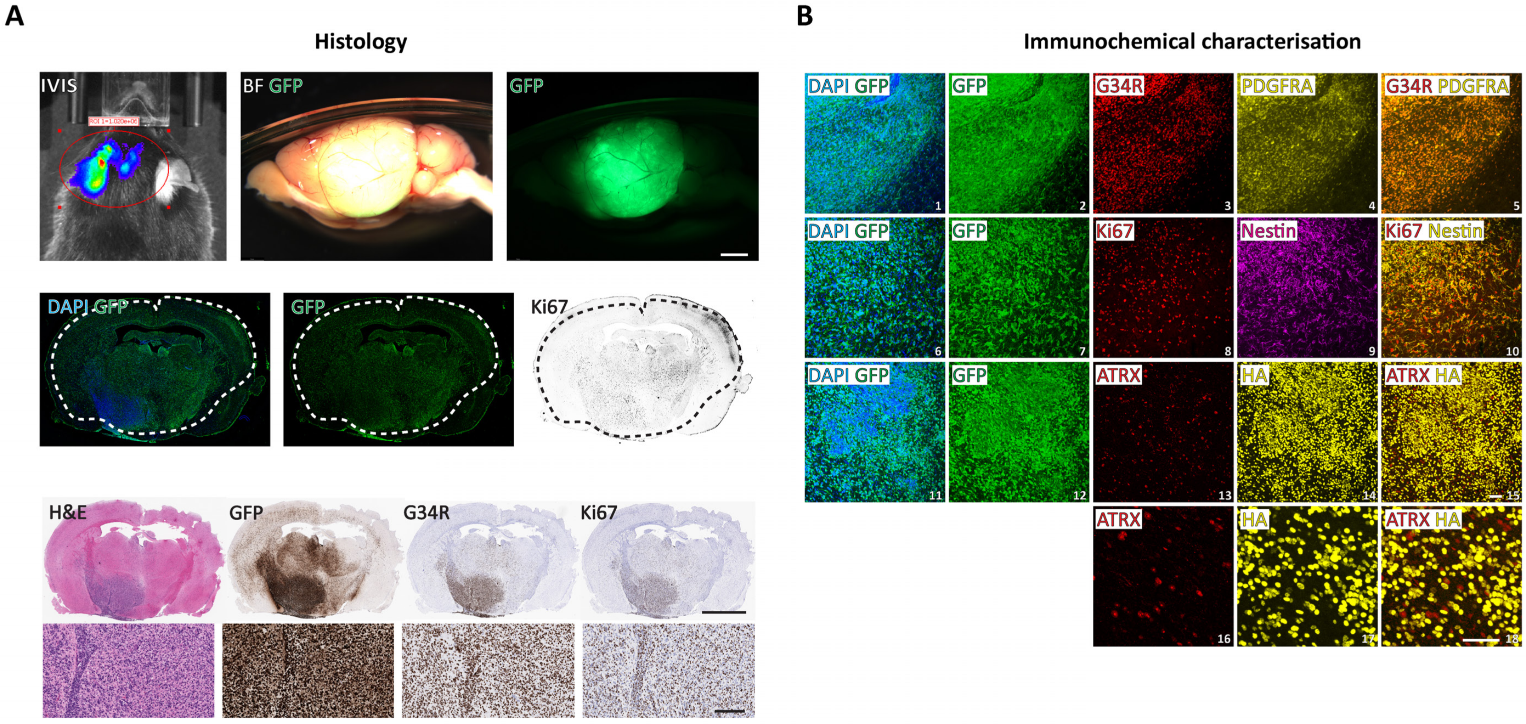

Supplementary Figure 3

A (Top) Bioluminescence imaging and low magnification view of a GPAP tumor in a symptomatic animal. Scale bars represent 1 mm. (Middle) Coronal section through the tumor and immunofluorescence for DAPI, GFP and Ki67. Scale bars represent 1 mm. (Bottom) Coronal section through the tumor and H&E staining and immunohistochemical detection of GFP, G34R and Ki67. Scale bars represent 2 mm and 200 µm. **B** Immunofluorescence to detect levels of G34R, PDGFRA, Ki67, Nestin, ATRX and HA in the tumor. Insets below show higher magnification views of ATRX levels in HA$^+$ nuclei. Scale bars represent 50 µm.

Supplementary Figure 4: H3.3^{G34R}, p53^{LOF}, ATRX^{LOF}, PDGFRA^{D842V}

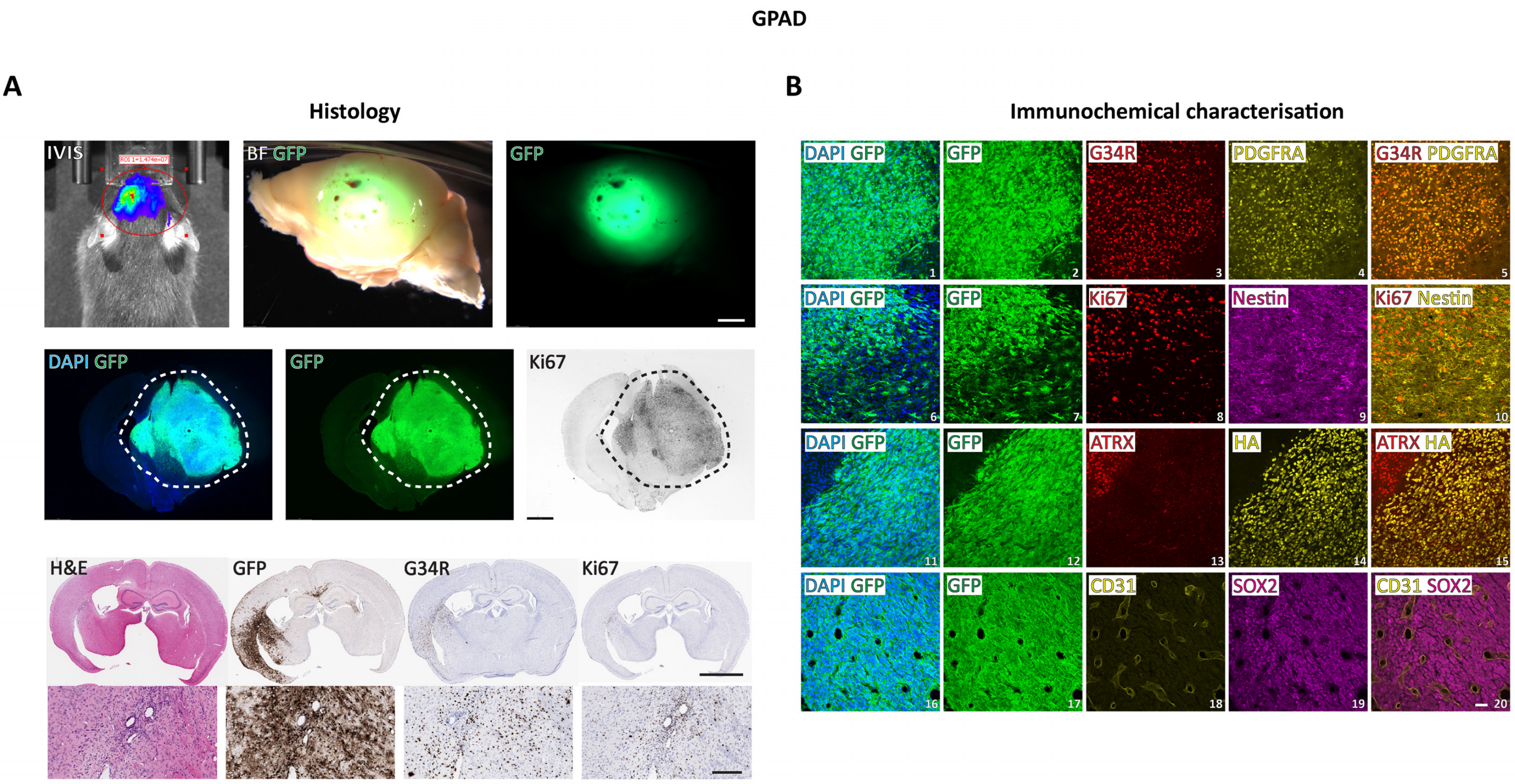

Supplementary Figure 4

A (Top) Bioluminescence imaging and low magnification view of a GPAD tumor in a symptomatic animal. Scale bars represent 1 mm. (Middle) Coronal section through the tumor and immunofluorescence for DAPI, GFP and Ki67. Scale bars represent 1 mm. (Bottom) Coronal section through the tumor and H&E staining and immunohistochemical detection of GFP, G34R and Ki67. Scale bars represent 2 mm and 200 μm. **B** Immunofluorescence to detect levels of G34R, PDGFRA, Ki67, Nestin, ATRX, HA, CD31 and Sox2 in the tumor. Scale bars represent 50 μm.

Supplementary Figure 5: H3.3^{G34R}, p53^{LOF}, ATRX^{LOF}, PDGFRA^{C235Y}

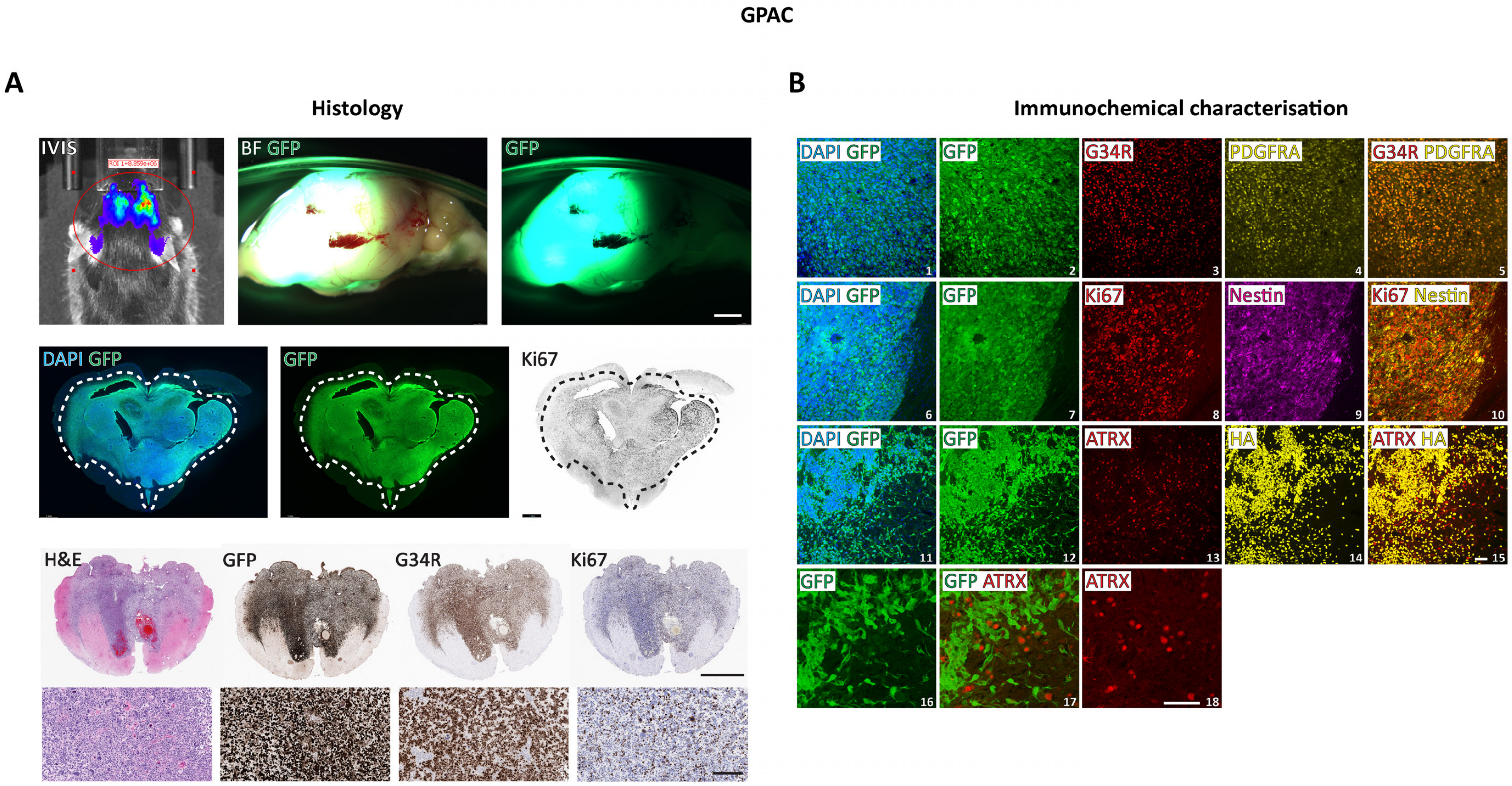

Supplementary Figure 5

A (Top) Bioluminescence imaging and low magnification view of a GPAC tumor in a symptomatic animal. Scale bars represent 1 mm. (Middle) Coronal section through the tumor and immunofluorescence for DAPI, GFP and Ki67. Scale bars represent 1 mm. (Bottom) Coronal section through the tumor and H&E staining and immunohistochemical detection of GFP, G34R and Ki67. Scale bars represent 2 mm and 200 µm. **B** Immunofluorescence to detect levels of G34R, PDGFRA, Ki67, Nestin, ATRX and HA in the tumor. Insets below show higher magnification views of ATRX levels in GFP^{+} nuclei. Scale bars represent 50 µm.

Supplementary Figure 6: H3.3^{G34R}, p53^{LOF}, ATRX^{LOF}

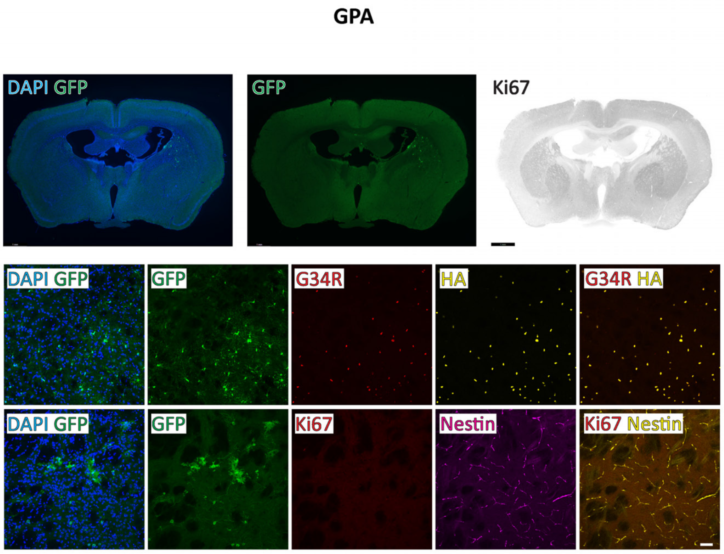

Supplementary Figure 6

(Top) Coronal section through the brain of an animal electroporated with constructs encoding H3.3^{G34R}, ATRX^{LOF} and p53^{LOF}. Immunofluorescence for DAPI, GFP and Ki67 reveals the absence of a tumor with this combination of mutations. Scale bars represent 1 mm. (Bottom) Immunofluorescence to detect levels of G34R, HA, Ki67 and Nestin. Scale bars represent 50 μm.

Supplementary Figure 7: Selective effects of PDGFRA C235Y in the ganglionic eminences are mitigated by H3.3G34R

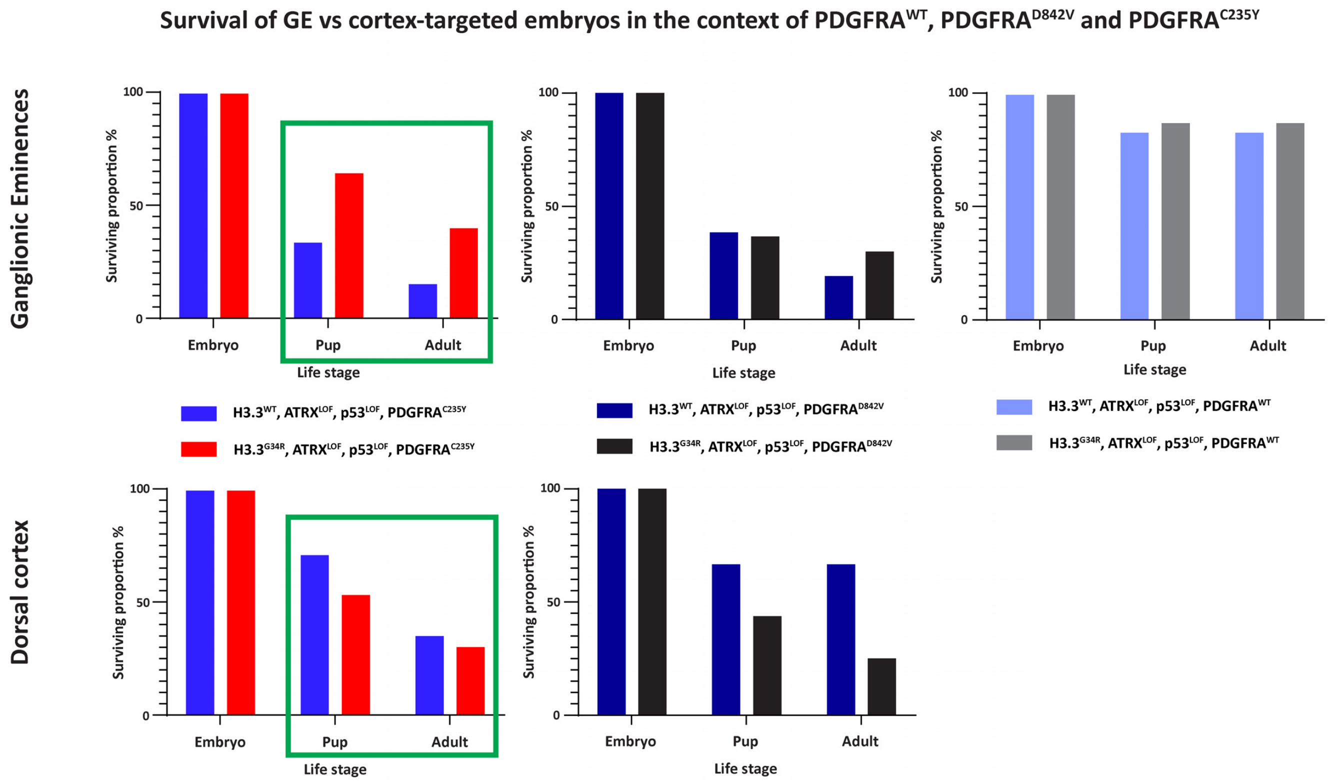

Supplementary Figure 7

When introduced into the GE or cortex, PDGFRA^{C235Y} and PDGFRA^{D842V} both produce a degree of embryonic lethality (bar graphs in the left and middle panels), not seen with PDGFRA^{WT} (bar graph on the extreme right). However, co-introduction of H3.3^{G34R} with PDGFRA^{C235Y} improves survival in the GE, relative to H3.3^{WT} (data highlighted in green boxes). This improvement in survival is not seen in cortex, or with PDGFRA^{D842V} in either location, suggesting that H3.3^{G34R} promotes survival of PDGFRA^{C235Y}-expressing cells in a niche-dependent manner.

Supplementary Figure 8: Drug screening in G34R lines presented as bar graphs

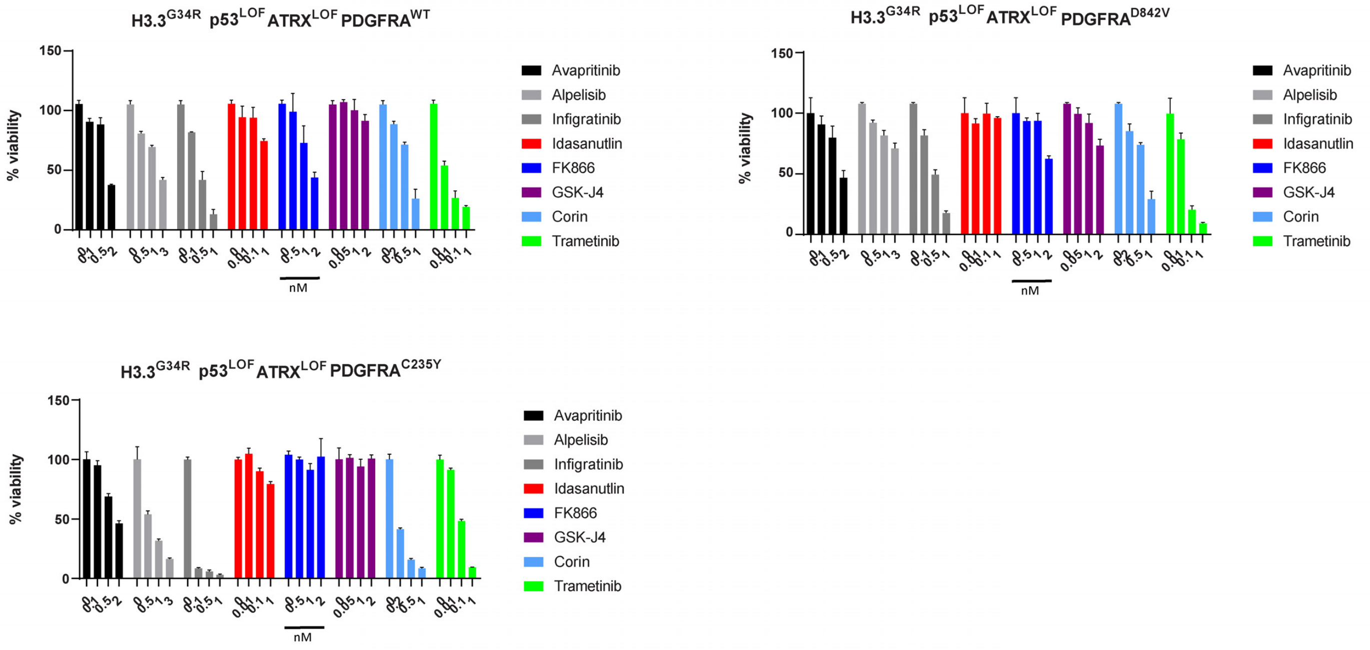

Supplementary Figure 8

Drug screening data represented as bar graphs, showing sensitivity of GPAP, GPAD and GPAC cells to infigratinib (Figure 1G) as well 7 other inhibitors. Note the enhanced sensitivity to infigratinib specifically in GPAC cells, and the reduced sensitivity to avapritinib (compared to K27M models which are more sensitive to avapritinib).

Supplementary Figure 9: Hindbrain *in utero* electroporation

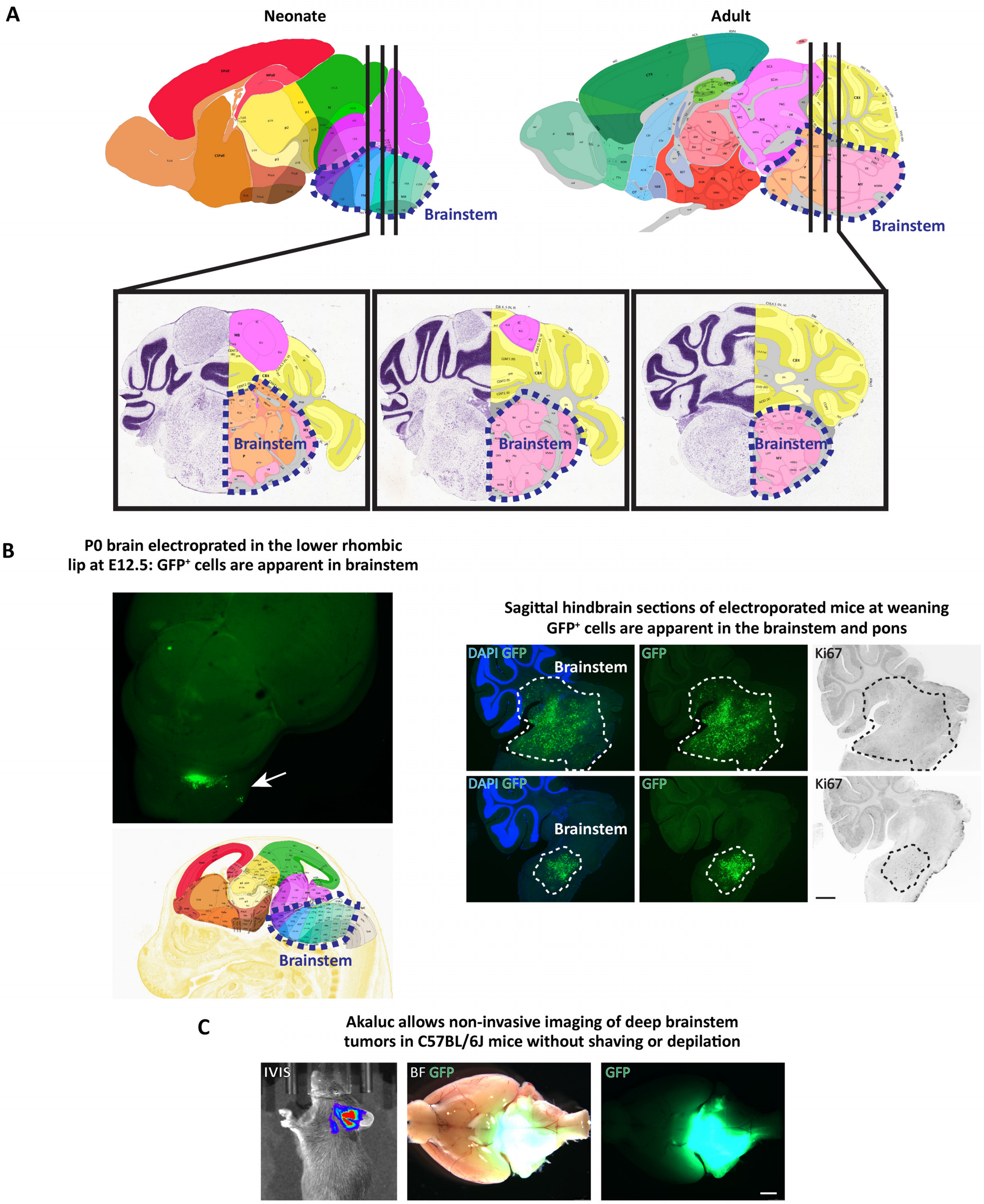

Supplementary Figure 9

A Schematic showing location of the brainstem in the sagittal and coronal planes in neonatal and adult mouse brain. The black bars indicate the approximate location of coronal sections shown in Figures 2, 4 and 6. **B** (Left) Representative fluorescence image of a postnatal day 0 (P0) brain electroporated in the lower rhombic lip (LRL) at E12.5. GFP$^+$ cells can be seen below the cerebellum, where the LRL would have been at E12.5, before the upper rhombic lip expanded and gave rise to the cerebellum. Schematic outlining embryonic LRL location below. (Right) Sagittal sections through the brainstem in weaning-age electroporated mice, showing GFP$^+$/Ki67$^+$ preneoplastic lesions developing in the brainstem and pons. **C** (Left) Introducing piggyBac Akaluc into the cocktail of elecporated constructs enables detection and monitoring of developing tumors. Akaluc bioluminescence is 100 to 1000 times brighter than convetional luciferases and allows non-invasive imaging of deep-seated brainstem tumors in C57BL/6J mice without the need for shaving or depilation. (Middle and Right) The dissected tumor from the same animal imaged on the left, showing a large GFP$^+$ tumor located in the brainstem. Scale bars represent 1 mm in low magnification panels and 50 μm in high magnification panels. Reference atlas images downloaded from the Allen Institute's https://mouse.brain-map.org/static/atlas.

Supplementary Figure 10: H3.3^{K27M}, p53^{LOF}, PDGFRA^{WT}

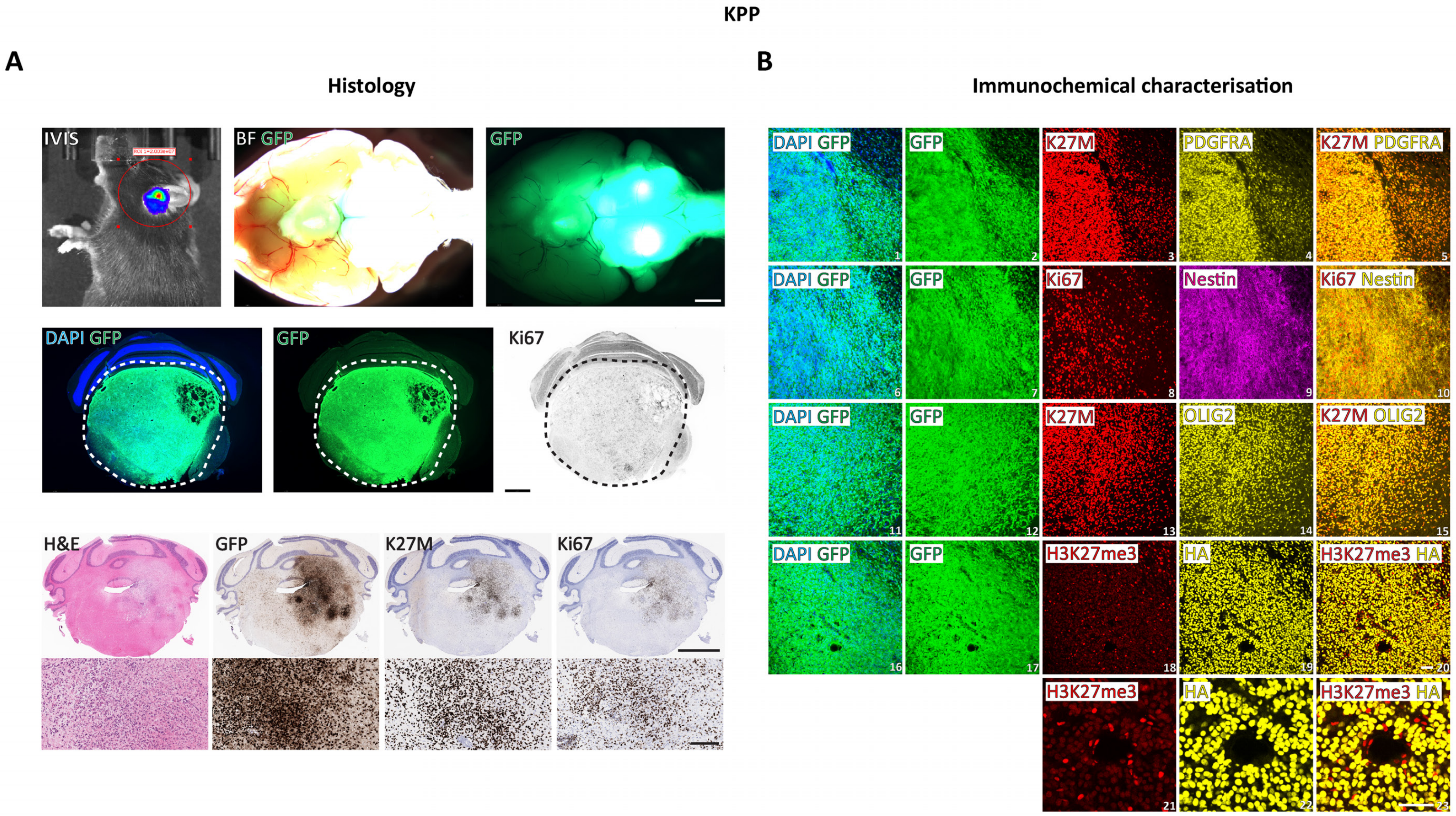

Supplementary Figure 10

A (Top) Bioluminescence imaging and low magnification view of a KPP tumor in a symptomatic animal. Scale bars represent 1 mm. (Middle) Coronal section through the tumor and immunofluorescence for DAPI, GFP and Ki67. Scale bars represent 1 mm. (Bottom) Coronal section through the tumor and H&E staining and immunohistochemical detection of GFP, K27M and Ki67. Scale bars represent 2 mm and 200 μm. **B** Immunofluorescence to detect levels of K27M, PDGFRA, Ki67, Nestin, Olig2, H3K27me3 and HA in the tumor. Insets below show higher magnification views of H3K27me3 levels in HA^{+} nuclei. Scale bars represent 50 μm.

Supplementary Figure 11: H3.1^{K27M}, ACVR1^{G328V}, PIK3CA^{E545K}

H3.1KACVPIK

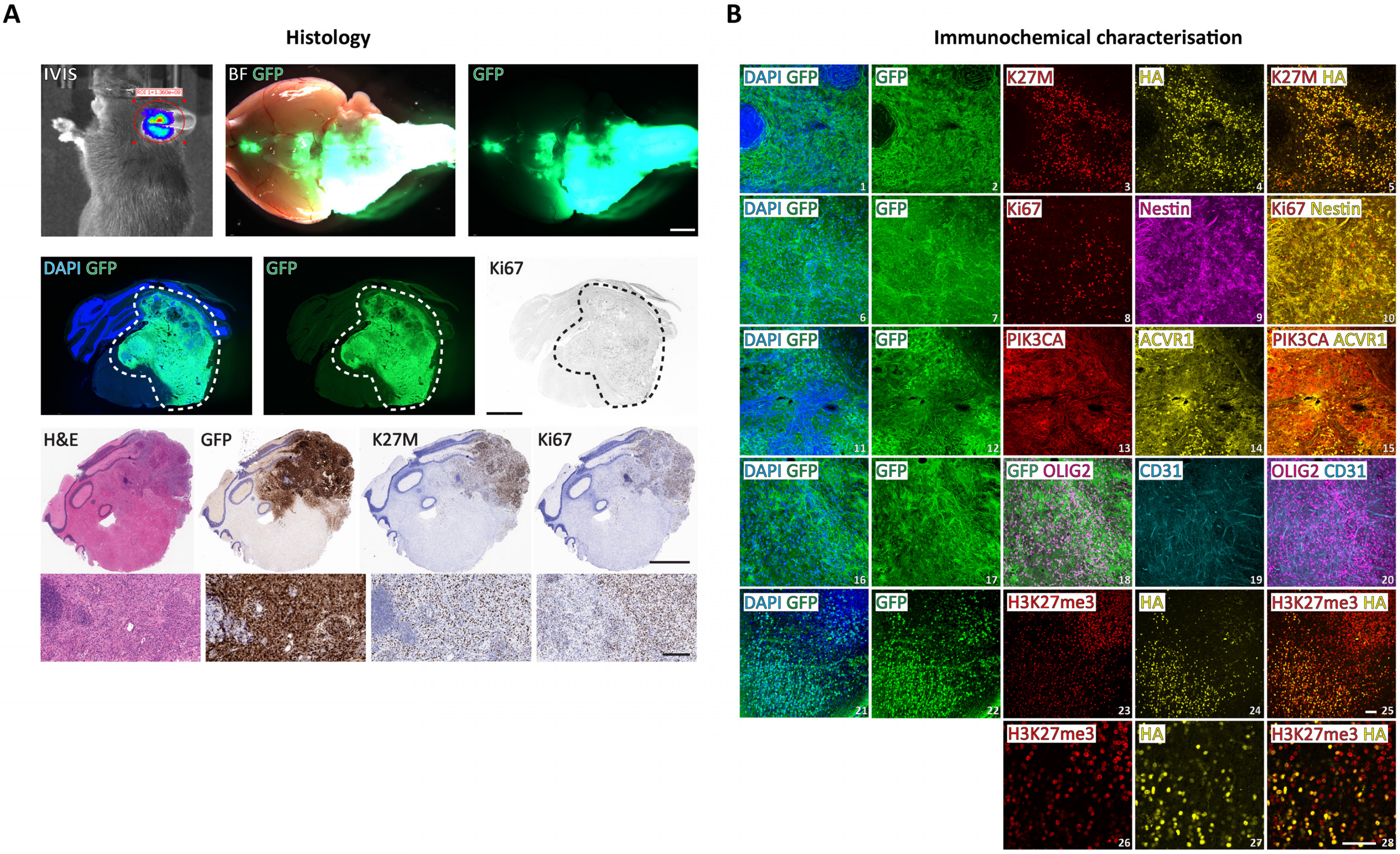

Supplementary Figure 11
A (Top) Bioluminescence imaging and low magnification view of an H3.1KACVPIK tumor in a symptomatic animal. Scale bars represent 1 mm. (Middle) Coronal section through the tumor and immunofluorescence for DAPI, GFP and Ki67. Scale bars represent 1 mm. (Bottom) Coronal section through the tumor and H&E staining and immunohistochemical detection of GFP, K27M and Ki67. Scale bars represent 2 mm and 200 µm. **B** Immunofluorescence to detect levels of K27M, Ki67, Nestin, PIK3CA, ACVR1, Olig2, CD31, H3K27me3 and HA in the tumor. Insets below show higher magnification views of H3K27me3 levels in HA^{+} nuclei. Scale bars represent 50 µm.

Supplementary Figure 12: H3.3^{K27M}, PPM1D$^{\Delta C}$, PIK3CA^{E545K}

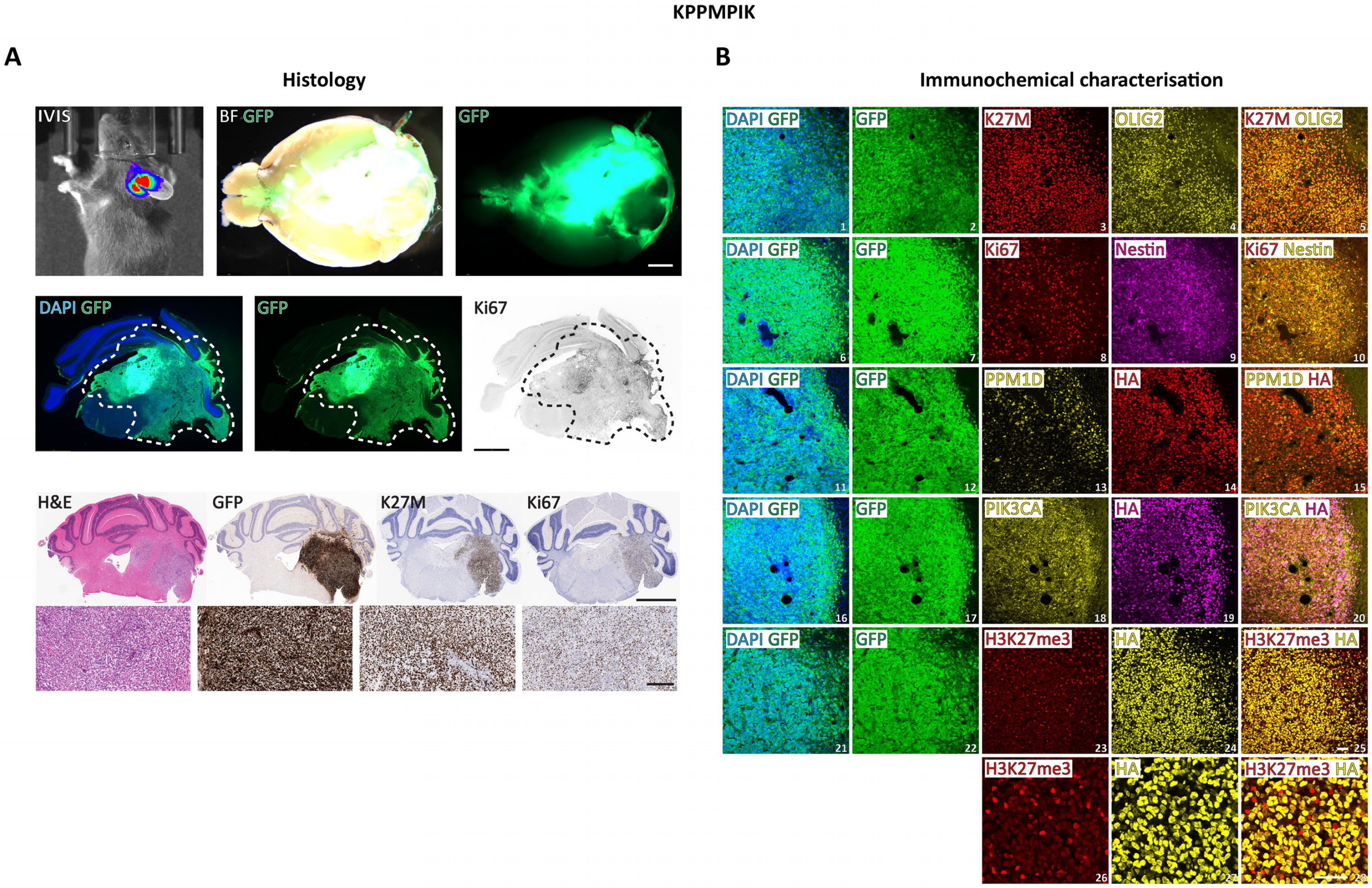

Supplementary Figure 12

A (Top) Bioluminescence imaging and low magnification view of a KPPMPIK tumor in a symptomatic animal. Scale bars represent 1 mm. (Middle) Coronal section through the tumor and immunofluorescence for DAPI, GFP and Ki67. Scale bars represent 1 mm. (Bottom) Coronal section through the tumor and H&E staining and immunohistochemical detection of GFP, K27M and Ki67. Scale bars represent 2 mm and 200 µm. **B** Immunofluorescence to detect levels of K27M, Olig2, Ki67, Nestin, PPM1D, PIK3CA, H3K27me3 and HA in the tumor. Insets below show higher magnification views of H3K27me3 levels in HA^{+} nuclei. Scale bars represent 50 µm.

Supplementary Figure 13: H3.3^{K27M}, NF1^{LOF}, FGFR1^{N457K}

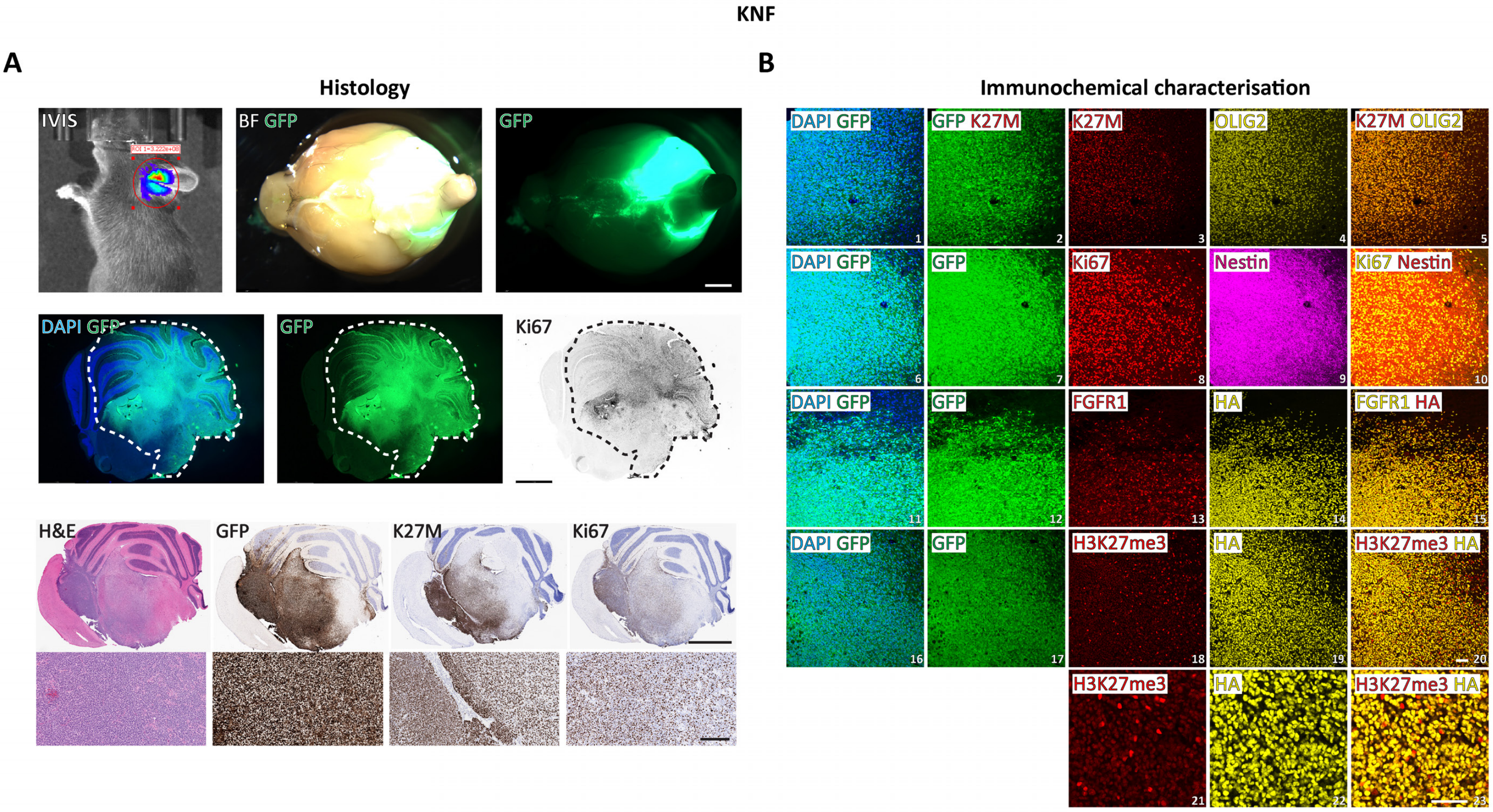

Supplementary Figure 13

A (Top) Bioluminescence imaging and low magnification view of a KNF tumor in a symptomatic animal. Scale bars represent 1 mm. (Middle) Coronal section through the tumor and immunofluorescence for DAPI, GFP and Ki67. Scale bars represent 1 mm. (Bottom) Coronal section through the tumor and H&E staining and immunohistochemical detection of GFP, K27M and Ki67. Scale bars represent 2 mm and 200 µm. **B** Immunofluorescence to detect levels of K27M, Olig2, Ki67, Nestin, FGFR1, H3K27me3 and HA in the tumor. Insets below show higher magnification views of H3K27me3 levels in HA^{+} nuclei. Scale bars represent 50 µm.

Supplementary Figure 14: H3.3^{K27M} alone

K

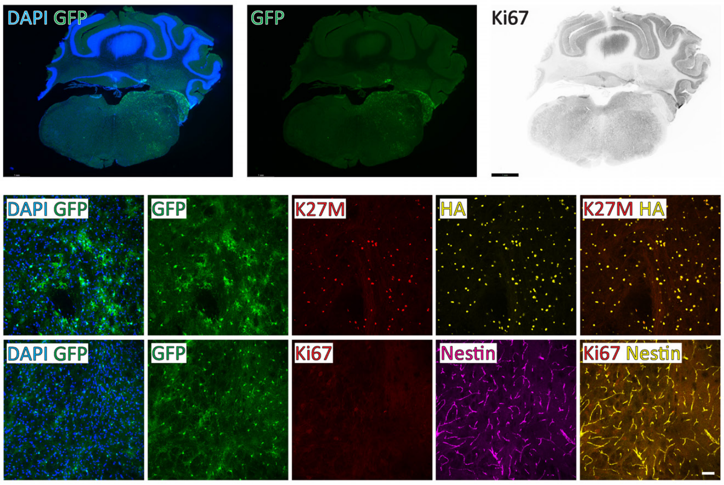

Supplementary Figure 14
(Top) Coronal section through the brain of an animal electroporated with a construct encoding H3.3^{K27M}. Immunofluorescence for DAPI, GFP and Ki67 reveals the absence of a tumor with this mutation alone. Scale bars represent 1 mm. (Bottom) Immunofluorescence to detect levels of K27M, HA, Ki67 and Nestin. Scale bars represent 50 μm.

Supplementary Figure 15: H3.1^{K27M} alone

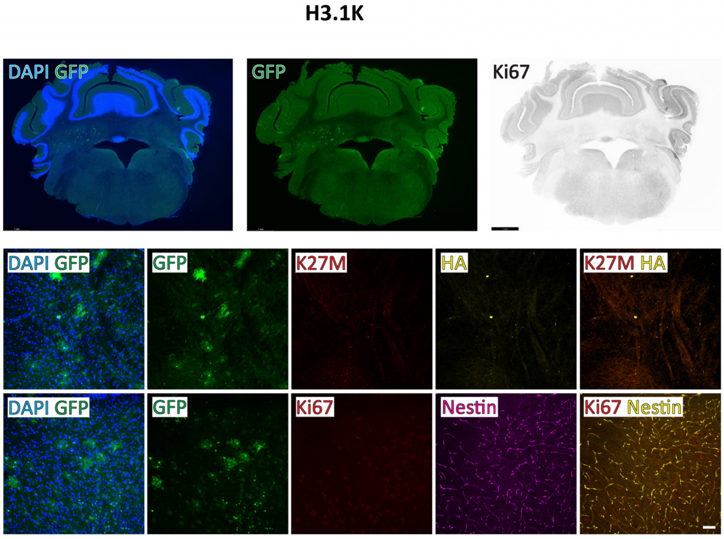

Supplementary Figure 15

(Top) Coronal section through the brain of an animal electroporated with a construct encoding H3.1^{K27M}. Immunofluorescence for DAPI, GFP and Ki67 reveals the absence of a tumor with this mutation alone. Scale bars represent 1 mm. (Bottom) Immunofluorescence to detect levels of K27M, HA, Ki67 and Nestin. The K27M antibody does not detect H3.1^{K27M} as well as H3.3^{K27M}, however, K27M^{+} cells are visible and co-localize with HA. Scale bars represent 50 μm.

Supplementary Figure 16: Single-nuclei RNA sequencing quality control, dimension reduction, clustering, and automated annotation

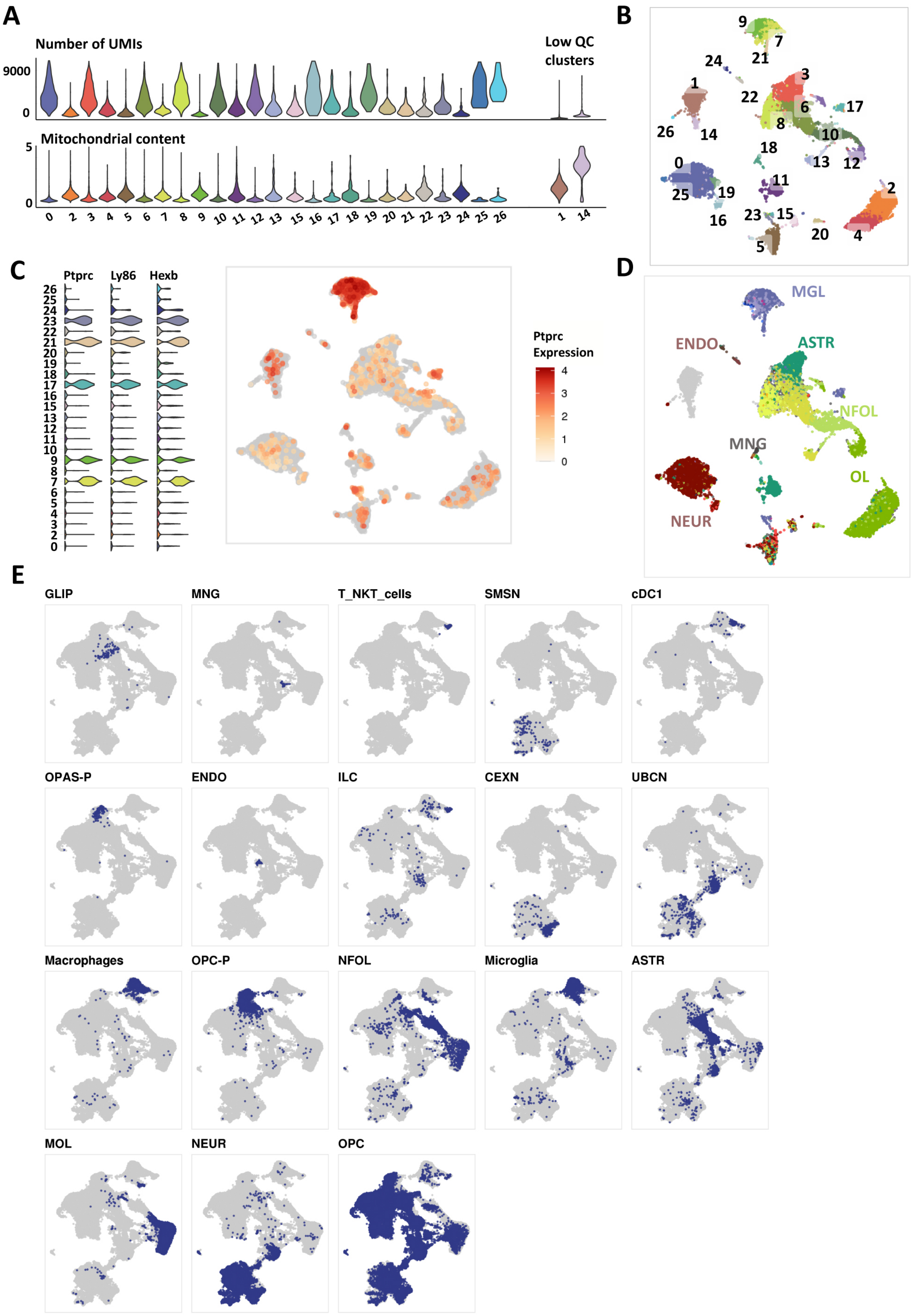

Supplementary Figure 16

A Distribution of number of detected genes and percent of mitochondrial transcripts per cluster for representative sample KPP replicate 2. Low quality clusters shown to the right. **B** UMAP of KPP replicate 2, with cells colored by cluster. **C** Distribution of immune marker gene detection per cluster of KPP replicate 2 (left) and UMAP as in B with cells colored by normalized Ptprc expression (right). **D** UMAP as in B with cells colored by cell type annotation. Low quality clusters are shown in grey. **E** UMAPs of KNF and KPP integration highlighting cell types with at least 100 cells. ASTR, astrocyte; ENDO, endothelial cell; MGL, microglia; MNG, meninges; NEUR, neuron; NFOL, newly-forming oligodendrocyte; OPC, oligodendrocyte precursor cell; OL, oligodendrocyte.

Supplementary Figure 17: Single-nuclei RNA sequencing of KPPMPIK, and inferred TF activity scoring of KNF vs KPP

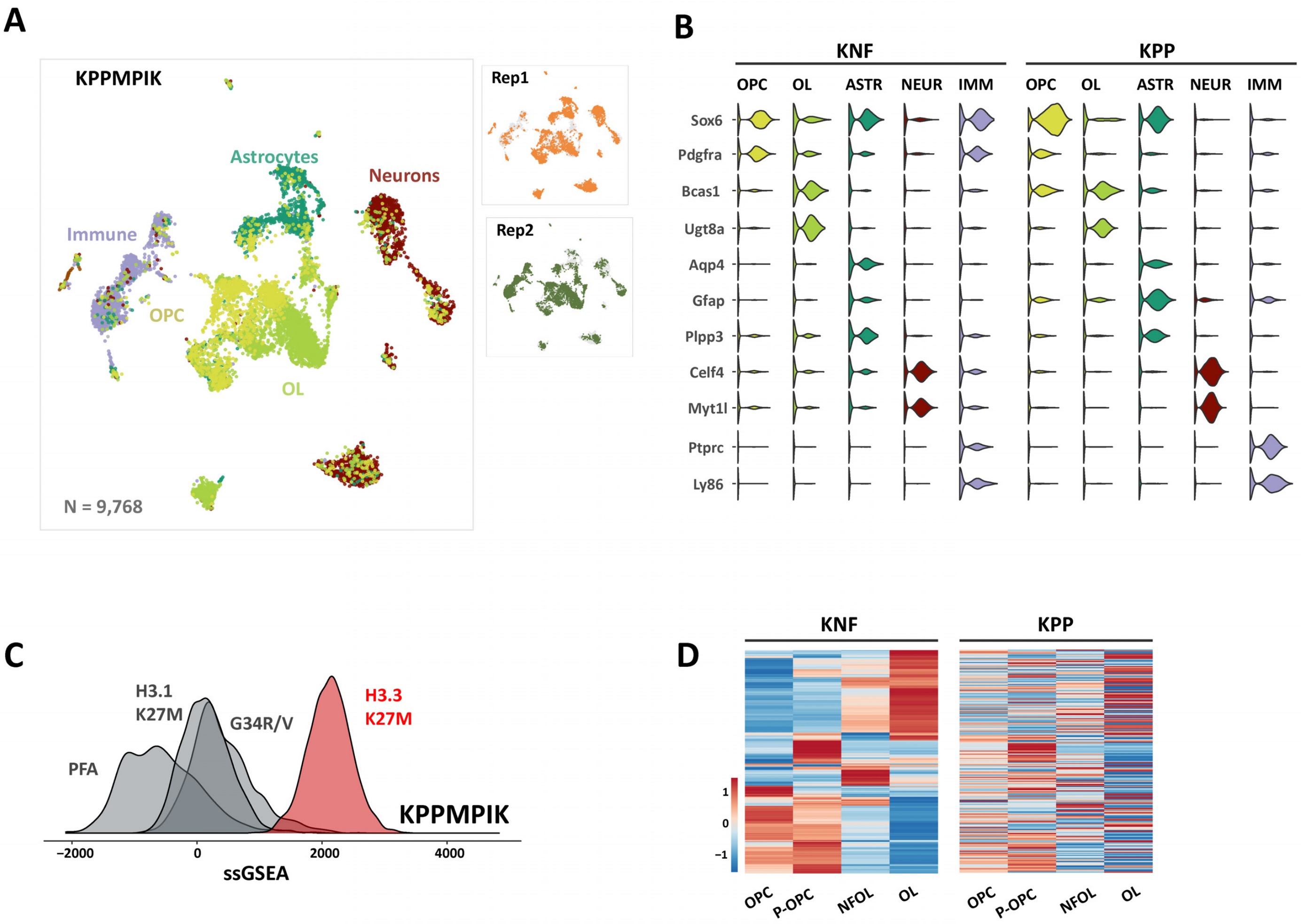

Supplementary Figure 17

A UMAPs of KPPMPIK integration colored by cell type annotation summarized in cell classes (left) and by replicate (right). Cells with low QC as determined from individual sample analysis and cells with no cell type consensus are not plotted. **B** Expression of canonical cell type marker genes in cell type classes, in KNF cells (left) and KPP cells (middle), and KPPMPIK cells (right). **C** Distribution of ssGSEA scores per cell for human tumor signatures in OPC-projected cells of KPPMPIK (N=1,321 cells). Signatures were derived from differential expression of bulk RNA seq data for tumor subtypes (see Methods). **D** Mean inferred TF activity scores for TFs (rows) inferred in both genotypes. Activity scaled by TF shown for clusters 0, 7, 10, and 2 (columns), with predominant cell types of OPC, proliferating OPC, newly-forming oligodendrocytes, and mature oligodendrocytes, respectively. TFs in both genotypes are ordered by hierarchical clustering of KNF activity scores. ASTR, astrocyte; IMM, immune; NEUR, neuron; NFOL, newly-forming oligodendrocyte; OPC, oligodendrocyte precursor cell; OL, oligodendrocyte, P-OPC, proliferating OPC.

Supplementary Figure 18: H3.3^{K27M}, p53^{LOF}

KP

A

Histology

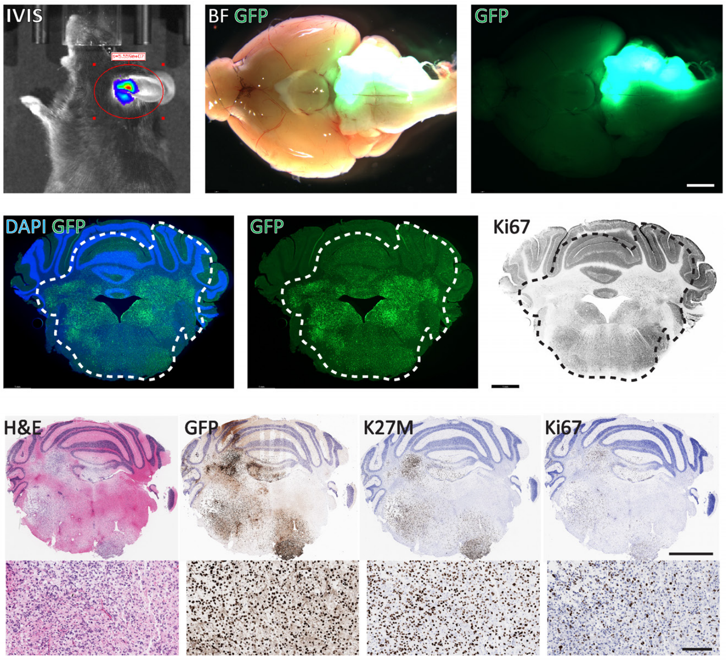

B

Immunochemical characterisation

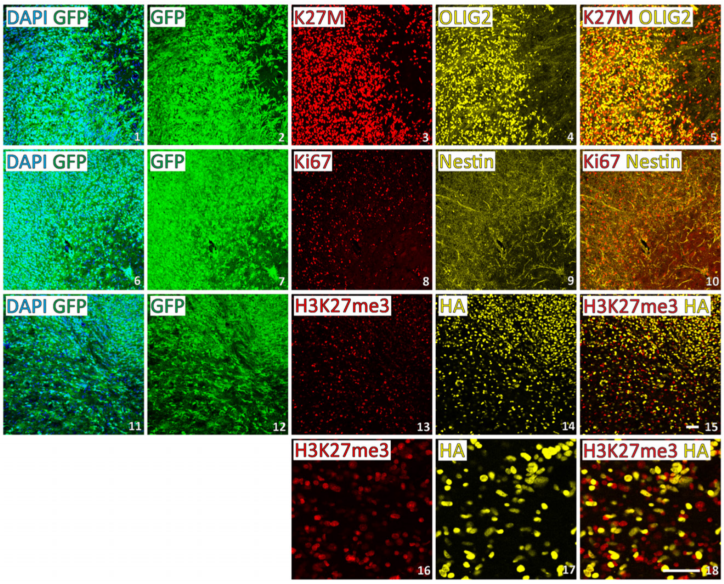

Supplementary Figure 18

A (Top) Bioluminescence imaging and low magnification view of a KP tumor in a symptomatic animal. Scale bars represent 1 mm. (Middle) Coronal section through the tumor and immunofluorescence for DAPI, GFP and Ki67. Scale bars represent 1 mm. (Bottom) Coronal section through the tumor and H&E staining and immunohistochemical detection of GFP, K27M and Ki67. Scale bars represent 2 mm and 200 μm. **B** Immunofluorescence to detect levels of K27M, Olig2, Ki67, Nestin, H3K27me3 and HA in the tumor. Insets below show higher magnification views of H3K27me3 levels in HA^{+} nuclei. Scale bars represent 50 μm.

Supplementary Figure 19: H3.3^{K27M}, FGFR1^{N457K}

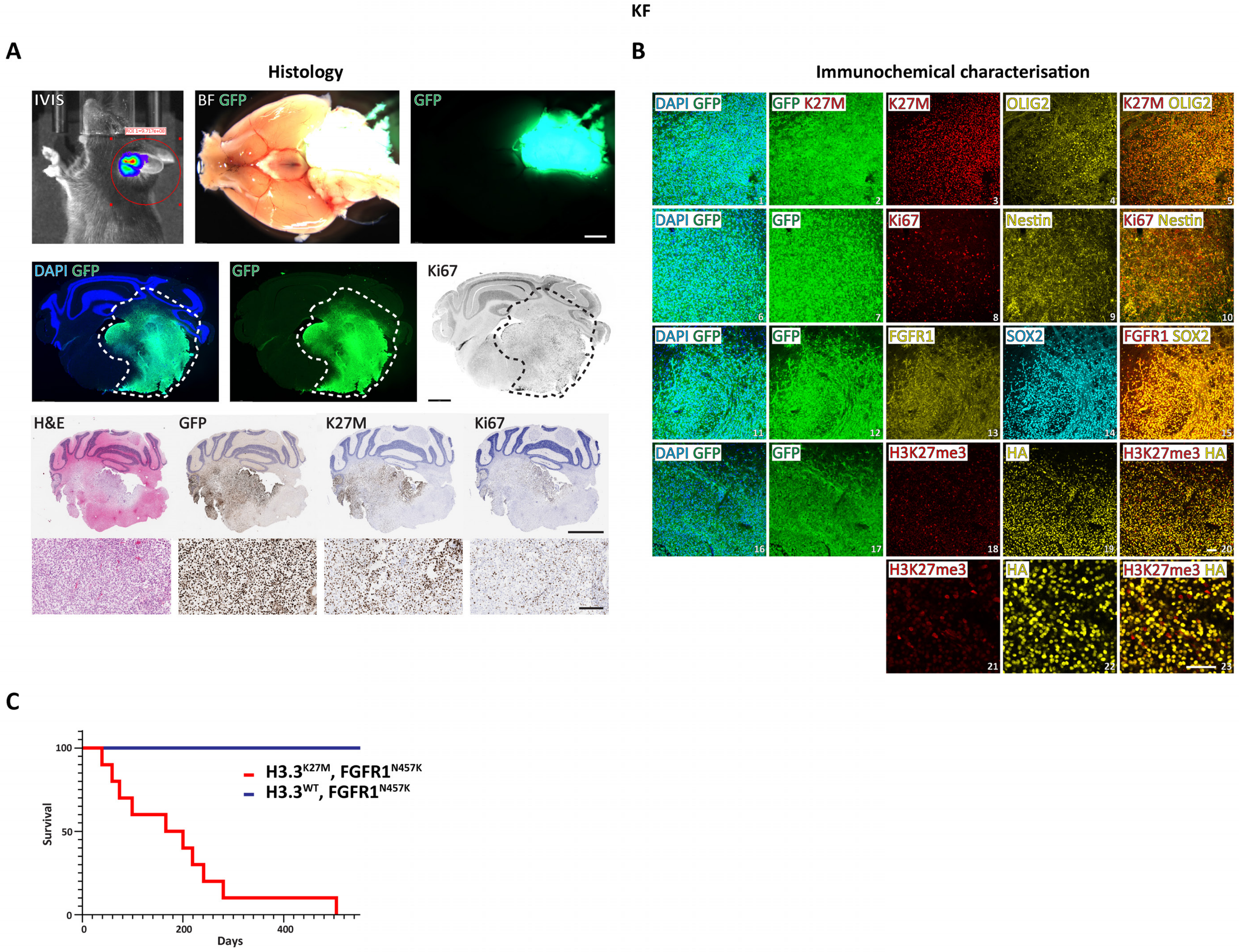

Supplementary Figure 19

A (Top) Bioluminescence imaging and low magnification view of a KF tumor in a symptomatic animal. Scale bars represent 1 mm. (Middle) Coronal section through the tumor and immunofluorescence for DAPI, GFP and Ki67. Scale bars represent 1 mm. (Bottom) Coronal section through the tumor and H&E staining and immunohistochemical detection of GFP, K27M and Ki67. Scale bars represent 2 mm and 200 μm. **B** Immunofluorescence to detect levels of K27M, Olig2, Ki67, Nestin, FGFR1, Sox2, H3K27me3 and HA in the tumor. Insets below show higher magnification views of H3K27me3 levels in HA^{+} nuclei. Scale bars represent 50 μm. **C** Kaplan-Meier survival curves of *in utero* electroporated, tumor-bearing mice carrying different combinations of mutations. KF (n=12), H3.3^{WT}, FGFR1^{N457K} (n=4). Statistical comparisons using the log-rank Mantel-Cox tests are described in Supplementary Table 2.

Supplementary Figure 20: H3.3^{K27M}, NF1^{LOF}

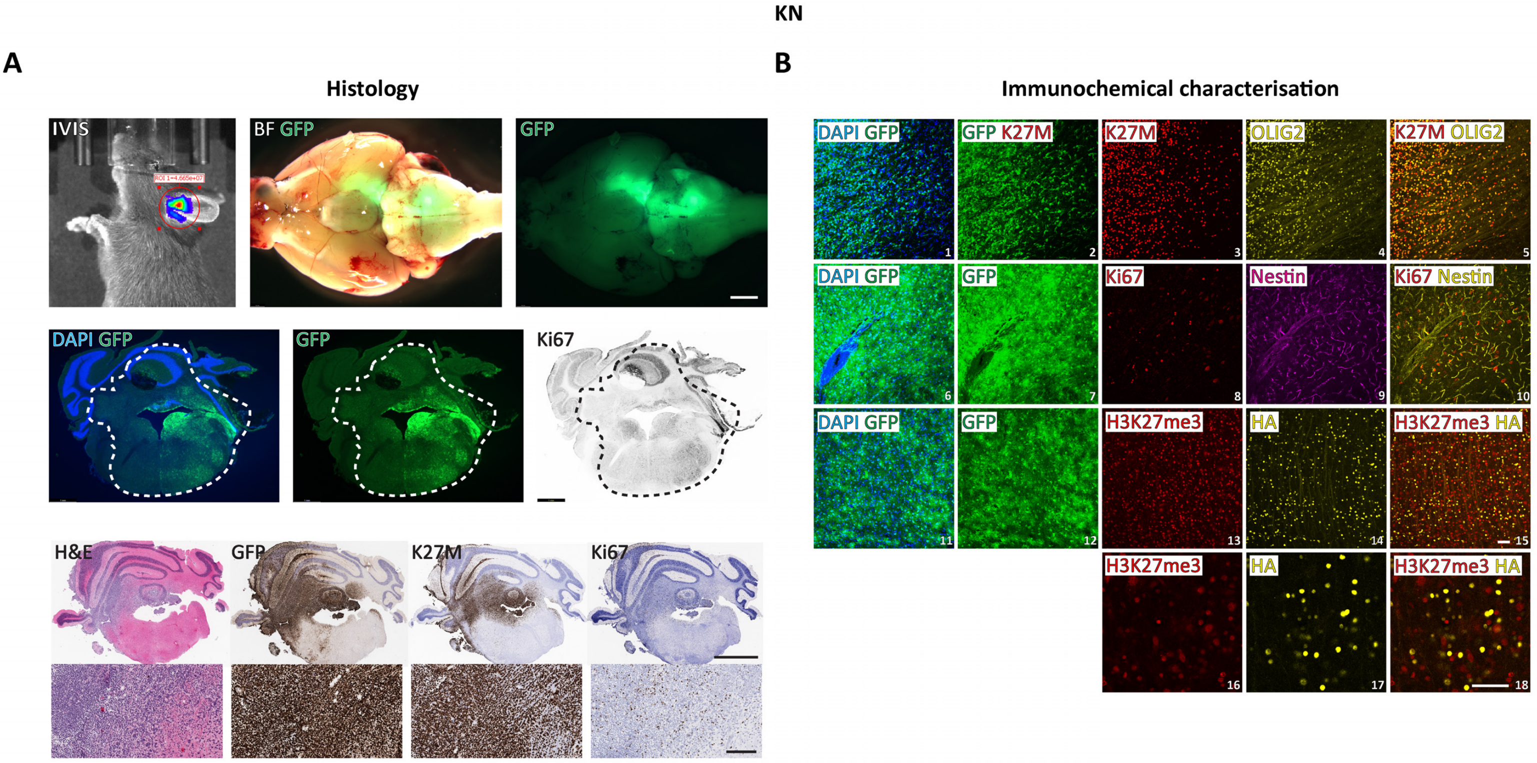

Supplementary Figure 20

A (Top) Bioluminescence imaging and low magnification view of a KN tumor in a symptomatic animal. Scale bars represent 1 mm. (Middle) Coronal section through the tumor and immunofluorescence for DAPI, GFP and Ki67. Scale bars represent 1 mm. (Bottom) Coronal section through the tumor and H&E staining and immunohistochemical detection of GFP, K27M and Ki67. Scale bars represent 2 mm and 200 μm. **B** Immunofluorescence to detect levels of K27M, Olig2, Ki67, Nestin, H3K27me3 and HA in the tumor. Insets below show higher magnification views of H3K27me3 levels in HA^{+} nuclei. Scale bars represent 50 μm.

Supplementary Figure 21: H3.1^{K27M}, p53^{LOF}

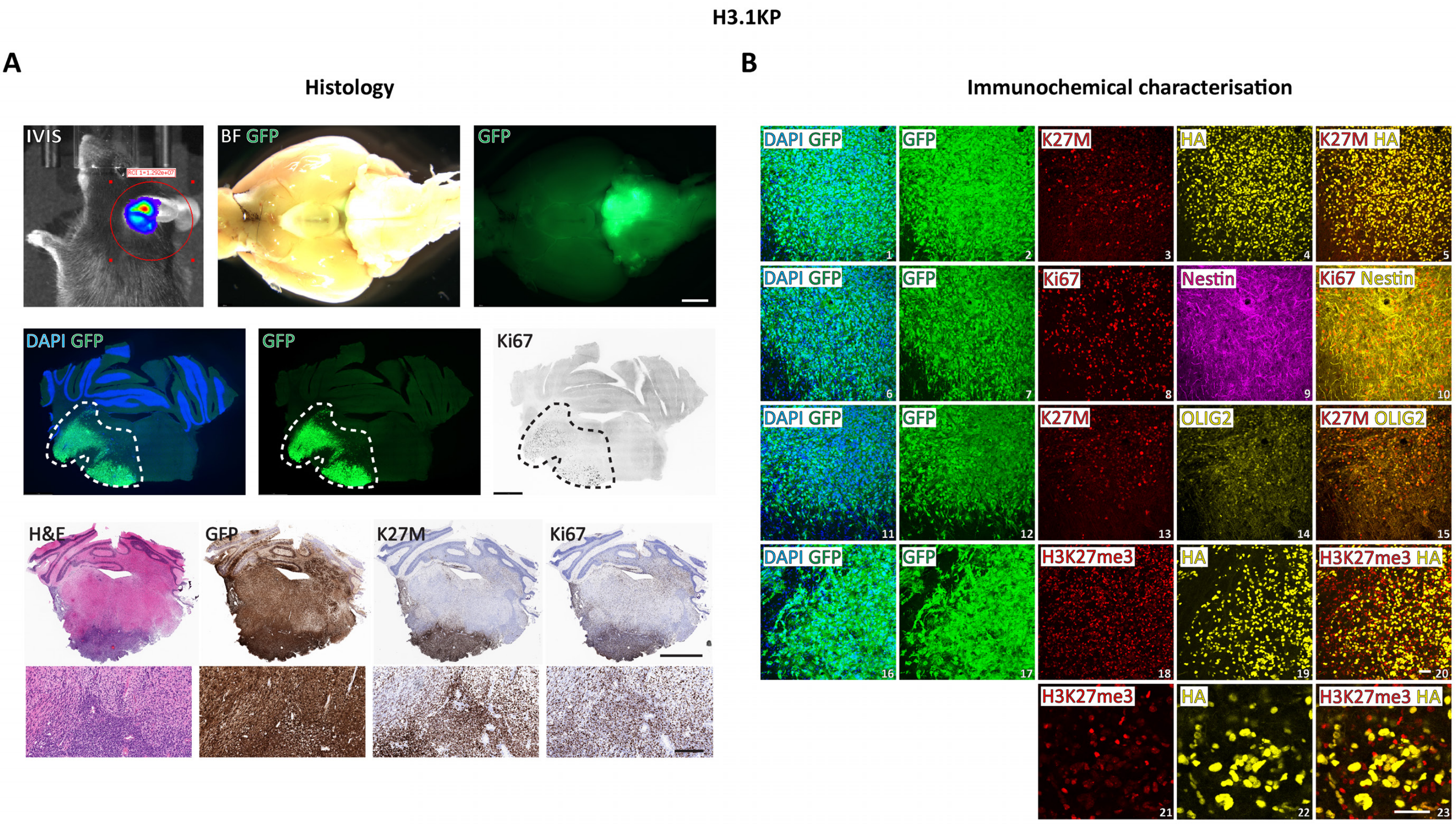

Supplementary Figure 21

A (Top) Bioluminescence imaging and low magnification view of an H3.1KP tumor in a symptomatic animal. Scale bars represent 1 mm. (Middle) Coronal section through the tumor and immunofluorescence for DAPI, GFP and Ki67. Scale bars represent 1 mm. (Bottom) Coronal section through the tumor and H&E staining and immunohistochemical detection of GFP, K27M and Ki67. Scale bars represent 2 mm and 200 μm. **B** Immunofluorescence to detect levels of K27M, Ki67, Nestin, Olig2, H3K27me3 and HA in the tumor. Insets below show higher magnification views of H3K27me3 levels in HA^{+} nuclei. Scale bars represent 50 μm.

Supplementary Figure 22: H3.3^{K27M}, p53^{LOF}, FGFR1^{N457K}

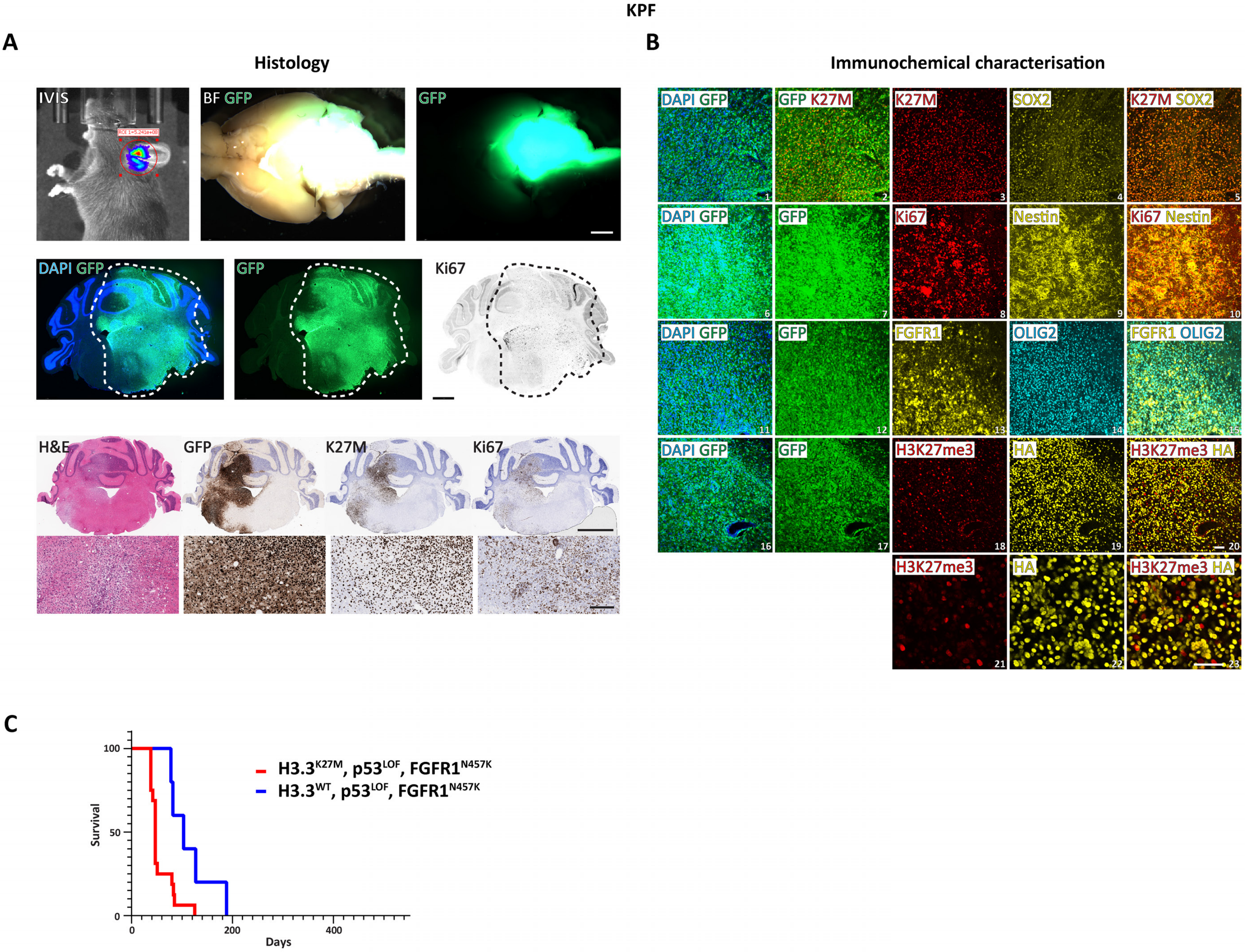

Supplementary Figure 22

A (Top) Bioluminescence imaging and low magnification view of a KPF tumor in a symptomatic animal. Scale bars represent 1 mm. (Middle) Coronal section through the tumor and immunofluorescence for DAPI, GFP and Ki67. Scale bars represent 1 mm. (Bottom) Coronal section through the tumor and H&E staining and immunohistochemical detection of GFP, K27M and Ki67. Scale bars represent 2 mm and 200 μm. **B** Immunofluorescence to detect levels of K27M, Sox2, Ki67, Nestin, FGFR1, Olig2, H3K27me3 and HA in the tumor. Insets below show higher magnification views of H3K27me3 levels in HA^{+} nuclei. Scale bars represent 50 μm. **C** Kaplan-Meier survival curves of *in utero* electroporated, tumor-bearing mice carrying different combinations of mutations. KPF (n=16), H3.3^{WT}, p53^{LOF}, FGFR1^{N457K} (n=5). Statistical comparisons using the log-rank Mantel-Cox tests are described in Supplementary Table 2.

Supplementary Figure 23: H3.3^{K27M}, p53^{LOF}, CCND2^{WT}

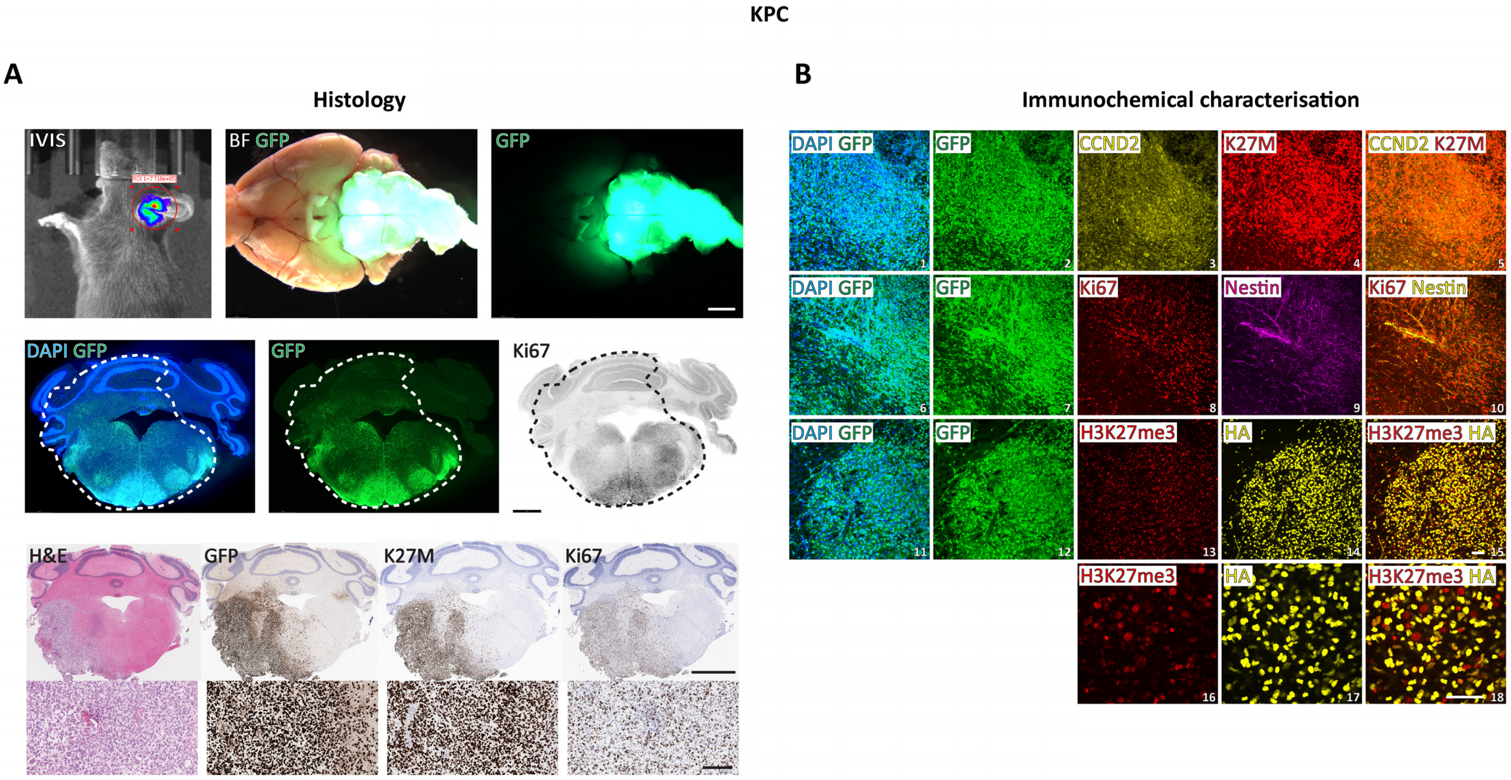

Supplementary Figure 23

A (Top) Bioluminescence imaging and low magnification view of a KPC tumor in a symptomatic animal. Scale bars represent 1 mm. (Middle) Coronal section through the tumor and immunofluorescence for DAPI, GFP and Ki67. Scale bars represent 1 mm. (Bottom) Coronal section through the tumor and H&E staining and immunohistochemical detection of GFP, K27M and Ki67. Scale bars represent 2 mm and 200 μm. **B** Immunofluorescence to detect levels of CCND2, K27M, Ki67, Nestin, H3K27me3 and HA in the tumor. Insets below show higher magnification views of H3K27me3 levels in HA^{+} nuclei. Scale bars represent 50 μm.

Supplementary Figure 24: H3.3^{K27M}, p53^{LOF}, PDGFRA^{D842V}

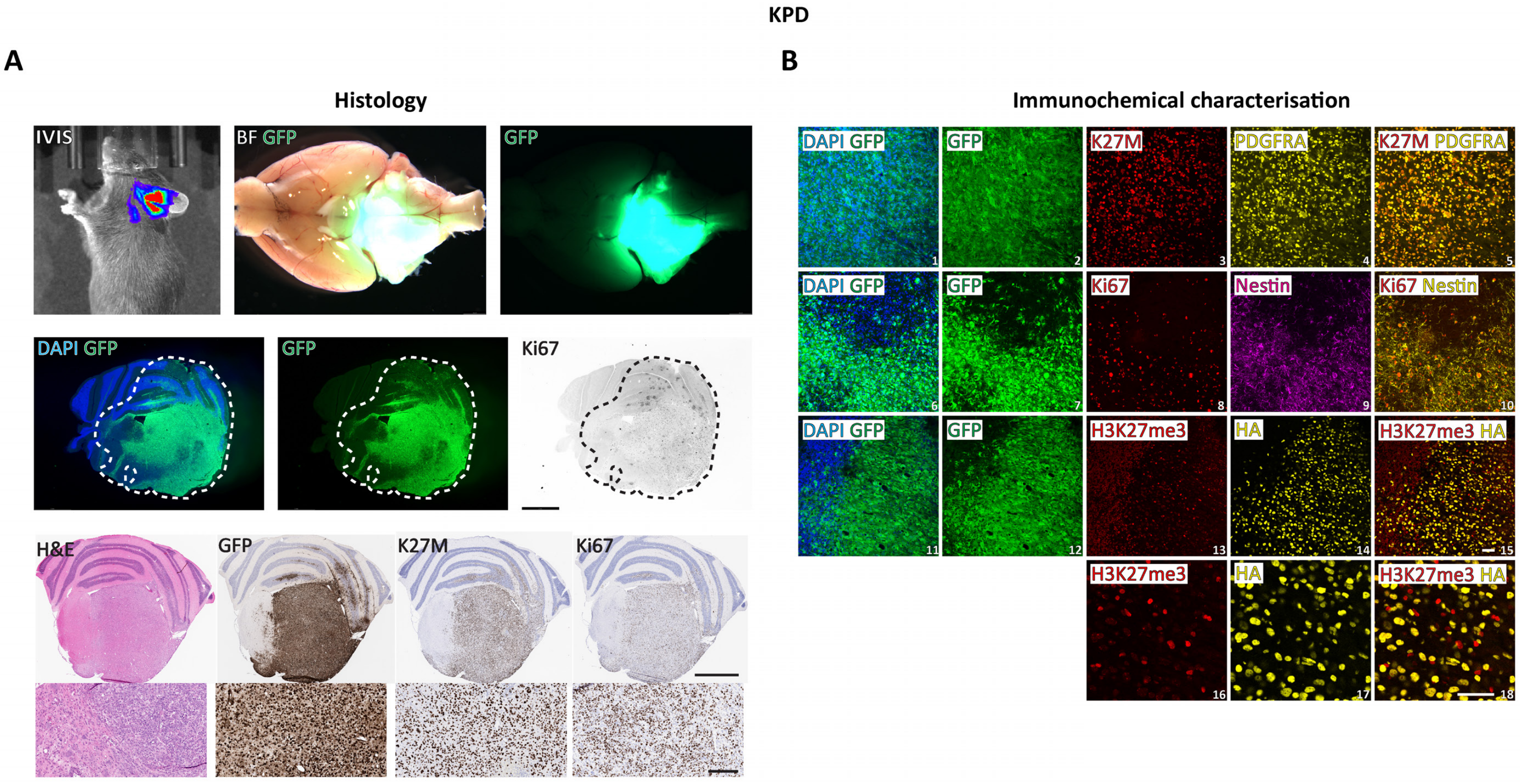

Supplementary Figure 24

A (Top) Bioluminescence imaging and low magnification view of a KPD tumor in a symptomatic animal. Scale bars represent 1 mm. (Middle) Coronal section through the tumor and immunofluorescence for DAPI, GFP and Ki67. Scale bars represent 1 mm. (Bottom) Coronal section through the tumor and H&E staining and immunohistochemical detection of GFP, K27M and Ki67. Scale bars represent 2 mm and 200 μm. **B** Immunofluorescence to detect levels of K27M, PDGFRA, Ki67, Nestin, H3K27me3 and HA in the tumor. Insets below show higher magnification views of H3K27me3 levels in HA^{+} nuclei. Scale bars represent 50 μm.

Supplementary Figure 25: H3.3^{K27M}, p53^{LOF}, ATRX^{LOF}, PDGFRA^{WT}

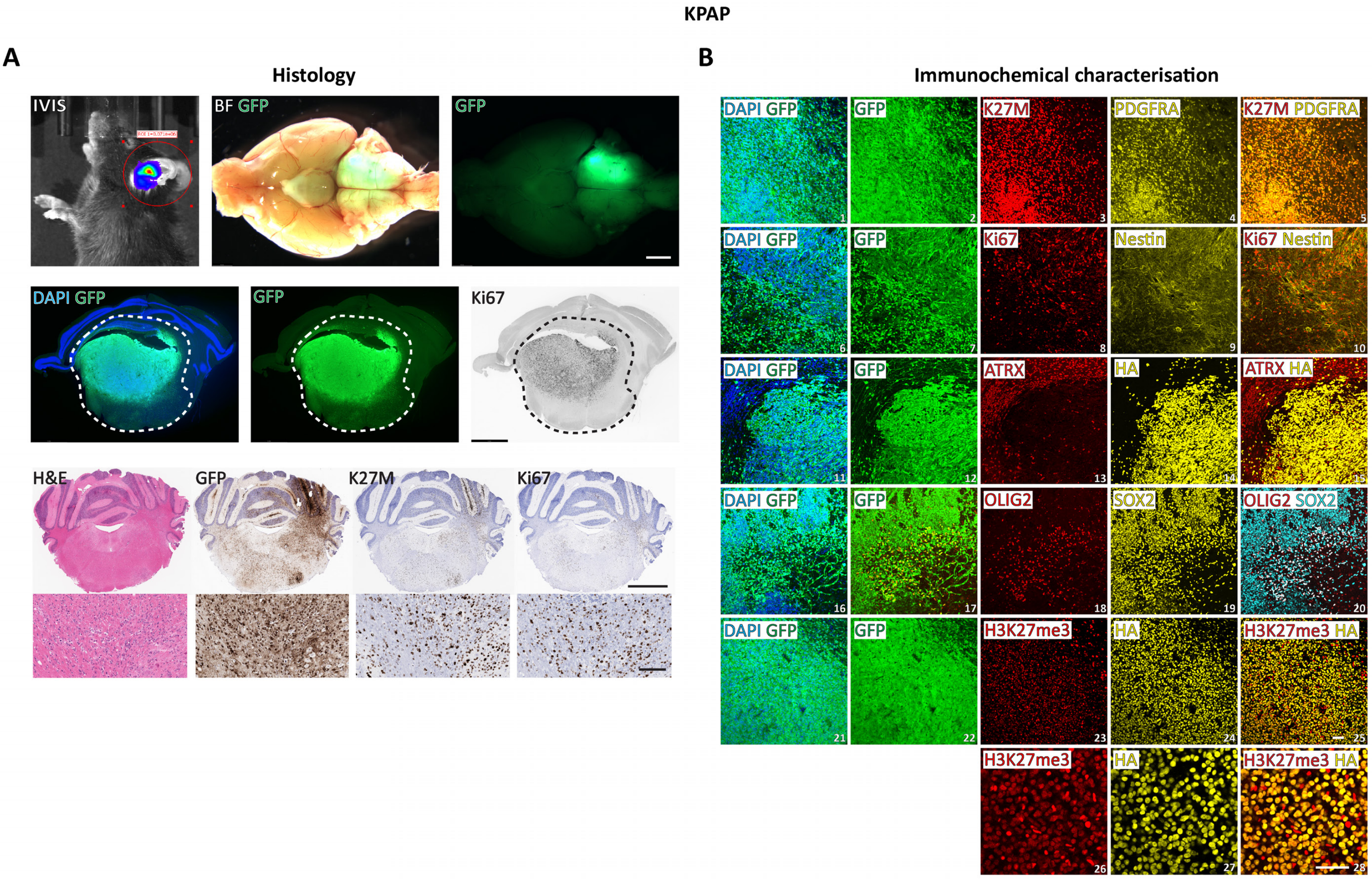

Supplementary Figure 25

A (Top) Bioluminescence imaging and low magnification view of a KPAP tumor in a symptomatic animal. Scale bars represent 1 mm. (Middle) Coronal section through the tumor and immunofluorescence for DAPI, GFP and Ki67. Scale bars represent 1 mm. (Bottom) Coronal section through the tumor and H&E staining and immunohistochemical detection of GFP, K27M and Ki67. Scale bars represent 2 mm and 200 μm. **B** Immunofluorescence to detect levels of K27M, PDGFRA, Ki67, Nestin, ATRX, Olig2, Sox2, H3K27me3 and HA in the tumor. Insets below show higher magnification views of H3K27me3 levels in HA^{+} nuclei. Scale bars represent 50 μm.

Supplementary Figure 26: H3.3^{K27M}, p53^{LOF}, ATRX^{LOF}, PDGFRA^{D842V}

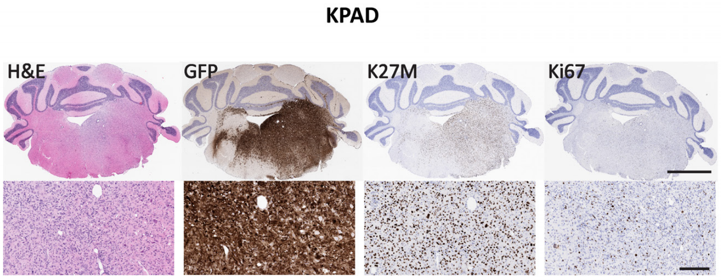

Supplementary Figure 26
A KPAD tumor in a symptomatic animal. Coronal section through the tumor and H&E staining and immunohistochemical detection of GFP, K27M and Ki67. Scale bars represent 2 mm and 200 µm.

Supplementary Figure 27: Drug screening in K27M lines presented as bar graphs

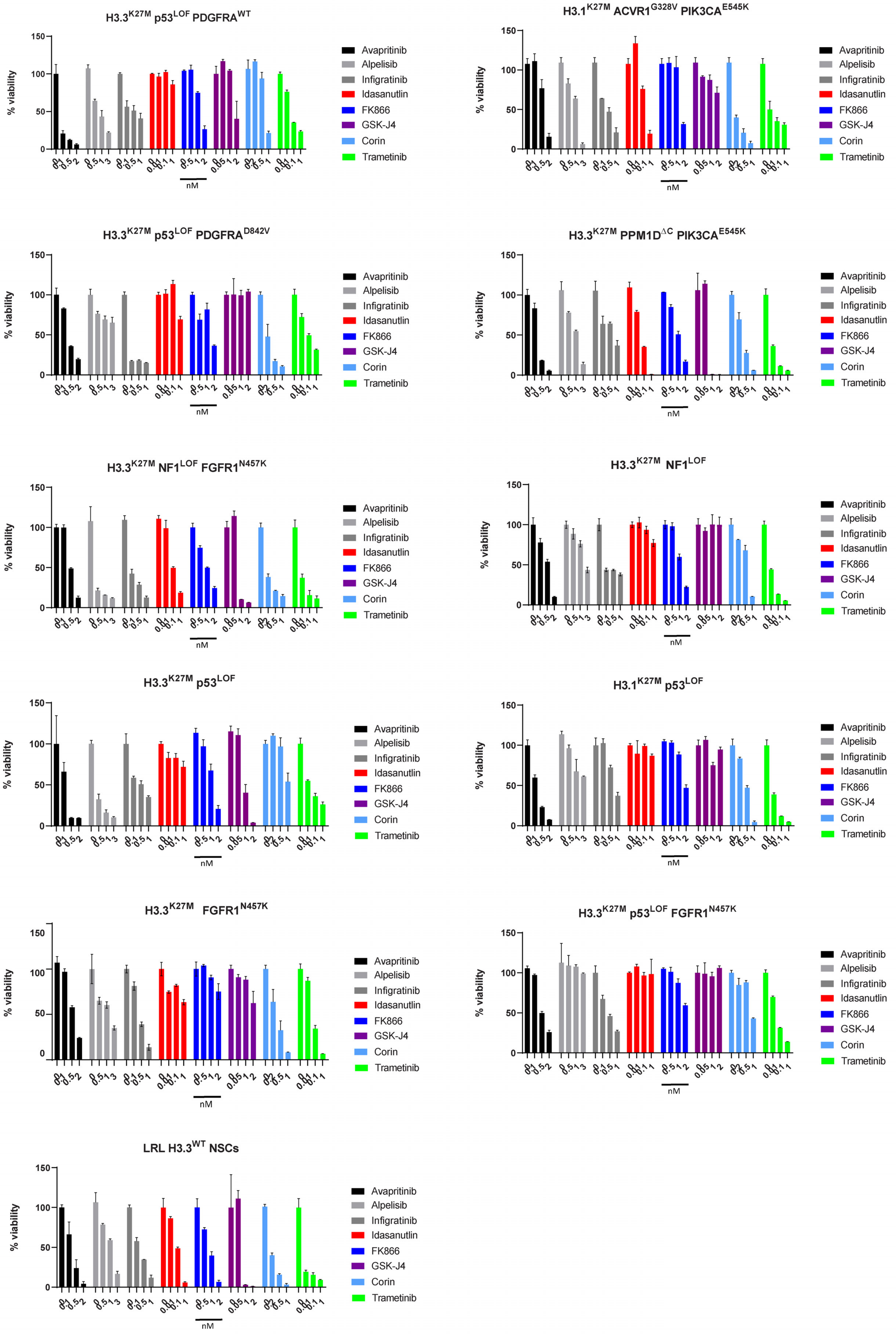

Supplementary Figure 27

Drug screening data represented as bar graphs (also shown in Figure 7A). Note the difference in sensitivity to specific inhibitors between the KPP (PDGFRA^{WT}) and KPD (PDGFRA^{D842V}) lines.
